# Scalable genetic screening for regulatory circuits using compressed Perturb-seq

**DOI:** 10.1038/s41587-023-01964-9

**Published:** 2023-10-23

**Authors:** Douglas Yao, Loic Binan, Jon Bezney, Brooke Simonton, Jahanara Freedman, Chris J. Frangieh, Kushal Dey, Kathryn Geiger-Schuller, Basak Eraslan, Alexander Gusev, Aviv Regev, Brian Cleary

**Affiliations:** 1 Program in Systems, Synthetic, and Quantitative Biology, Harvard University, Cambridge, MA USA; 2 Klarman Cell Observatory, Broad Institute of Harvard and MIT, Cambridge, MA USA; 3 Department of Electrical Engineering and Computer Science, Massachusetts Institute of Technology, Cambridge, MA USA; 4 Harvard T.H. Chan School of Public Health, Boston, MA USA; 5 Genentech, South San Francisco, CA USA; 6 Department of Medical Oncology, Dana-Farber Cancer Institute, Boston, MA USA; 7 Division of Genetics, Brigham and Women’s Hospital, Boston, MA USA; 8 Faculty of Computing and Data Sciences, Boston University, Boston, MA USA; 9 Department of Biology, Boston University, Boston, MA USA; 10 Department of Biomedical Engineering, Boston University, Boston, MA USA; 11 Program in Bioinformatics, Boston University, Boston, MA USA; 12 Biological Design Center, Boston University, Boston, MA USA; 13 Present Address: Department of Genetics, Stanford University School of Medicine, Stanford, CA USA; 14 Present Address: Computational and Systems Biology Program, Sloan Kettering Institute, Memorial Sloan Kettering Cancer Center, New York, NY USA; 15 Present Address: Genentech, South San Francisco, CA USA

**Keywords:** High-throughput screening, Gene expression profiling, High-throughput screening, Gene expression profiling

## Abstract

Pooled CRISPR screens with single-cell RNA sequencing readout (Perturb-seq) have emerged as a key technique in functional genomics, but they are limited in scale by cost and combinatorial complexity. In this study, we modified the design of Perturb-seq by incorporating algorithms applied to random, low-dimensional observations. Compressed Perturb-seq measures multiple random perturbations per cell or multiple cells per droplet and computationally decompresses these measurements by leveraging the sparse structure of regulatory circuits. Applied to 598 genes in the immune response to bacterial lipopolysaccharide, compressed Perturb-seq achieves the same accuracy as conventional Perturb-seq with an order of magnitude cost reduction and greater power to learn genetic interactions. We identified known and novel regulators of immune responses and uncovered evolutionarily constrained genes with downstream targets enriched for immune disease heritability, including many missed by existing genome-wide association studies. Our framework enables new scales of interrogation for a foundational method in functional genomics.

## Abstract (web summary)

Compressed Perturb-seq incorporates compressed sensing to genetic screening for scalable discovery of genetic interactions.

## Main

Pooled perturbation screens with high-content readouts ranging from single-cell RNA sequencing (Perturb-seq)^1–4^ to imaging-based spatial profiling^5–7^ are now enabling systematic studies of the regulatory circuits that underlie diverse cell phenotypes. Perturb-seq has been applied to various model systems, leading to insights about diverse cellular processes, including the innate immune response^2^, in vivo effects of autism risk genes in mice^8^ and organoids^9,10^ and genome-scale effects on aneuploidy, differentiation and RNA splicing^11^. Integrating data from population-level genetic screens has also elucidated human disease mechanisms^12^.

However, owing to the large number of genes in the genome, large-scale Perturb-seq screens are still prohibitively expensive and are often limited by the number of available cells, especially for primary cell systems^13^ and in vivo niches^8^. In addition, the exponentially larger number of possible genetic interactions makes it impossible to conduct exhaustive combinatorial screens for genetic interactions using existing approaches, so current Perturb-seq studies of genetic interactions are very modest and focused^14^. Several approaches have been developed to improve the efficiency of single-cell RNA sequencing (scRNA-seq) and/or Perturb-seq, including overloading droplets with multiple pre-indexed cells (SciFi-seq^15^) or pooling multiple guides within cells^16^. However, pre-indexing requires an additional laborious and complex experimental step, and guide-pooling has only been used to study *cis* and not *trans* effects of perturbations.

We propose an alternative approach to greatly increase the efficiency and power of Perturb-seq for both single and combinatorial perturbation screens, inspired by theoretical results from compressed sensing^17–19^ that apply to the sparse and modular nature of regulatory circuits in cells. To elaborate, perturbation effects tend to be ‘sparse’, in that most perturbations affect only a small number of genes or co-regulated gene programs^2,20^. In this scenario, rather than assaying each perturbation individually, we can measure a much smaller number of random combinations of perturbations (forming what we call ‘composite samples’) and accurately learn the effects of individual perturbations from the composite samples using sparsity-promoting algorithms. Moreover, with certain types of composite samples, we can efficiently learn both first-order effects (that is, from single-gene perturbations) and higher-order genetic interaction effects from the same data. We previously showed that experiments that measure random compositions of the underlying biological dataset can greatly increase the efficiency of measuring expression profiles^21^ and imaging transcriptomics^22^.

In the present study, we developed two experimental strategies to generate composite samples for Perturb-seq screens, and we introduce here an inference method, Factorize-Recover for Perturb-seq (FR-Perturb), to learn individual perturbation effects from composite samples. We applied our approach to 598 genes in a human macrophage cell line treated with bacterial lipopolysaccharide (LPS). By comparing compressed Perturb-seq to conventional Perturb-seq conducted in the same system, we demonstrate the enhanced efficiency and power of our approach for learning single perturbation effects and second-order genetic interactions. We derive insights into immune regulatory functions and illustrate their connection to human disease mechanisms by integrating data from genome-wide association studies (GWASs) and expression quantitative trait loci (eQTL) studies.

## Results

### A compressed sensing framework for perturbation screens

**Fig. 1:**
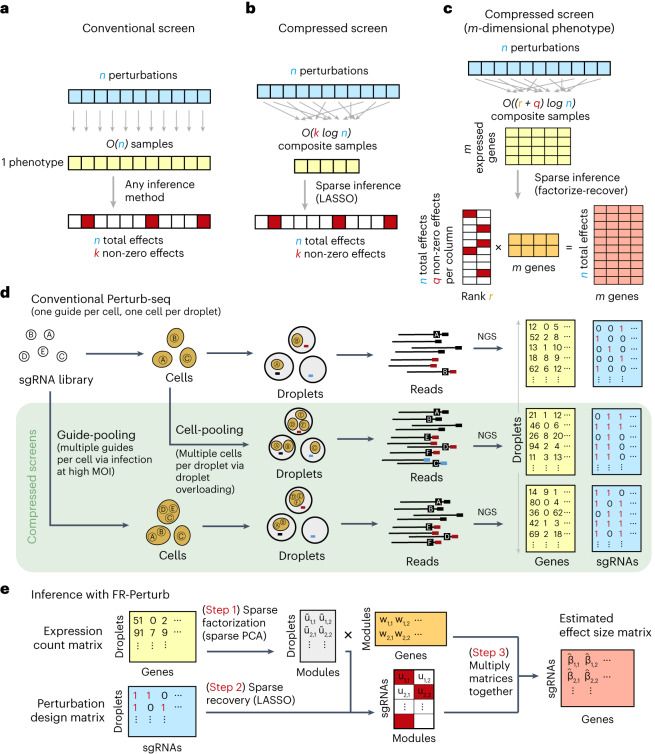
Framework for compressed Perturb-seq. **a**, Schematic for conventional perturbation screen with single-valued phenotype. Each sample (yellow) receives a single perturbation (blue). The required number of samples scales linearly with the number of perturbations, as captured by the *O*(*n*) term. **b**, Schematic for compressed perturbation screen with single-valued phenotype. Each ‘composite’ sample (yellow) represents a random combination of perturbations (blue). The required number of samples scales sub-linearly with the number of perturbations given the following: (1) the effects of the perturbations are sparse (that is, *k* increases more slowly than *n*), and (2) sparse inference (typically LASSO) is used to infer the effects from the composite sample phenotypes. **c**, Schematic for compressed perturbation screen with high-dimensional phenotype, which is the main use case for Perturb-seq. The required number of samples scales sub-linearly with the number of perturbations given the following: (1) the effects of the perturbations are sparse and act on a relatively small number of groups of correlated genes (that is, *q* and *r* increase more slowly than *n*), and (2) sparse inference (namely the ‘factorize-recover’ algorithm^23^) is used to infer the effects from the composite sample phenotypes. **d**, Two experimental strategies for generating composite samples for Perturb-seq. Both ‘cell-pooling’ and ‘guide-pooling’ change one step of the conventional Perturb-seq protocol. The result is a sample whose phenotype corresponds to a random linear combination of the phenotypes of samples from the conventional Perturb-seq screen. **e**, Schematic of computational method used to infer perturbation effects from composite sample phenotypes, based on the ‘factorize-recover’ algorithm^23^. NGS, next-generation sequencing.

In conventional Perturb-seq, each cell in a pool receives one or more genetic perturbations. Each cell is then profiled for the identity of the perturbation(s) and the expression levels of *m* ≈ 20,000 expressed genes. Our goal is to infer the effect sizes of *n* perturbations on the phenotype, which can be the entire gene expression profile (*n* × *m* matrix) or an aggregate multi-gene phenotype^2,3,11^, such as an expression program or cell state score (length − *n* vector). In both cases, we need *O*(*n*) samples to learn the effects of *n* perturbations (Fig. 1a) (where sample replicates introduce a constant factor that is subsumed under the big O notation), such that the number of samples scales linearly with the number of perturbations.

Based on the theory of compressed sensing^17^, there exist conditions under which far fewer than *O*(*n*) samples are sufficient to learn the effects of *n* perturbations. In general, if the perturbation effects are sparse (that is, relatively few perturbations affect the phenotype) or are sparse in a latent representation (that is, perturbations tend to affect relatively few latent factors that can be combined to ‘explain’ the phenotype), then we can measure a small number of random composite samples (comprising ‘linear combinations’ of individual sample phenotypes) and decompress those measurements to infer the effects of individual perturbations. Composite samples can be generated either by randomly pooling perturbations in individual cells or by randomly pooling cells containing one perturbation each (see below).

**Extended Data Fig. 1:**
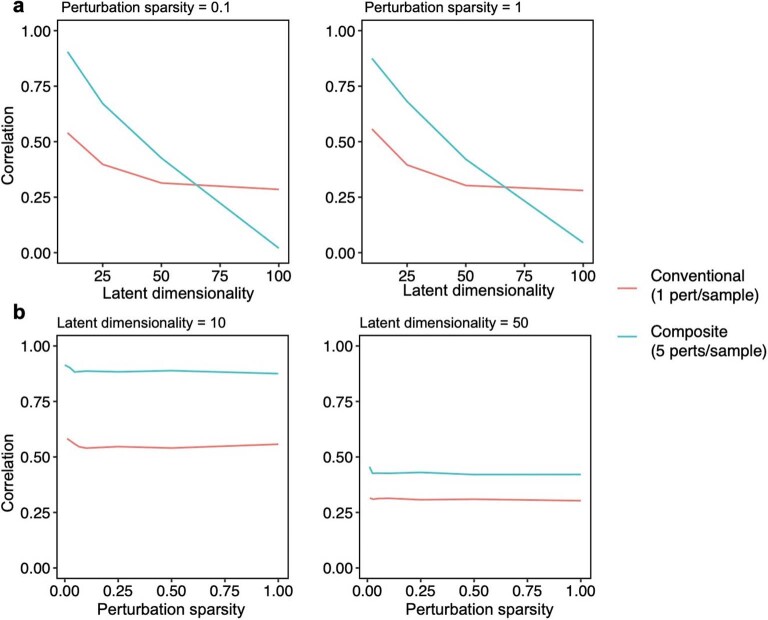
Performance of compressed Perturb-seq in simulations with different effect size structure. Effect sizes were simulated for 100 perturbations on 10,000 genes by separately simulating factor matrices, comprising a (1) 100 perturbation x module ‘activity’ matrix and (2) module x 10,000 gene ‘dictionary’ matrix, then multiplying the matrices together to obtain the final effect size matrix. Entries for both factor matrices were drawn from N(0, 1). The latent dimensionality (corresponding to r in the main text) of the final matrix was set by varying the number of modules (that is columns of the activity matrix or rows of the dictionary matrix). The perturbation sparsity (corresponding to q in the main text) was set by randomly setting a given proportion of entries in the module activity matrix to zero. Samples were generated by taking random rows (or sums of random combinations of rows) of the perturbation-by-gene effect size matrix, with the number of rows represented per sample set to 1 for conventional samples or 5 for composite samples. Noise from N(0, 9) was added to all samples to generate phenotypes with 10% signal and 90% noise for the 1 perturbation/sample scenario (plausible for single-cell expression data). Unless otherwise specified, inference was performed using the Factorize-Recover algorithm. (**a**) Correlation of inferred vs. true effects (Y-axis) when varying the latent dimensionality r of the perturbation effect size matrix (X-axis). q was fixed at 0.1 (left) or 1 (right). (**b**) Correlation of inferred vs. true effects (Y-axis) when varying the perturbation sparsity q (that is the proportion of nonzero entries in the module activity matrix; X-axis). r was fixed at 10 (left) or 50 (right).

The number of required composite samples depends on whether the phenotype is single valued or high dimensional. When the phenotype is single valued (for example, fitness), *O*(*k* log *n*) composite samples suffice to accurately recover the effects of *n* perturbations^18,19^, where *k* is the number of non-zero elements among the *n* perturbation effects (Fig. 1b). When most perturbations do not affect the phenotype, *k* grows more slowly than *n*, and the number of required composite samples scales logarithmically or, at worst, sub-linearly with the number of perturbations. Meanwhile, when the phenotype is an *m*-dimensional gene expression profile, an efficient approach involves inferring effects on latent expression factors and then reconstructing the effects on individual genes from these factors using the ‘factorize-recover’ algorithm^23^. This approach requires $O\left(\left(q+r\right)\log n\right)$ composite samples, where *r* is the rank of the *n* × *m* perturbation effect size matrix (that is, the maximum number of its linearly independent column vectors), and *q* is the maximum number of non-zero elements in any column of the left matrix of the factorized effect size matrix (Fig. 1c). In our case, *r* is the number of distinct groups of ‘co-regulated’ genes whose expression changes concordantly in response to any perturbation, and *q* is the maximum number of ‘co-functional’ perturbations with non-zero effects on any individual module. Due to the modular nature of gene regulation^20,24,25^, *r* and *q* are expected to remain small when *n* increases. Indeed, we observed a relatively small number of co-functional and co-regulated gene groups (small *q* and *r*, respectively, relative to *n*) in previous Perturb-seq screens in various systems^2,13^. Thus, the number of required composite samples will scale logarithmically or, at worst, sub-linearly with *n*, leading to much fewer required samples than the conventional approach with large *n*. In simulations, this result held across a wide range of plausible values for *q* and *r* (Extended Data Fig. 1). We provide rough estimates of *q* and *r* from our own screens (see below) in the Supplementary Note, section 1.

### Experimentally generating composite samples

We generated composite samples for compressed Perturb-seq either by randomly pooling cells containing one perturbation each in overloaded scRNA-seq droplets^15^ (‘cell-pooling’) or by randomly pooling guides in individual cells via infection with a high multiplicity of infection (MOI)^2,16^ (‘guide-pooling’) (Fig. 1d). Under certain assumptions, the resulting expression counts in each droplet from either method represent a random linear combination of log fold change effect sizes of guides. When cell-pooling, the expression counts in a given droplet are proportional to the average expression counts of the cells in the droplet, which can then be modeled in terms of log fold change effect sizes of the guides in each cell (Methods). When guide-pooling, the expression counts in a given droplet can also be modeled as the sum of log fold change effect sizes (Methods), although this requires the non-trivial assumption that the effect sizes of guides tend to combine additively in log expression space when multiple guides are present in the same cell. Although higher-order genetic interaction effects can, in theory, bias lower-order effect size estimates in guide-pooled data, we note that only a large imbalance in the direction and/or magnitude of higher-order interaction effects across many perturbations will lead to such biases, and that, even in this scenario, many of the lower-order effects can still be accurately estimated (Supplementary Note, section 2).

Either of the two methods described above can be used to learn the same underlying perturbation effects, but each has different strengths and limitations (Discussion). Guide-pooling has a key benefit over cell-pooling, in that the generated data can be used to estimate both first-order effects and higher-order genetic interactions (with appropriate sample sizes and explicit interaction terms in the model) (Methods). In later analyses, we illustrate the feasibility of estimating second-order effects from guide-pooled data.

### FR-Perturb infers effects from compressed Perturb-seq

To infer perturbation effects from the composite samples, we devised a method called FR-Perturb based on the ‘factorize-recover’ algorithm^23^ (Methods). FR-Perturb first factorizes the expression count matrix with sparse factorization (that is, sparse principal component analysis (PCA)), followed by sparse recovery (that is, least absolute shrinkage and selection operator (LASSO)) on the resulting left factor matrix comprising perturbation effects on the latent factors. Finally, it computes perturbation effects on individual genes as the product of the left factor matrix from the recovery step with the right factor matrix (comprising gene weights in each latent factor) from the first factorization step (Fig. 1e and Methods). Because FR-Perturb uses penalized regression, it is not guaranteed to be unbiased. We obtained *P* values and false discovery rates (FDRs) for all effects by permutation testing (Methods). In later analyses, we evaluated FR-Perturb by comparing it to existing inference methods for Perturb-seq, namely elastic net regression^2^ and negative binomial regression^16^.

### Compressed Perturb-seq screens of the LPS response

**Fig. 2:**
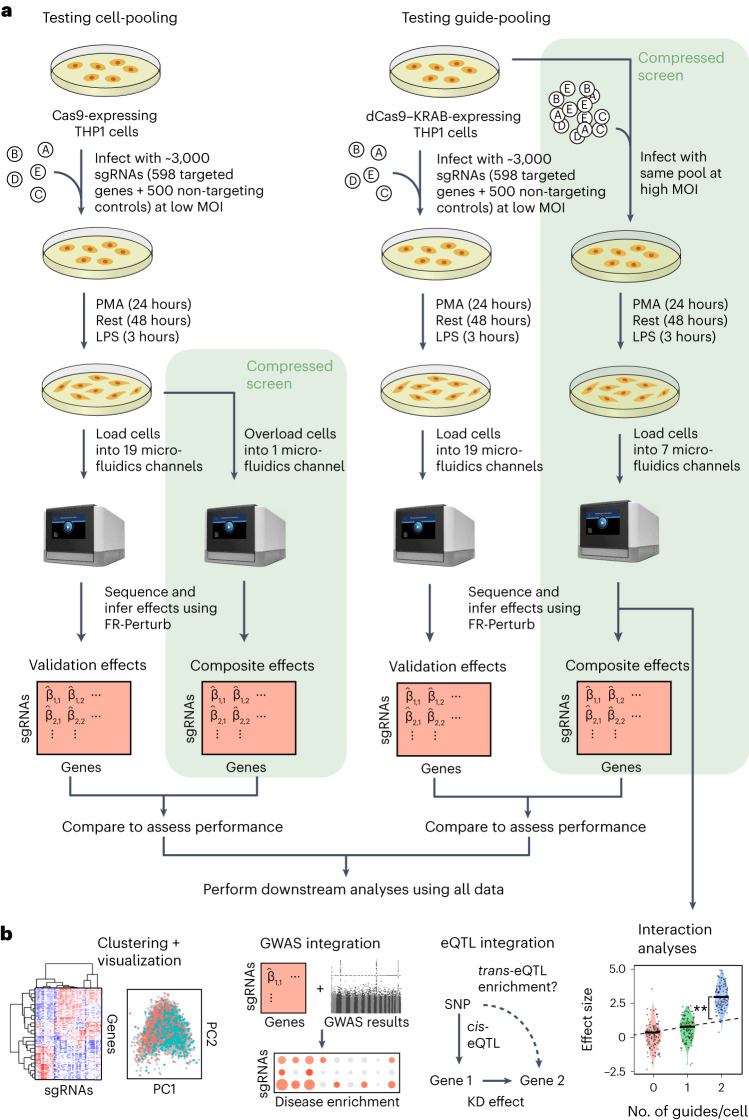
Experimental overview. **a**, Outline of experiments used to test and validate cell-pooling (left) and guide-pooling (right). **b**, Downstream analyses performed using perturbation effects from all experiments.

**Extended Data Fig. 2:**
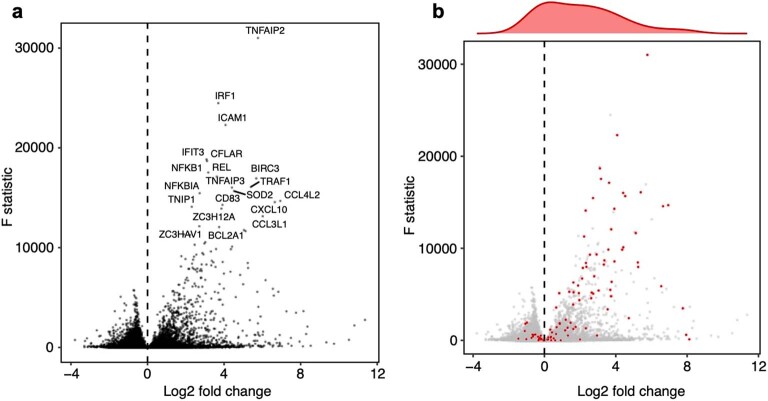
Analysis of pre-stimulated cells. Volcano plots showing the log2 fold changes (x-axis) and F statistics (y-axis) of all genes from differential expression analysis of pre-stimulated vs. LPS-stimulated cells. (**a**) Top 20 most significantly differentially expressed genes are labeled. (**b**) Same data as **a**, but instead the top 100 genes (based on the number of perturbations that significantly modulate them) are highlighted in red. Density plot shows the distribution of log2 fold changes of these 100 genes.

We implemented and evaluated compressed Perturb-seq in the response of THP1 cells (a human monocytic leukemia cell line) to stimulation with LPS when either pooling cells or pooling guides (Fig. 2a,b). In each case, we also performed conventional Perturb-seq, targeting the same genes in the same system for comparison. We selected 598 genes to be perturbed from seven mostly non-overlapping immune response studies (Supplementary Table 1), including genes with roles in the canonical LPS response pathway (34 genes); GWAS for inflammatory bowel disease (IBD) (79 genes) and infection (106 genes); Mendelian immune diseases from the Online Mendelian Inheritance in Man (OMIM) database with keywords for ‘bacterial infection’ (85 genes) and ‘NF-κB’ (102 genes); a previous genome-wide screen for effects on tumor necrosis factor (TNF) expression in mouse bone-marrow-derived dendritic cells (BMDCs)^26^ (93 genes); and genes with large genetic effects in *trans* on gene expression from an eQTL study in patient-derived macrophages stimulated with LPS^27^ (79 genes) (Methods and Supplementary Fig. 1). We designed four single guide RNAs (sgRNAs) for each gene and 500 each of non-targeting or safe-targeting control sgRNAs, resulting in a total pool of 3,392 sgRNAs (Methods). We introduced the sgRNAs into THP1 cells via a modified CROP-seq vector^4^ (Methods). After transduction and selection, we treated cells with PMA for 24 h and grew them for another 48 h as they differentiated into a macrophage-like state^28^, and then we treated them with LPS for 3 h before harvesting for scRNA-seq (Methods). As a baseline, we also collected scRNA-seq data for genetically perturbed cells before stimulation (that is, no LPS treatment) (see Supplementary Note, section 3, and Extended Data Fig. 2 for analysis). For our cell-pooled screen, we used CRISPR–Cas9 to knock out genes^2^, whereas, for our guide-pooled screen, we used CRISPR interference (CRISPRi) with dCas9–KRAB to knock down gene expression^1^ (Fig. 2a) to avoid cellular toxicity due to multiple double-stranded breaks in individual cells^29^.

**Fig. 3:**
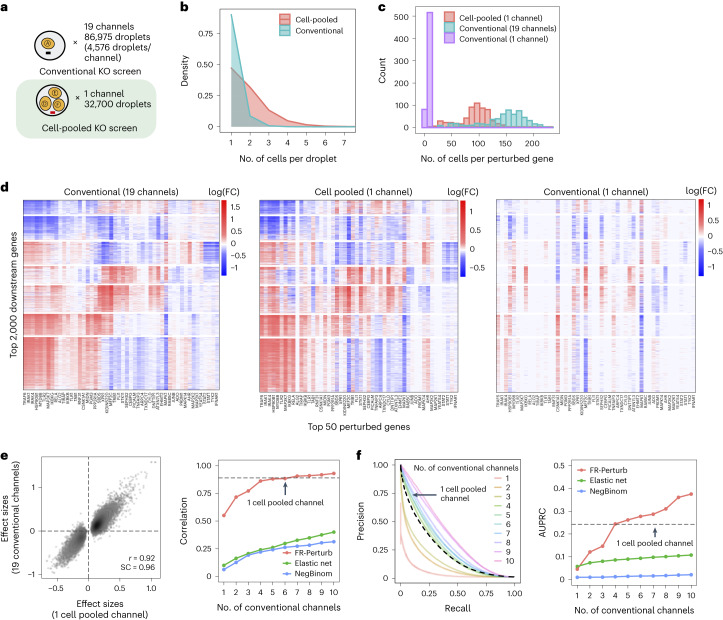
Evaluating cell-pooled Perturb-seq versus conventional Perturb-seq. **a**, Number of channels and droplets from the conventional validation screen (top) and the cell-pooled screen (bottom). **b**, Distribution of droplets based on the number of cells they contain for the cell-pooled and conventional screens. **c**, Distribution of the number of cells containing a guide targeting each perturbed gene in the cell-pooled screen and conventional screen (19 channels = full screen, 1 channel = matching number of channels from cell-pooled screen). **d**, Heat maps of the top effect sizes (inferred with FR-Perturb) from the conventional screen (left), with the same effect sizes shown for the cell-pooled screen (middle) and one equivalent channel of the conventional screen (right). *x* axis: top 50 perturbed genes, based on their average magnitude of effect on all 17,552 downstream genes. *y* axis: top 2,000 downstream genes, based on the average magnitude of effects of all 598 perturbed genes acting on them. Rows and columns are clustered based on hierarchical clustering in the leftmost plot. For the left plot, all effects with FDR *q* > 0.2 are whited out (*q* value threshold relaxed to 0.5 for the middle and right plots). **e**, Left, scatter plot of all significant effects (q < 0.05; *n* = 19,909) from the cell-pooled screen (*x* axis) versus the same effects in the conventional screen (*y* axis). Effects represent log fold changes in expression relative to control cells. *r*, Pearson’s correlation coefficient; SC, sign concordance. Right, held-out validation accuracy of top 19,909 effects (*y* axis; Pearson’s correlation with validation dataset) from the downsampled conventional screen (*x* axis) and the cell-pooled screen (dotted line). The same inference method is used to estimate effects in both the downsampled conventional data and validation data. The effects from the cell-pooled screen are estimated using FR-Perturb only (see Extended Data Fig. 3d for results using other methods). **f**, Left, precision-recall curves computed from downsampled conventional screen and cell-pooled screen (dotted line). True positives = all significant effects (*n* = 79,100) from the held-out validation dataset. The classification threshold being varied (*x* axis) is the significance (that is, *P* value) of the effects. All effects displayed are learned using FR-Perturb. Right, AUPRCs (*y* axis) computed from the downsampled conventional experiment when varying the number of channels (*x* axis). FC, fold change.

By design, the two compressed screens were substantially smaller than their corresponding conventional screens. In the cell-pooled screen, we analyzed a single channel of droplets (10x Genomics; Methods) overloaded with 250,000 cells, whereas, for the corresponding conventional Perturb-seq screen, we analyzed 19 channels at normal loading. We sequenced the library from the overloaded channel to a depth of four-fold more reads than a conventional channel to account for the larger number of non-empty droplets and greater expected RNA content per droplet. After quality control, there were 32,700 droplets containing at least one sgRNA from the overloaded channel (versus 4,576 droplets per channel for a total of 86,954 droplets from the conventional screen) (Fig. 3a), with a mean of 1.86 sgRNAs per non-empty droplet (conventional: 1.11) (Fig. 3b) and a mean of 90 droplets containing a guide for each perturbed gene (conventional: 144) (Fig. 3c). We observed 14,987 total genes with measured expression (conventional: 17,552). Thus, the cell-pooled screen had more than seven times the number of non-empty droplets per channel compared to the conventional screen; considering library preparation and sequencing costs, it was approximately eight times cheaper.

**Fig. 4:**
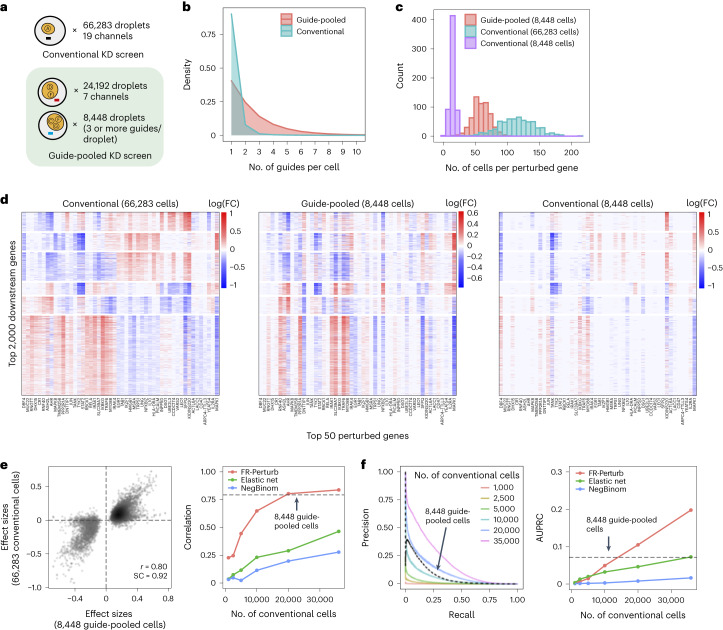
Evaluating guide-pooled Perturb-seq versus conventional Perturb-seq. **a**, Number of channels and droplets from the conventional validation screen (top) and the guide-pooled screen (bottom). We focused our analysis on the subset of 8,448 droplets from the guide-pooled screen with at least three guides per droplet. **b**, Distribution of cells based on the number of guides that they contain for the full guide-pooled and conventional screens. In practice, we only directly measured the number of guides per droplet rather than guides per cell, but these quantities are equivalent given one cell per droplet. **c**–**f**, See captions for Fig. 3c–f. These analyses were conducted in an identical fashion, with the only difference being that the screens are downsampled based on cell count rather than channel count. FC, fold change.

In the guide-pooled experiment, we infected cells expressing dCas9–KRAB at high MOI (Methods) and profiled a single cell in each droplet across seven channels, whereas, for the corresponding conventional Perturb-seq, we infected cells with the same guide library at low MOI and analyzed 19 channels. From the guide-pooled experiment, we obtained 24,192 cells after filtering (conventional: 66,283), where 35% of the cells (8,448) contained three or more guides (Fig. 4a), with 2.50 guides on average per cell (conventional: 1.13) (Fig. 4b) and 101 cells containing a guide for each perturbed gene on average (conventional: 115) (Fig. 4c). We measured expression for 16,268 total genes (conventional: 18,617). The guide-pooled screen was approximately three times cheaper than the conventional screen.

### Cell-pooling achieves large efficiency gains

The perturbation effect sizes estimated by Perturb-FR from the cell-pooled Perturb-seq screen (Methods) agreed well with its conventional counterpart. When estimating effects, we included read count, cell cycle and proportion of mitochondrial reads as covariates^2^, and we combined sgRNAs targeting the same gene while retaining the subset of sgRNAs for a gene with maximal concordance of effects across random subsets of the data (Methods). The significant effects from the compressed experiment (*n* = 19,909) were strongly correlated with the corresponding effects from the conventional experiment (Pearson’s *r* = 0.92, sign concordance = 0.96; Fig. 3e). Notably, we observed many more significant effects overall in the conventional screen than the cell-pooled screen (216,220 versus 19,909; FDR q < 0.05), but this is expected given that we intentionally generated a larger and more highly powered conventional screen (144 droplets per perturbation, compared to 90 for the cell-pooled screen) to enable data splitting and cross validation analyses (see below).

**Extended Data Fig. 3:**
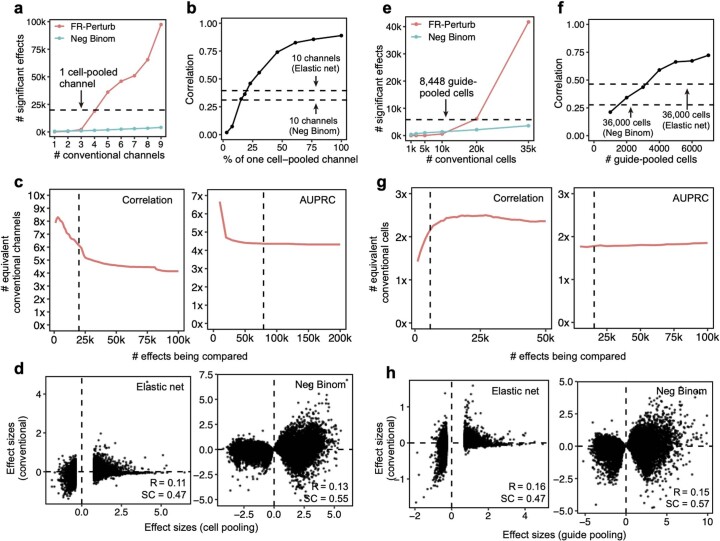
Additional analyses comparing compressed versus conventional screens. (**a**) Number of significant effects (q < 0.05) detected by FR-Perturb and negative binomial regression (y-axis) as a function of number of channels (x-axis) from the conventional knock-out screen. We do not include the number of significant effects from elastic net due to its extremely large magnitude ( > 1,000,000), which is inconsistent with the performance of elastic net in held-out validation analyses. (**b**) Sample size in terms of percentage of a single cell-pooled channel by droplet count (x-axis) versus out-of-sample validation accuracy (y-axis). Validation accuracy of 10 channels analyzed with elastic net or negative binomial regression is indicated with dotted lines. (**c**) Performance of cell-pooled versus conventional screen (y-axis) while varying the number of effects being compared (x-axis). Performance is quantified as the number of conventional channels needed to obtain the same correlation (left) or AUPRC (right) as one cell-pooled channel. Dotted line represents the cutoffs used in Fig. 3e, f. (**d**) Scatterplots of top 19,909 estimated effects from the cell-pooled screen (x-axis) versus the same effects in the conventional screen (y-axis) when estimating effects using elastic net regression (left) or negative binomial regression (right). R = Pearson’s correlation, SC = sign concordance. (**e-h**) Same as **a-d**, but showing results from the guide-pooled screen (restricting to cells with 3 or more guides) and corresponding conventional screen.

**Extended Data Fig. 4:**
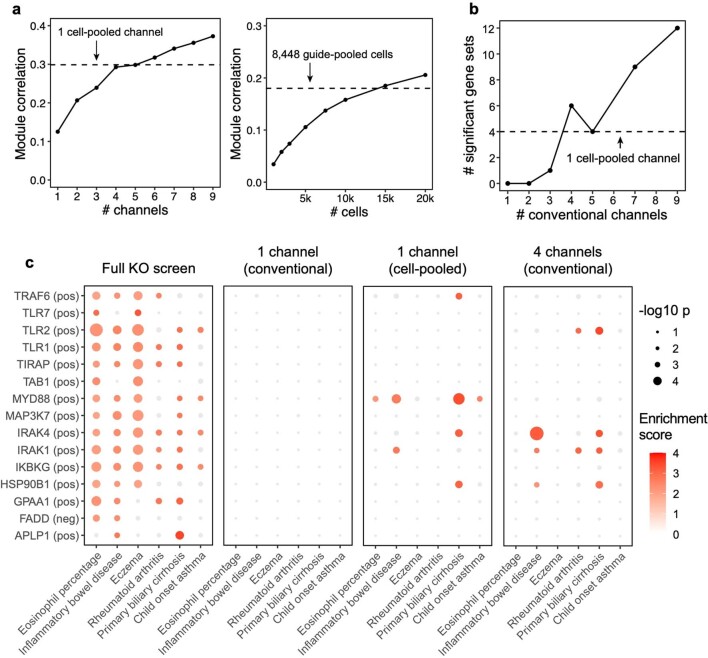
Additional analyses comparing compressed versus conventional screens. (**a**) Same as Fig. 3e (left) and **4e** (right), but correlation (Y-axis) is computed based on perturbation effects on gene modules rather than effects on individual genes. FR-Perturb produces module dictionaries that are correlated but not identical when applied to different datasets, which precludes the direct comparison of perturbation effects on modules in different datasets. Thus, to enable this comparison, the module dictionary was fixed to be the one obtained from the held-out validation dataset for all results above. We note that overall lower correlation is observed in this figure than Figs. 3e and 4e because we compared all perturbation’s effects on all modules rather than only significant effects on genes. (**b**) Same as Fig. 3e, but performance is assessed based on the number of gene sets constructed from the perturbation effects with significant GWAS heritability enrichment estimated using sc-linker (p < 0.001 for at least two traits out of 63 total; same threshold used as Fig. 6a; see Methods and section ‘Integrating Perturb-seq with genome-wide association studies’ in the main text). P-values are two-sided and obtained from sc-linker. (**c**) Individual heritability enrichment estimates for all significant gene sets and traits from the full knock-out screen (combined cell-pooled and conventional screens, leftmost plot). The same effects are shown for gene sets constructed from perturbation effects estimated from 1 conventional channel, 1 cell-pooled channel, and 4 conventional channels. Effects with p > 0.001 are greyed out.

The cell-pooled experiment yielded substantially more signal per experimental unit (channel) than the conventional one (Fig. 3d–f). First, the global clustering of effects learned from a single cell-pooled channel was much less noisy than from a single conventional channel (adjusted Rand index of 0.53 versus 0.31 when comparing clusters with those learned from the full conventional screen; Fig. 3d). Moreover, approximately four conventional channels were needed to obtain the same number of significant effects as one cell-pooled channel (Extended Data Fig. 3a). Next, to quantitatively assess the specificity of each approach, we held out half of the conventional data as a validation set, and then we downsampled the remaining half to different numbers of channels and compared the top 19,909 most significant effects learned from the downsampled data (matching the number of significant effects in the cell-pooled screen) to those in the held-out validation set. We found that 5–6 conventional channels were needed to achieve equivalent validation accuracy (correlation) as one cell-pooled channel (Fig. 3e). The relative efficiency gains of the compressed screen were consistent when varying the number of effects being compared (Extended Data Fig. 3c), when comparing effects on modules rather than on individual genes (Extended Data Fig. 4a) or when evaluating performance based on biological informativeness as reflected by the number of effects with significant heritability enrichment for common diseases (Extended Data Fig. 4b,c). We also assessed the sensitivity of each approach by testing whether the significant effects determined from the validation set were recovered by the downsampled conventional or cell-pooled screens. We constructed precision-recall curves, calling ‘true positives’ the 79,100 significant effects from the validation dataset and varying the classification threshold by the significance of the effects in the downsampled conventional or cell-pooled datasets. One cell-pooled channel had similar area under the precision-recall curve (AUPRC) to four conventional channels (Fig. 3f), with consistent efficiency gains when varying the number of true-positive effects (Extended Data Fig. 3c).

**Extended Data Fig. 5:**
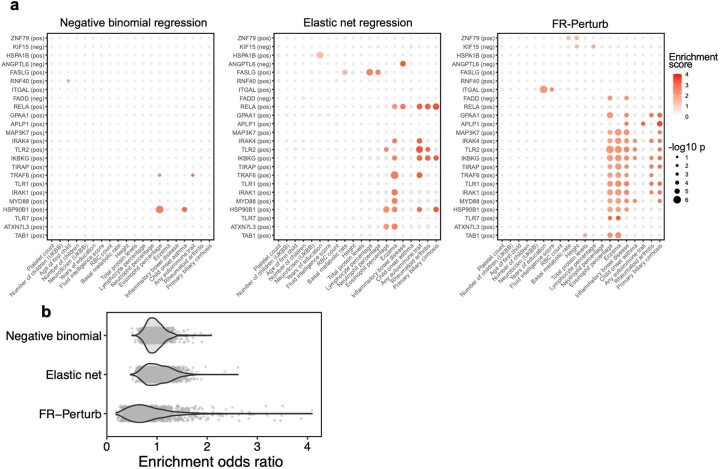
Additional analyses comparing inference methods. **(a**) Heritability enrichment estimates and p-values (estimated using sc-linker; Methods) for gene sets and traits that are significant in at least one of the three inference methods. Gene sets were constructed in the same manner as in Fig. 6a (see section ‘Integrating Perturb-seq with genome-wide association studies’ in the main text). Significance is determined as having two or more effects with p < 0.001 (same threshold used as in Fig. 6a). Greyed out points correspond to p-value > 0.001. Gene sets are constructed from the conventional knock-out screen. (**b**) Odds ratios for enrichment of evolutionarily constrained genes (pLI > 0.9) in all gene sets (comprising the top 500 upregulated or downregulated genes from each perturbation) estimated from the three inference methods. Each point represents a gene set.

Moreover, FR-Perturb substantially outperformed the established inference methods that we tested: elastic net regression^2^ and negative binomial regression^16^. Repeating the same analyses as above with each method (Methods), the concordance between the downsampled conventional data and validation data, and between cell-pooled and conventional data, was much higher with FR-Perturb than previous methods (Fig. 3e,f and Extended Data Fig. 3d). FR-Perturb also identified more biologically informative effects than previous methods, based on the heritability enrichment of common diseases (Extended Data Fig. 5). By downsampling the cell-pooled screen, we found that ~1/5 of a cell-pooled channel analyzed with FR-Perturb achieved the same validation accuracy as 10 conventional channels analyzed with existing methods (Extended Data Fig. 3b). We assessed the cost savings of cell pooling over the conventional approach while factoring in sequencing costs in the Supplementary Note, section 5.

### Guide-pooling achieves large efficiency gains

Guide-pooled Perturb-seq was also concordant with its conventional counterpart, based on a similar evaluation scheme as above. For the guide-pooled screen, we focused on the 8,448 cells with three or more guides. This number of guides per cell can be achieved with sequential transduction, as done for two of the seven channels (Methods and Supplementary Fig. 2). We learned perturbation effects from both screens using FR-Perturb, with slight modifications to account for differences in the guide-pooled versus cell-pooled screens (Methods). The 5,836 significant effects from the guide-pooled cells were strongly correlated with the same effects from the conventional screen (Pearson’s *r* = 0.80, sign concordance = 0.92) (Fig. 4e). Thus, even if some nonlinear effects exist between guides, the overall assumption of additivity holds broadly enough to infer many accurate effects. Analysis of the effects that appear to be visual outliers in the guide-pooled screen (Fig. 4e) showed that they arise from correlated noise rather than genetic interaction effects (Supplementary Note, section 4, and Supplementary Fig. 3). As with the cell-pooled screen, the total number of significant effects was much lower in the 8,448 guide-pooled cells versus the full conventional screen (5,836 versus 95,526; q < 0.05), but this is expected because our conventional screen was, by design, larger and more highly powered overall to enable downsampling analyses.

The guide-pooled screen was substantially more efficient than the conventional screen per experimental unit (cell), and FR-Perturb provided more accurate effect sizes than established methods. Around 2.5× more conventionally studied cells were needed to obtain the same number of significant effects as guide-pooled cells (Extended Data Fig. 3e). Globally, the effect size patterns learned from the same number of cells (8,448 cells) were much less noisy in the guide-pooled screen than in the conventional screen (adjusted Rand index of 0.45 versus 0.35 when comparing clusters with those learned from the full conventional screen; Fig. 4d). Approximately twice as many conventional cells were required to learn effect sizes at the same correlation (Fig. 4e) or to attain the same AUPRC (Fig. 4f) as guide-pooled cells when comparing to a held-out validation set. This relative efficiency gain was consistent when varying the number of compared effects (Extended Data Fig. 3g) or when comparing effects on modules rather than on individual genes (Extended Data Fig. 4a). Moreover, the effect sizes inferred by FR-Perturb had substantially better validation accuracy than those from the two established inference methods in both the guide-pooled and conventional data (Fig. 4e,f and Extended Data Fig. 3h). Around 3,200 guide-pooled cells analyzed with FR-Perturb achieved the same validation accuracy as 36,000 conventional cells analyzed with existing approaches (Fig. 2f), leading to an approximately 10-fold cell count and cost reduction over existing experimental and computational approaches (Supplementary Note, section 5).

### Guide-pooling is the more impactful compression approach

**Extended Data Fig. 6:**
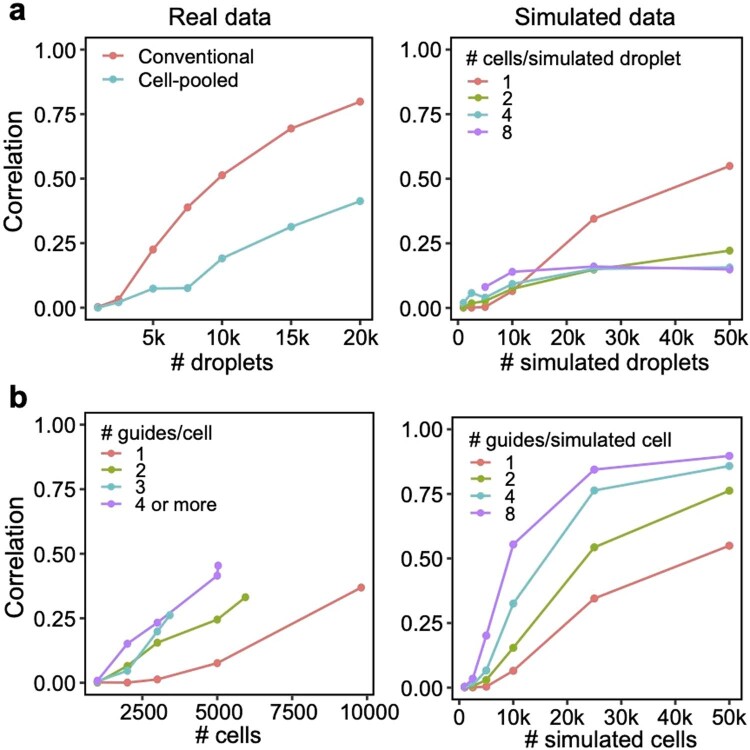
Relationship between degree of overloading and performance. (**a**) Down-sampling droplets from cell-pooled and conventional screens. (Left) Correlation of top 10,000 estimated effects with held-out validation data (y-axis) when varying droplet count (x-axis). (Right) Correlation of top 10,000 estimated effects with true effects in simulations of cell-pooled data with varying numbers of cells/droplet. (**b**) Down-sampling cells from guide-pooled screen stratified by # guides/cell. (Left) Correlation of top 10,000 estimated effects with held-out validation data (y-axis) when varying cell count (x-axis). (Right) Correlation of top 10,000 estimated effects with true effects in simulations of guide-pooled data.

We conducted a detailed comparison of the strengths and limitations of cell-pooling and guide-pooling relative to each other (Supplementary Note, sections 6 and 7, and Supplementary Fig. 4). Notably, the performance of cell-pooling does not scale with the number of cells per droplet, and the overall efficiency gains of cell-pooling stem from obtaining more non-empty droplets per channel (Extended Data Fig. 6). On the other hand, the performance of guide-pooling does scale with the number of guides per cell, with the best performance attained by cells with four or more guides (Extended Data Fig. 6). This suggests that guide-pooling has the potential to achieve even higher efficiency with a greater degree of overloading than we attained in our experiment.

**Extended Data Fig. 7:**
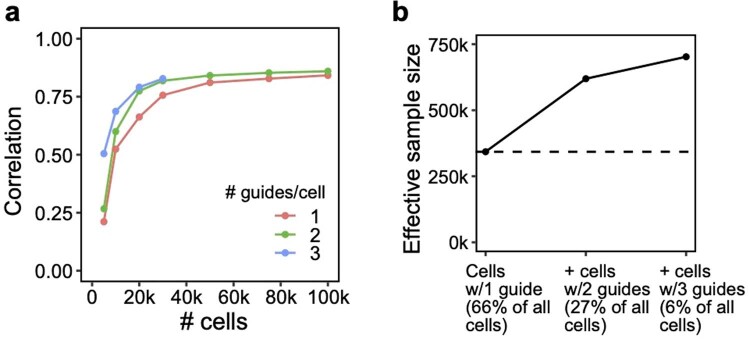
Additional signal in cells containing multiple guides in a conventional 1,130 gene Perturb-seq screen in mouse BMDCs. These cells would normally be discarded before analysis. (**a**) Correlation of top 10,000 estimated effects with held-out validation (y-axis) when varying cell count (x-axis). (**b**) Increase in effective sample size in cells (y-axis) when including cells containing 2 or 3 guides (x-axis). Effective sample size for cells with 2 or 3 guides is computed as the number of single-guide containing cells needed to achieve the same held-out validation accuracy (from **a**).

The effectiveness of compressed Perturb-seq has important implications for existing Perturb-seq screens, each of which already has some overloaded droplets (cell-pooling) and multi-guide-expressing cells (guide-pooling) by chance or by design^1,2,13^. Although these cells/droplets are often discarded, our results suggest that these cells/droplets can contain even more signal than the single-guide/single-cell-containing ones and, thus, should be retained. To illustrate this, we used FR-Perturb to analyze a Perturb-seq knock-out (KO) screen of 1,130 genes in mouse BMDCs^30^. In this screen, 519,535 droplets containing a single cell were obtained, of which 33% contained more than one guide by chance. By stratifying cells by the number of guides and comparing the learned effect sizes from FR-Perturb with a held-out validation subset of the data with single guide perturbations, we show that the accuracy of the effect sizes scales with the number of guides per cell and is highest in cells containing three guides (Extended Data Fig. 7a). Thus, by retaining all cells with more than one guide, the sample size of the experiment could effectively be doubled compared to the conventional approach that discards these cells (Extended Data Fig. 7b).

### Regulatory circuitry of the LPS response

We next leveraged the overall concordance of all perturbation data (conventional and compressed, KO and knock-down (KD)) to investigate the underlying regulatory circuitry of the LPS response. To maximize power, we merged droplets from the compressed and conventional screens together and then re-estimated all effects. There were 251,792 significant effects in the combined conventional and cell-pooled KO screen (131,161 effects in the combined conventional and guide-pooled KD), an increase of 16% (KD: 37%) over the conventional screen alone. We focused all subsequent analyses on effects from these combined screens.

**Fig. 5:**
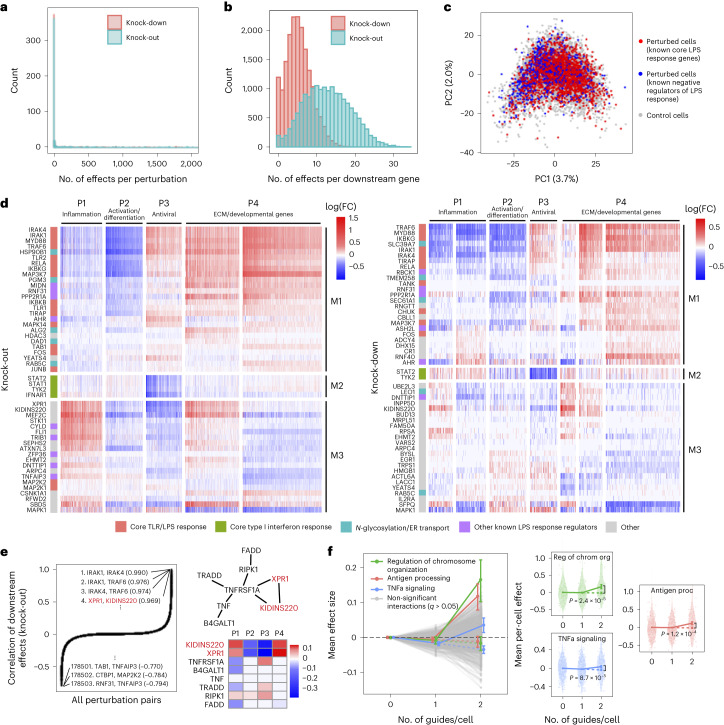
Analysis of KO and KD perturbation effects in the LPS response. **a**, Distribution of perturbed genes based on their number of significant effects (*q* < 0.05) on downstream genes. **b**, Distribution of downstream genes based on how many perturbed genes significantly affect their expression. **c**, PCA of perturbed and control cells based on the expression of the top 2,000 most variable genes. Control cells (gray) contain a non-targeting guide only. Perturbed cells (red/blue) contain a guide for one of the following genes. Red: IKBKB, IKBKG, IRAK1, IRAK4, MAP2K1, MAP3K7, MAPK14, MYD88, RELA, TIRAP, TLR1, TLR2 and TRAF6. Blue: CISH, CYLD, STAT3, TNFAIP3, TRIB1 and ZFP36. Numbers in parentheses indicate percent variance explained by PCs. **d**, Heat maps of perturbation effect sizes (inferred with FR-Perturb) from the KO (left) and KD (right) screens. Rows: top 50 perturbed genes based on the average magnitude of effects on all downstream genes. Columns: top 2,000 downstream genes based on the average magnitude of effects of all perturbed genes acting on them. Rows and columns are clustered using Leiden clustering. Clusters are labeled based on their GO enrichment terms. All effects with *q* > 0.2 are whited out. **e**, Left, correlation of KO effect sizes (*y* axis) between all pairs of perturbed genes (*x* axis). Top and bottom gene pairs are labeled. Top right, graph of all perturbed genes that physically interact with XPR1 and/or KIDINS220, based on AP-MS data from BioPlex 3.0 (ref. ^46^). Edges represent physical interaction. Bottom right, mean effects of perturbed genes from top right on P1–P4. **f**, Analysis of genetic interaction effects. Left, effect sizes relative to control (*y* axis) of cells containing zero, one or two guides (*x* axis) within each perturbation module (lines connecting three dots). Modules with significant effects (*q* < 0.05) are highlighted in color and labeled, with the expected effect of cells containing two guides in the module represented with a dotted line. Error bars represent standard errors obtained from bootstrapping. Right plots, violin plots of the mean effects of individual cells containing zero, one or two guides in the three perturbation modules with significant interaction effects. Dotted line represents the expected effect of cells with two guides. Two-sided *P* values were computed from permutation testing. FC, fold change.

Overall, the KO and KD screens were concordant, with most of the significant effects (FDR *q* < 0.05) attributed to relatively few (~5%) of the perturbations, each with widespread effects on many genes (Fig. 5a). As expected, there were substantially more significant effects in the KO screen compared to the KD screen (251,792 versus 131,161 effects), consistent with larger effects of KO on the target gene’s activity^31^. Effects significant in both screens (*n* = 26,362) were highly correlated between the screens (*r* = 0.92, sign consistency = 0.99; Supplementary Fig. 5a–d). The perturbations did not lead to new global cell states, such that profiles from perturbed (one or more targeting guides) and unperturbed (control guide) cells spanned the same low-dimensional space (Fig. 5c). Thus, although many perturbations had significant and widespread effects, they did not yield radically altered phenotypic states, consistent with previous studies of this cellular response^2^.

We organized the perturbations and genes by clustering their effect size profiles (Methods), observing four broad co-regulated programs of downstream genes with correlated responses across the perturbations and three broad co-functional modules of perturbations with correlated effects on downstream genes (Fig. 5d).

The four major co-regulated programs were present in both the KO and KD screens (Fig. 5d), spanning key aspects of the response to LPS: inflammation (P1: cytokine, chemotaxis and LPS response genes; Supplementary Fig. 5e,f); macrophage differentiation (P2: immune cell activation, differentiation and cell adhesion genes); antiviral response (P3: type I interferon response genes); and extracellular matrix (ECM) and developmental genes (P4) (Supplementary Table 2). Inflammation (P1) and the antiviral response (P3) are known to be regulated by LPS signaling through AP1/NF-κB and IRF3, respectively^32^, and were mostly anti-correlated in their responses to perturbation in our screen, consistent with reports that downregulation of the inflammatory response can lead to upregulation of type I interferon response^33,34^. Inflammatory signaling is known to lead to macrophage differentiation^35^, but almost all perturbations with significant effects on inflammation (P1) (in any direction) downregulated macrophage differentiation (P2). This suggests that additional factors beyond inflammatory signaling mediate macrophage differentiation in response to LPS^36^.

Of the three major co-functional modules, KO/KD of the first module (M1) resulted in strong downregulation of inflammation and macrophage differentiation (P1–P2) and upregulation of the antiviral response and ECM/developmental genes (P3–P4) (Fig. 5d). M1 was mainly composed of core TLR/LPS response genes and genes directly upstream or downstream of the pathway^32^, including MYD88, IRAK1, IRAK4, RELA, TRAF6, TIRAP, IKBKB, IKBKG, TAB1, TANK, TLR1, TLR2, MAPK14, MAP3K7, FOS, JUNB and CHUK. Given the known function of these genes, we expect that their KO/KD will lead to downregulation of inflammation and macrophage differentiation (P1–P2), as we indeed observed. Other genes in M1 previously shown to downregulate TNF and the inflammatory response when knocked out^26^ included two LUBAC complex proteins (RBCK1 and RNF31), genes in the OST complex (DAD1 and TMEM258) and ER transport (HSP90B1, SEC61A1 and ALG2) and other genes with diverse functions (MIDN, AHR, PPP2R1A and ASH2L). M1 also included two additional ER transport genes not previously implicated in immune pathways (RAB5C and PGM3), highlighting the important role of *N*-glycosylation and trafficking in macrophage activation^37^.

KO/KD of the second co-functional module (M2) primarily resulted in strong downregulation of the antiviral program (P3), with weak/mixed effects on other programs. M2 comprised four genes known to be core components of the type I interferon response^38^— STAT1, STAT2, TYK2 and IFNAR1—for which downregulation of the antiviral program in response to their perturbation is expected.

KO/KD of the third and final co-functional module (M3) resulted in upregulation of inflammation (P1), downregulation of macrophage differentiation and the antiviral response (P2–P3) and mixed effects on ECM/development (P4). M3 included many genes with known inhibitory effects on inflammation, including ZFP36, an RNA-binding protein that destabilizes TNF mRNA^39^; enzymes CYLD and TNFAIP3, involved in deubiquitination of NF-κB pathway proteins^40,41^; pseudokinase TRIB1 and ubiquitin ligase RFWD2, which are involved in degradation of JUN^42,43^; and RELA-homolog DNTTIP1 (ref. ^26^). Other genes in M3 included transcription factors (MEF2C, FLI and EGR1), chromatin modifiers (EHMT2 and ATXN7L3) and kinases (CSNK1A1 and STK11).

Interestingly, two of the M3 genes with particularly strong effects on all programs did not have prior immune annotations: XPR1, a retrovirus receptor involved in phosphate export, and KIDINS220, a transmembrane scaffold protein previously reported in neurons^44^. In the KO screen, this pair of genes had the fourth highest correlation of downstream effects (*r* = 0.97) among all ${598}\choose{2}$ = 178,503 perturbation pairs (Fig. 5e), following IRAK1/IRAK4, IRAK1/TRAF6 and IRAK4/TRAF6, which are all known to form a physical LPS signaling complex^32^. XPR1 and KIDINS220 have recently been shown to form a complex that is required for normal regulation phosphate efflux in certain cancer cells^45^. Furthermore, in affinity purification mass spectrometry (AP-MS) data^46^, XPR1 and KIDINS220 physically associate with each other and TNF receptor TNFRSF1A. KO of TNFRSF1A in our screen resulted in effects opposite to XPR1/KIDINS220 KO (Fig. 5e), suggesting a possible inhibitory effect of this complex on TNFRSF1A.

**Extended Data Fig. 8:**
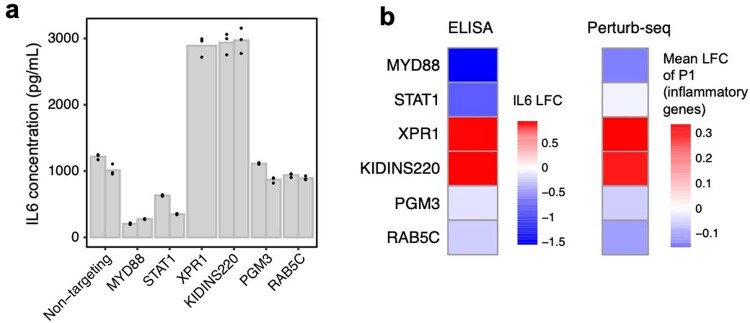
Experimental validation of six regulators of the inflammatory response. RAB5C, PGM3, XPR1, and KIDINS220 represent novel regulators of the inflammatory response, while MYD88 and STAT1 were included as positive controls. (**a**) IL6 concentration (as measured by ELISA) in LPS-stimulated THP1 cells infected with single guides. Two guides were included for each target (excluding XPR1, which only has one guide due to all cells receiving the other guide dying). Individual bars represent guides, while individual points represent experimental replicates. (**b**) Left: Log fold changes of IL6 protein in cells receiving perturbations (averaged across the two guides for each target) relative to non-targeting controls. Right: Mean log fold change of expression of genes in P1 (inflammatory program, see Fig. 5d).

We experimentally validated several of the novel results described in this subsection, namely the effects of RAB5C, PGM3, XPR1 and KIDINS220 KO on the inflammatory response in LPS-stimulated THP1 cells, as measured by the secretion of IL6 (Methods). We found that RAB5C and PGM3 KO both led to a modest decrease (~0.85-fold) in IL6 secretion (consistent with our finding that KO of these genes led to downregulation of the P1 program), whereas XPR1 and KIDINS220 KO both led to a substantial increase (~2.6-fold) in IL6 secretion (consistent with our previous finding that KO of these genes led to upregulation of P1; Extended Data Fig. 8).

### Guide-pooling reveals second-order genetic interactions

Genetic interactions (non-additive effects) between two or more genes can, in principle, be inferred from cells containing two or more guides, which are generated by chance when transducing cells at low or high MOI (Fig. 4b). Here, guide-pooling can provide increased efficiency compared to the conventional approach, as in the first-order case (Supplementary Note, section 9).

We first attempted to estimate second-order interaction effects and their *P* values from the guide-pooled screen and corresponding conventional KD screen by adding interaction terms to the perturbation design matrix (Methods). However, although we could generate point estimates of second-order effects^2^, none of these effects was significant in either screen due to insufficient power (Supplementary Fig. 6a), even with a lax significance threshold (*q* < 0.5).

To increase power, we aggregated perturbations into modules defined by Gene Ontology (GO) annotations (Supplementary Table 3a) and learned the overall impact of second-order interactions within and between each module on each gene program (Methods). Here, we define an interaction effect as the deviation from the sum of first-order effects for cells that contain any two perturbations from either the same module (intra-module interactions) or two different modules (inter-module interactions) (Methods). To ensure adequately sized groupings, we aggregated perturbations into 490 (possibly overlapping) modules each with at least 20 genes, such that any pair of perturbations in each module was represented in an average of 87 cells in the guide-pooled screen (conventional: 30 cells) (Supplementary Fig. 6b). We also constructed 30 non-overlapping modules by clustering the original 490 modules (Methods), resulting in ${30}\choose{2}$ = 435 module pairs, among which we could compute inter-module interactions. To increase power, we grouped downstream genes by their program (P1–P4) membership (Fig. 5d), computing mean effects on these four programs rather than on individual genes. The results from this analysis represent the extent of intra-module and inter-module interactions on each key program.

We detected three co-functional modules with significant (*q* < 0.05) intra-module interaction effects on at least one program from the guide-pooled screen (Fig. 5f and Supplementary Table 3b), whereas we detected no significant interactions from the substantially larger conventional screen (even at *q* < 0.5) (Supplementary Fig. 6c and Supplementary Table 3c). Two of the significant interaction effects—with genes for regulation of chromosome organization (*P* = 2.4 × 10^−5^) and antigen processing (*P* = 1.2 × 10^−4^)—had insignificant first-order effects on the antiviral program (P3) while having significant positive second-order effects. The third, TNFα signaling, had a significant negative first-order effect on the inflammatory/LPS program (P1) (*P* = 2.0 × 10^−4^) and significant positive second-order effect (*P* = 8.7 × 10^−5^). This effect is consistent with the reported nonlinear relationship between gene dosage and TNF signaling activity when comparing heterozygous versus homozygous KO mice for either TNF^47^ or the TNF receptor TNFRSF1A (ref. ^48^). Interestingly, we did not observe any significant inter-module interactions from either screen (Supplementary Fig. 6d and Supplementary Table 3d,e), which may suggest that perturbations in different modules are less likely to interact with each other^49,50^.

### Integrating Perturb-seq with GWASs

Because dysregulation of innate immune responses plays a key role in many human diseases^51^, we next asked whether the perturbation effects learned from our in vitro screens can help identify disease-relevant genes and processes. In vitro screens may be especially helpful for this aim given that many of the perturbed genes from our screens are under strong selective constraint in human populations (Supplementary Fig. 7a), making them challenging to directly connect to disease through GWASs^52^ owing to fewer common variants in or around the gene^53,54^. To investigate this, we obtained summary statistics from GWAS of 64 distinct human diseases and traits (Supplementary Table 4a), including autoimmune diseases and blood traits as well as non-immune traits/diseases (for example, height, body mass index, schizophrenia and type 2 diabetes). Using sc-linker^55^, we computed the overall heritability enrichment of these 64 traits/diseases in single-nucleotide polymorphisms (SNPs) in/around genes comprising perturbation modules M1–M3 (Methods). We observed significant heritability enrichment (*P* < 0.001) for M3 (genes that suppress the LPS response) for two blood traits (lymphocyte and neutrophil percentage), but we did not observe significant enrichment for M1 (positive regulators of the LPS response) or M2 (genes involved in the antiviral response) for any traits (Supplementary Fig. 7b).

Instead, we hypothesized that, if a perturbed gene is important for disease, then disease heritability may be enriched near the downstream genes that it affects^12,56^. To test this hypothesis, we constructed two ‘perturbation signatures’ for each perturbed gene that include all genes that are significantly upregulated (‘negative’ targets) or downregulated (‘positive’ targets) by its KO/KD. We retained signatures with at least 100 genes, resulting in a total of 1,634 perturbation signatures from both the KO and KD screens. We also constructed signatures corresponding to the gene programs P1–P4 (Fig. 5d). As above, we used sc-linker to test for disease heritability enrichment for each signature/phenotype pair (Methods).

**Fig. 6:**
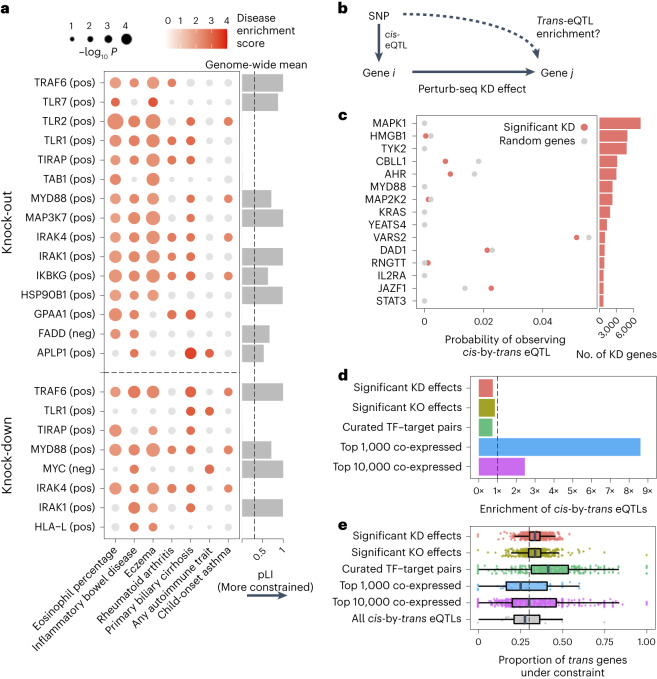
Integration of population genetic screens with Perturb-seq. **a**, Heritability enrichment scores of signatures comprising genes significantly modulated by perturbations (rows) across human traits (columns), computed using sc-linker^55^. ‘pos’ indicates the set of genes whose expression changes in the same direction as the perturbed gene (that is, downregulated by the perturbation), with the opposite applying to ‘neg’. Displayed are all perturbation signatures and traits with at least two significant (*P* < 0.001) effects. Non-significant scores are grayed out. Bar plot: probability of loss-of-function intolerance^54^ (pLI) of the corresponding perturbed gene. **b**, Schematic of eQTL integration analysis, aiming to test whether *trans*-regulatory relationships learned from Perturb-seq are also present in eQTL studies. For all gene pairs in which gene *i* exerts an effect on gene *j* (that is, has a significant KD effect in our Perturb-seq screen), we would expect that gene *i* and gene *j* are enriched for *cis*-by-*trans* eQTLs. **c**, Using data from an eQTL study closely matching our cell type and treatment^27^, shown is the probability of observing significant *cis*-by-*trans* eQTLs among the top 15 perturbed genes from our KD screen and their affected downstream genes (red) compared to random downstream genes (gray). **d**, Enrichment of significant *cis*-by-*trans* eQTLs among various sources of gene–gene pairs: significant KO/KD effects (representing significant gene–gene effects from our KO and KD screens, respectively), curated transcription factor (TF) and target gene pairs^65^ and the top 1,000/10,000 most co-expressed gene pairs (based on correlation of expression across samples) from the eQTL dataset. Enrichment was computed relative to random *trans* genes for each *cis* gene and then averaged over all *cis* genes. **e**, Selective constraint on *trans* genes from **d** plus all significant *cis*-by-*trans* eQTLs from the Fairfax et al.^27^ dataset. Each point represents a *cis* gene, whereas the *x* axis represents the proportion of the *trans* genes for each *cis* gene that are under selective constraint (determined as having a pLI >0.5). Box plots represent the median and first/third quartile of points, whereas the bounds of the whiskers represent 1.5× interquartile range.

Twenty-three signatures associated with 16 perturbed genes had significant heritability enrichment scores for at least two phenotypes (*P* < 0.001). In addition, seven phenotypes that reflect immune or blood traits (IBD, eczema, rheumatoid arthritis, asthma, primary biliary cirrhosis and eosinophil percentage) had significant scores for at least two perturbation signatures (Fig. 6a, Supplementary Fig. 7c,d and Supplementary Table 4b,c). As an important negative control, no non-immune/blood traits had any significant enrichment. Most of the significant signatures (15/23) were from the KO screen, suggesting that the expression effects from KO are more suited for this analysis (either because they are more disease relevant or more powered due to capturing more effects). Among the downstream programs P1–P4, we observed significant enrichment from only P2 on three immune traits: IBD, eczema and primary biliary cirrhosis (Supplementary Fig. 7b).

Most of the significant signatures (17/23) were from genes in core LPS and TLR signaling pathways that fall into perturbation module M1 (even though M1 did not exhibit any direct heritability enrichment itself; Supplementary Fig. 7b): TRAF6 (positive), TLR7 (positive), TLR2 (positive), TLR1 (positive), TIRAP (positive), TAB1 (positive), MYD88 (positive), MAP3K7 (positive), IRAK4 (positive), IRAK1 (positive) and IKBKG (positive). Other significant signatures include HSP90B1 (positive), an ER transport gene important for innate immunity^57^ that is co-functional with the core LPS genes (Fig. 5d); FADD (negative), a pro-apoptotic gene downstream of LPS signaling that serves for negative feedback^32^; MYC (negative), an oncogene with known immunosuppressive effects^58,59^; and poorly characterized pseudogene HLA-L. The two remaining significant signatures are for genes whose functions are not previously associated with the immune system, including APLP1 (an amyloid beta precursor-like gene primarily involved in brain function that, interestingly, contains a missense variant associated with severe influenza^60^) and GPAA1 (involved in anchoring proteins to the cell membrane). Thus, by leveraging gene–gene links learned from our screens, we were able to identify disease-relevant genes that we were underpowered to detect through direct heritability analyses (Discussion).

To complement our results that focus on common diseases and variants, we also computed the enrichment of Mendelian immune disease genes among the same signatures derived from our screens from above. We found significant enrichment in a similar number of signatures, particularly those with strong effects on the antiviral response (Supplementary Note, section 10, and Supplementary Fig. 8).

### Perturbation effects do not concord with trans-eQTLs

*Trans*-genetic gene regulation (that is, regulation of gene expression distal to the given SNP) has been proposed as a primary mediator of genetic effects on human disease^61^. *Trans*-genetic gene regulation can be studied through either population-level genetic data (via eQTL studies^62,63^) or experimental perturbation of gene expression^12^, such as the screens conducted in our study. Although both types of data can, in principle, be used to learn the same *trans* effects, their consistency with each other has not been empirically evaluated.

We, therefore, compared gene–gene regulatory links between our Perturb-seq screen and a *trans*-eQTL analysis in primary patient-derived monocytes treated with LPS^27^ (*n* = 432), closely matching our cell line. For validation, we repeated this analysis using a much larger *trans*-eQTL dataset (eQTLGen; *n* = 31,684) although in a model system less similar to ours (whole blood samples). We define a gene–gene regulatory link in eQTL studies based on *cis*-by-*trans* co-localization, where a *cis*-eQTL for gene *i* is also a *trans*-eQTL for gene *j* via a (presumed) *trans*-regulatory effect of gene *i* on gene *j* (Fig. 6b). Here, we assume that a perturbation of a *cis*-eQTL on the expression of gene *i* is analogous to the experimental KD in our system. We used coloc^64^ to compute the posterior probability of *cis*-by-*trans* co-localization while accounting for linkage disequilibrium (LD) between SNPs (Methods). To determine whether the regulatory links learned for a given perturbed gene *i* from Perturb-seq are reflected in the eQTL analysis, we compared the proportion of downstream genes *j* of gene *i* in Perturb-seq that co-localize with gene *i* in the eQTL study, $P\left({colo}{c}_{{gene\; i}\to {gene\; j}}\right)$, with the proportion of random expressed genes that co-localize with *i*, $P\left({colo}{c}_{{gene\; i}\to {random\; gene}}\right)$ (Methods).

Surprisingly, $P\left({colo}{c}_{{gene\; i}\to {gene\; j}}\right)$ was slightly lower than $P\left({colo}{c}_{{gene\; i}\to {random\; gene}}\right)$ for individual perturbed genes *i* (Fig. 6c and Supplementary Table 5) as well as when aggregating across all perturbed genes (Fig. 6d). Moreover, we observed no relationship between either the significance or magnitude of the effect of gene *i* on gene *j* and $P\left({colo}{c}_{{gene\; i}\to {gene\; j}}\right)$ (Supplementary Fig. 9a). We observed similar negative results when obtaining gene–gene links from our KO data or from a curated list of transcription factor–target gene pairs^65^ (Fig. 6d). Using an alternative way of quantifying gene–gene links in eQTL studies that does not make assumptions about the number of causal variants (that is, bivariate Haseman–Elston regression to estimate genetic correlation of expression^66^; Methods) yielded similar results (Supplementary Fig. 9b,c). We observed similar negative results when taking *cis*-by-*trans* eQTLs from eQTLGen (Supplementary Fig. 10).

Conversely, we did observe significant enrichment of *cis*-by-*trans* eQTLs in gene pairs co-expressed in the same eQTL study (Fig. 6d), as has been observed in other *trans*-eQTL studies^62^. Notably, co-expression in eQTL datasets is dominated by environmental effects rather than genetic effects^67^. Thus, given that the two effects are independent across samples, we would not ordinarily expect the most strongly co-expressed genes to be enriched for *cis*-by-*trans* eQTLs, suggesting that they may be confounded, in part, by unmodeled technical artifacts or inter-cellular heterogeneity (Supplementary Note, section 11). We also observed that the level of negative selection on the *trans* gene mirrored the patterns of *cis*-by-*trans* eQTL enrichment (or lack thereof) that we observed in the previous analyses (Fig. 6e), suggesting that our power to detect *cis*-by-*trans* eQTLs was affected by selection-induced depletion of SNPs affecting the *trans* genes^54,68^ (Supplementary Note, section 12).

## Discussion

In the present study, we evaluated a new approach for conducting Perturb-seq based on generating composite samples, which involves either overloading microfluidics chips to generate droplets containing multiple cells (cell-pooling) or infecting cells at high MOI so that each cell contains multiple guides (guide-pooling). We also propose a new method, FR-Perturb, to estimate perturbation effect sizes from composite samples, which increases power by estimating sparsity-constrained effects on latent gene expression factors rather than on individual genes. We tested our approach by perturbing 598 immune-related genes in a human macrophage cell line. We found that our experimental approaches of cell-pooling and guide-pooling, combined with the use of FR-Perturb to infer effect sizes, led to substantial cost reductions over conventional Perturb-seq while maintaining the same accuracy. Guide-pooling also substantially increases power to detect genetic interaction effects and reduces the number of cells needed for screening.

Here we report that cell-pooling led to a 4–20-fold cost reduction, and guide-pooling led to a 10-fold cost reduction, over existing approaches (Supplementary Note, section 5). Both these approaches reduce costs due to RNA library preparation without altering the sequencing step of scRNA-seq. Thus, they can, in principle, be paired with approaches that increase the efficiency of sequencing via new technologies^69^ or targeted sequencing^70^, resulting in further improvements to the efficiency of Perturb-seq. Concurrent results also demonstrate the power of compressed screening with bio-chemical perturbations in high-fidelity cellular model systems (Mead et al.^71^, companion manuscript).

Inference with FR-Perturb leads to substantially improved out-of-sample validation accuracy over conventional gene-by-gene methods (for example, elastic net and negative binomial regression) in both conventionally generated data and compressed data. FR-Perturb is, thus, useful for inferring effects in any type of Perturb-seq screen, even conventional screens that do not adopt our proposed experimental changes. The improved performance of FR-Perturb in both conventional and compressed settings likely stems from perturbation effect sizes being inferred on latent gene expression factors that aggregate many co-expressed genes, thereby denoising the expression counts of individual genes that are especially noisy/sparse in single-cell data. However, the performance of FR-Perturb is likely to suffer when inferring effects for perturbations that cannot be well approximated by these factors (due to idiosyncratic effects of the perturbations^21^).

**Extended Data Fig. 9:**
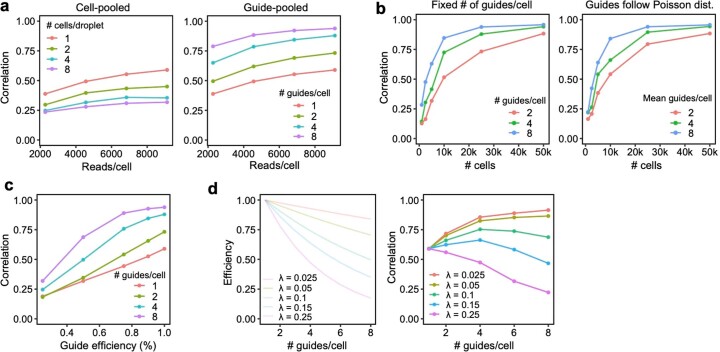
Additional simulations. (**a**) Performance of cell/guide pooling when varying sequencing depth (X-axis). Y-axis: correlation of the top 10,000 most significant effects with the true effects. (**b**) Performance of guide pooling when simulating cells with a fixed number of guides per cell (left; matching the simulation in Extended Data Fig. 6) or when simulating cells with number of guides following a zero-truncated Poisson distribution with mean guides/cell matching the left plot. (**c**) Performance of guide pooling vs. the efficiency of all guides (x-axis). Guide efficiency is simulated as the proportion of guides that had the intended effect on their target. For example, for a guide efficiency of 0.8, 20% of guides were randomly selected to have no downstream effects. (**d**) Performance of guide pooling when efficiency within cells decays as a function of the number of guides per cell. Left: 5 different simulated decay scenarios, where the efficiency per cell = *e*^−*λ*(*x*−1)^ and *x* is the number of guides in the cell. Right: Performance of guide pooling across different # of guides/cell for these 5 scenarios.

Cell-pooling and guide-pooling are complementary approaches with different strengths and limitations. Unlike cell-pooling, guide-pooling has the drawbacks that it requires that nonlinear interaction effects do not systematically bias phenotypes (although not all interaction effects will impart bias; Supplementary Note, section 2), and it potentially suffers from cellular toxicity caused by multiple viruses infecting each cell and/or multiple double-stranded breaks. In addition, unlike guide-pooling, cell-pooling has the drawbacks that it requires increased sequencing depth per channel to account for more non-empty droplets, and it loses per-droplet signal due to dilution of effect sizes (Supplementary Note, section 8). Due to the latter fact, cell-pooling requires many more cells than guide-pooling to achieve the same performance, which can be prohibitive in certain settings where cell count is limited^8,13^. Because guide-pooling performs best with high guide number per cell (four or more), whereas cell-pooling does not perform well with high cell count per droplet, we posit that guide-pooling (but not cell-pooling) can be readily scaled up to very compressed designs (in which case the use of KD over KO and Cas12/13 over Cas9 may be desirable to avoid cellular toxicity), likely leading to even larger efficiency gains than we observed in our screens. To aid in the design of future experiments, we also conducted simulations showing the performance of compressed Perturb-seq when varying factors such as sequencing depth and guide efficiency, finding that is it is robust in many different scenarios (Supplementary Note, section 13, Extended Data Fig. 9 and Supplementary Fig. 11).

**Extended Data Fig. 10:**
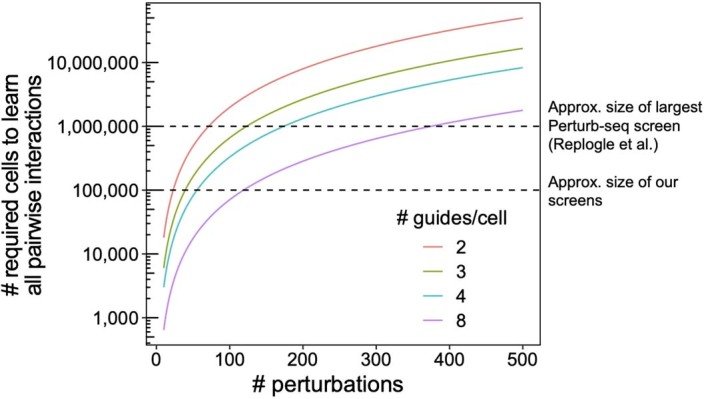
Theoretical number of cells needed to learn pairwise interactions at different levels of guide pooling. Number of total perturbations (x-axis) vs. number of cells needed to learn second-order interaction effects between all pairs of perturbation (y-axis), based on the formula N = 400 * C(p, 2) / C(k, 2), where N is the number of cells, p is the number of perturbations, and k the number of guides per cell.

An additional key advantage of guide-pooling over cell-pooling is that guide-pooling naturally allows for the study of higher-order interaction effects. In our study, we were underpowered (even with guide-pooling) to detect second-order interaction effects between individual gene pairs. However, we detected significant intra-module interaction effects from the guide-pooled but not conventional screen, serving as a proof of concept that such signal can be detected in the guide-pooled screen and may be further probed in more powered future experiments. The efficiency gains brought about from guide-pooling can, in theory, counteract the exponential growth of gene combinations (given that various assumptions are satisfied), potentially making it the only tractable way to systematically study higher-order interaction effects (Supplementary Note, section 9). To aid in the design of future experiments, we conducted simulations showing the number of cells needed to learn second-order interaction effects at various levels of guide-pooling, finding that guide-pooling can markedly reduce the number of cells needed to learn a given number of second-order interaction effects (Supplementary Note, section 14, and Extended Data Fig. 10).

By integrating data from GWASs, our screens highlighted perturbed genes with downstream genes enriched for disease heritability. Many of these perturbed genes are under strong selective constraint and would require up to millions of samples to detect in GWAS^72^. Thus, our analysis represents a potential way to circumvent the issue of negative selection removing GWAS signal from some large-effect disease-relevant genes, a key challenge for biological interpretation of common-variant GWAS.

Gene–gene effects learned from our Perturb-seq screens were not enriched for *cis*-by-*trans* eQTLs in a closely matched cell type and treatment. Many possible explanations exist for this observation, including (1) insufficient power to detect *trans*-eQTLs in the eQTL dataset; (2) biological differences between our cell line and primary monocytes used in the eQTL study; (3) large differences in the magnitude of perturbation between experimental KO/KD and eQTLs; and (4) confounders in the eQTL dataset (Supplementary Note, section 11). Explanation (1) can, in theory, be addressed with larger *trans*-eQTL studies^62^, although we observed similar negative results when replicating our results in a large *trans*-eQTL dataset (eQTLGen). Such studies often suffer from issues with confounding/intercellular heterogeneity, as evidenced by very low reported out-of-sample replication accuracy and substantial overlap (>50%) of detected *trans*-eQTLs with variants known to influence cell type proportion^62^. In addition, single-cell eQTL studies^73^ can potentially address explanation (4), although such studies suffer from low power relative to sample size (~1,000 significant *trans*-eQTL effects detected from ~1.2 million cells^73^ versus ~200,000 *trans* perturbation effects detected from ~100,000 cells in our screen). We propose that our compressed screen is a powerful tool to learn *trans* effects on gene expression, although additional work is needed to fully reconcile the differences between population-level genetic screens and experimental perturbation screens.

## Methods

### Experimental procedures

#### Cell culture and stimulation

THP1 cells (American Type Culture Collection (ATCC), TIB202) were cultured in RPMI medium (ATCC, 30-2001) supplemented with 10% FBS (ATCC, 30-2020) and 0.05 mM 2-mercaptoethanol (Sigma-Aldrich, M7522). Cells were maintained between 0.8 and 2 million cells per milliliter.

Cell lines for KO and KD screens were engineered with lentiviral vectors containing Cas9 (pxpr311) and dCas9–KRAB (pxpr121), respectively. Viruses were prepared using a previously published protocol (https://portals.broadinstitute.org/gpp/public/dir/download?dirpath=protocols/production&filename=TRC%20shRNA%20sgRNA%20ORF%20Low%20Throughput%20Viral%20Production%20201506.pdf) and concentrated by centrifugation in a column with a cut size of 100 kDa (MilliporeSigma, UFC903096). Cells were transduced by spinfection as previously described (https://portals.broadinstitute.org/gpp/public/resources/protocols).

THP1 cell lines were infected with sgRNA libraries (described below) at an MOI specific for each guide-pooled experiment. Twelve hours after spinfection, cells and media were diluted 1:10, and cells were allowed to recover for 48 h. Cells were selected with puromycin (2 μg ml^−1^) for 4 d. The selected cells were differentiated into macrophages by stimulation in 20 ng ml^−1^ phorbol 12-myristate 13-acetate (Sigma-Aldrich, P8139-1mg) for 24 h. Cells were then allowed to rest in normal culture medium for 48 h before stimulation in medium containing 100 ng ml^−1^ LPS (MilliporeSigma, L4391-1mg) for 3 h.

#### Guide library production and validation

sgRNAs for the perturbed panel of genes (described below) were designed using the CRISPR-Pick tool from the Broad Institute. Four distinct sgRNAs were designed for each perturbed gene. In addition, 500 non-targeting sgRNAs and 500 safe-targeting sgRNAs (that is, guides targeting intergenic regions of the genome) were included. Oligonucleotide libraries were synthesized by Twist Biosciences and then amplified and inserted into a CROP-seq vector^4^ with sgOpti scaffold (Addgene, 106280) via Gibson assembly. Cloned libraries for KO, KD and control sgRNAs (non-targeting and safe-targeting) were sequence validated as previously described (https://portals.broadinstitute.org/gpp/public/dir/download?dirpath=protocols/production&filename=cloning_of_oligos_for_sgRNA_shRNA_nov2019.pdf). Viral libraries were produced as described above (without concentration), and an MOI was determined by transfecting cells with scaled dilutions of the virus covering a 100-fold dynamic range and quantifying survival rate after selection.

#### Conventional Perturb-Seq, cell-pooling and guide-pooling (scRNA-seq and dialout library production)

For conventional screens, the infected (MOI 0.25) and stimulated THP1 cell suspension was prepared for droplet generation according to the manufacturer’s suggested protocol (10x Genomics, CG00053 Rev C). Channels aiming to recover 5,000–10,000 cells were loaded on the 10x Chromium Controller, and the protocol was followed according to the manual for Chromium Next GEM Single Cell 3′ Reagent Kits version 3.1 (CG000315 Rev C).

For cell-pooling (MOI 0.25), the standard 10x Genomics single-cell 3′ RNA-seq protocol (Chromium Next GEM Single Cell 3′ GEM, Library & Gel Bead Kit version 3.1, PN-1000121) was run according to the manufacturer’s recommendations, except that the concentration of cells was increased to co-encapsulate multiple cells per droplet (250,000 cells loaded per channel).

For guide-pooling, cells were infected at an MOI of 10 before selection and stimulation or were left to rest for 2 d after initial infection before infecting a second time at an MOI of 10 before selection and stimulation (Supplementary Fig. 2). High MOI cells were loaded into droplets as in the conventional screens.

After the generation of double-stranded cDNA, part of the whole transcriptome amplification (WTA) product was set aside for targeted amplification to recover the perturbation barcode. Then, 10 ng of WTA from each channel was input into eight cycles of PCR (primer 1 CTACACGACGCTCTTCCGATCT; primer 2 GTGACTGGAGTTCAGACGTGTGCTCTTCCGATCTTGTGGAAAGGACGAAACACC). The sample underwent a 1× AMPure XP Reagent SPRI clean (Beckman Coulter, A63881) and was amplified for another nine cycles with 8 bp indexed PCR primers and purified with a 0.7× SPRI clean (primer 1 AATGATACGGCGACCACCGAGATCTACACTCTTTCCCTACACGACGCTC, primer 2 CAAGCAGAAGACGGCATACGAGATGTCGAGCAGTGACTGGAGTTCAGACGTGTGCTCTTCCGATCT).

#### Guide effect validation screens

For guide effect validation, two guides (out of four) were chosen for six targets—MYD88, STAT1, RAB5C, PGM3, XPR1 and KIDINS220—as well as two of the non-targeting controls. RAB5C, PGM3, XPR1 and KIDINS220 represent novel regulators of the inflammatory response, and MYD88 and STAT1 were included as positive controls. The two guides for each target were selected by computing the pairwise correlation of effect sizes of the four individual guides on all genes and then taking the pair with the highest correlation. Single guides were cloned into the CROP-seq vector as previously detailed. Two million cells were infected for each guide. Cells were then selected with 4 µg ml^−1^ puromycin for 2 d and then expanded in culture for 10 d. Cells infected with the first guide targeting XPR1 all died, so that condition was removed from the validation experiment. THP1 cells were differentiated into macrophages using PMA as in the main screen. Three wells of a 24-well plate were seeded for each guide, with 250,000 cells per well. After 24 h in PMA, the medium was changed for fresh medium, and cells recovered for 2 d. Cells were then stimulated with 250 µl of medium containing LPS (100 ng ml^−1^) for 8 h, and then medium was collected, spun at 1,000*g* for 2 min to remove cell debris and stored at −80 °C. Two extra wells of cells infected with non-targeting guides received fresh medium as a non-stimulated control. ELISAs were conducted following the manufacturerʼs protocol (https://www.abcam.com/ps/products/178/ab178013/documents/Human-IL-6-ELISA-kit-protocol-book-v4a-ab178013%20(website).pdf).

### Computational procedures

#### Selecting genes to be perturbed

A set of perturbed genes was compiled from several sources (Supplementary Table 1). These included a manually curated list of 35 canonical LPS response genes; the top 100 genes from a previous genome-wide CRISPR screen for regulation of TNF expression after LPS stimulation^26^; 100 genes identified as being a *cis-*eQTL target of SNPs that were (in total) associated with *trans*-eQTL effects for at least four downstream genes in primary monocytes treated with LPS^27^; 95 genes near high-confidence variants in IBD GWAS loci^74^; 108 genes associated with Mendelian disorders identified by search for ‘bacterial infection’ in the OMIM database^75^ and 115 Mendelian genes similarly identified by ‘NF-κB’ search; and 173 genes reported in studies identified by a GWAS Catalog^76^ search for ‘infection’ with diseases/traits related to liver disease and HIV-1 infection excluded.

The (perhaps surprisingly small) intersections between gene lists from these sources are depicted in Supplementary Fig. 1. The final list of 598 perturbed genes was obtained by intersecting genes expressed in THP1 cells with the combined list of 758 genes from all sources.

#### Generating expression and perturbation design matrix

Starting with raw Illumina BCL files from the sequencing output, the ‘cellranger mkfastq’ command with default parameters (from the 10x Cell Ranger tool version 6.0.1; https://support.10xgenomics.com/single-cell-gene-expression/software/downloads/latest) was used to generate FASTQ files. The ‘cellranger count’ command with default parameters was used to align the expression reads to the GRCh38 build of the human transcriptome and generate a gene expression count matrix (see below for details on normalization of expression counts).

To generate the droplet by perturbation design matrix, paired-end reads (in FASTQ format) containing a droplet barcode and unique molecular identifier (UMI) on read 1 and sgRNA sequence on read 2 were aligned using Bowtie2 as follows. Read 2 reads were aligned to a reference constructed from the labeled sgRNA sequences using the –local option with default parameters, which performs local read alignment. Then, using a custom script, droplet barcodes were matched to the mapped guides for each paired-end read. A guide was called as ‘present’ in a droplet if there were at least five UMIs for each droplet barcode–guide barcode pair.

#### Inference using FR-Perturb

From the sequencing output of each of our Perturb-seq experiments, two matrices were directly generated (see above):

- *N* × *G* raw gene expression count matrix ***Y***, where *N* is the number of droplets and *G* is the number of sequenced genes.
- *N* × *P* perturbation design matrix ***X***, where *N* is the number of droplets and *P* is the total number of perturbed genes. Here, *x*_*ij*_ represents a binary indicator variable for whether droplet *i* contains a guide targeting gene *j* (we discuss below how we collapse multiple guides for the same gene). ***X*** also includes two additional columns corresponding to the presence of a non-targeting control guide and a safe-targeting guide, respectively. Cells containing a non-targeting guide are treated as ‘control’ cells (see below), whereas cells containing a safe-targeting guide are used to test for general effects of genome-targeting guides.

From these data, a *P* × *G* effect size matrix ***B*** is estimated, where *β*_*ij*_ represents the log fold change of the expression of gene *j* relative to control expression when gene *i* is perturbed. Two slightly different versions of FR-Perturb were formulated to learn ***B*** from ***X*** and ***Y*** generated from cell-pooling and guide-pooling, respectively, as follows.

##### Version 1: composition in expression space (for cell-pooling)

This scenario arises from cell-pooling. The relationship among ***B***, ***X*** and ***Y*** in a given droplet *i* is modeled as:

1 $$\begin{array}{c}E\left[{{\boldsymbol{y}}}_{{\boldsymbol{i}}}\right]=\frac{1}{{g}_{i}}\mathop{\sum }\limits_{j}^{P}{x}_{{ij}}{\boldsymbol{c}}\exp {\big(\,{\boldsymbol{\beta }}}_{{\boldsymbol{j}}}\big),\end{array}$$

where ***y***_***i***_ is a vector of length *G* corresponding to the expression counts of all genes in droplet *i*; *g*_*i*_ is the number of guides contained in droplet *i* (used as a proxy for the number of cells in the droplet); *x*_*ij*_ is a binary scalar indicating whether cell *i* contains a guide for gene *j*; ***c*** is a vector of length *G* indicating the expected control expression counts of all genes; and $\exp {\big(\,{\boldsymbol{\beta }}}_{{\boldsymbol{j}}}\big)$ is a vector of length *G* indicating the fold change of expression relative to control expression for cells containing a guide for gene *j* (with ***β***_***j***_ representing the log fold change). Note that the ‘exp’ symbol here is used to distinguish fold changes from log fold changes, because the latter units are more commonly used to report effect sizes on gene expression. Conceptually, this model reflects the fact that expected expression measured in a droplet containing *g*_*i*_ cells is the average of the expected expression counts of the individual cells in the droplet (where the latter quantity can be expressed as ${\boldsymbol{c}}\exp ({{\boldsymbol{\beta }}}_{{\boldsymbol{j}}})$ for cells containing guide *j*).

In practice, it is advantageous to model the measured expression in each droplet as the geometric rather than arithmetic mean of expression of the constituent cells. Simulations with real cells show that the arithmetic versus geometric means of expression across multiple cells are very similar (Supplementary Fig. 12a), but modeling expression counts in a droplet as the latter enables us to perform inference in the space of log fold changes rather than fold changes. The former is symmetric around zero (whereas the latter is not) and, thus, leads to balanced inference of upregulation versus downregulation.

Thus, equation (1) is rewritten as follows:

$$E\left[{{\boldsymbol{y}}}_{{\boldsymbol{i}}}\right]={\left(\mathop{\prod }\limits_{j}^{P}{\boldsymbol{c}}\,\exp {(\,{\boldsymbol{\beta }}_{{\boldsymbol{j}}})}^{{x}_{{ij}}}\right)}^{\frac{1}{{g}_{i}}}$$

2 $$\begin{array}{c}E\left[\log \left({{\boldsymbol{y}}}_{{\boldsymbol{i}}}\right)\right]=\log \left({\boldsymbol{c}}\right)+\frac{1}{{g}_{i}}\mathop{\sum }\limits_{j}^{P}{x}_{{ij}}{{\boldsymbol{\beta }}}_{{\boldsymbol{j}}}\end{array}$$

Equation (2) can be expressed simply in matrix form as $E\left[{{\boldsymbol{Y}}^{\prime}}\right]={{{\boldsymbol{X}}^{\prime}}B}$, where each row of ***Y*′,** ${{\boldsymbol{y}}}_{{\boldsymbol{i}}}^{{\boldsymbol{{\prime} }}}$, equals $\log \left({{\boldsymbol{y}}}_{{\boldsymbol{i}}}\right)-\log \left({\boldsymbol{c}}\right)$, and ***X*′** is ***X*** with rows normalized to sum 1. To infer ***B***, ***Y*** is transformed into ***Y*′** by taking the log(TP10K + 1) of all gene expression counts and subtracting $\log ({\boldsymbol{c}})$ from each row of *Y* (where $\log ({\boldsymbol{c}})$ represents the average log(TP10K + 1) of all genes in cells containing only non-targeting control guides). A pseudocount of 1 is included because the sparse nature of gene expression counts prevents directly taking their logarithm.

Next, the factorize-recover algorithm is applied to ***Y*′** and ***X*′** to infer ***B***. In the first ‘factorize’ step of factorize-recover, sparse factorization is applied to ***Y*′** alone using sparse PCA, which produces *N* × *R* left factor matrix $\widetilde{{\boldsymbol{U}}}$ and *R* × *G* right factor matrix ***W***. *R* is a hyperparameter that controls the rank of ***Y*′**. In the second ‘recover’ step, sparse recovery is used to learn *P* × *R* matrix ***U*** from the following regression model: $\widetilde{{\boldsymbol{U}}}={\boldsymbol{X\text{'}U}}$, using LASSO applied to each column of $\widetilde{{\boldsymbol{U}}}$ (so that one column of ***U*** is learned at a time). By multiplying ***U*** by ***W*** obtained from the factorize step, a *P* × *G* matrix $\hat{{\boldsymbol{B}}}$ is obtained, which is an estimate of ***B***.

In practice, the magnitude of elements of $\widehat{{\boldsymbol{B}}}$ was strongly correlated with the overall expression level of the downstream gene in control cells. This correlation changed (but was not removed) when varying the arbitrary pseudocount of 1 and/or scale factor of 10,000, suggesting that it was an artifact arising from log-transforming lowly expressed gene expression counts^77^. Indeed, simulations show that the magnitude of effects estimated with FR-Perturb had a negative bias that scaled with the expression level of the downstream gene, with the largest biases observed for the most lowly expressed genes (Supplementary Fig. 12c).

This bias was removed with the following heuristic correction. First, LOESS was used to fit a curve to the plot of effect size magnitude versus expression level in control cells for all entries of $\hat{{\boldsymbol{B}}}$. Next, all effect sizes were scaled based on the ratio of their fitted effect size magnitude from LOESS and the fitted effect size magnitude of genes with the highest expression counts (log(average TP10K) > 2). This procedure removes the global relationship between effect size magnitude and expression level of the downstream gene while preserving heterogeneity in the average magnitude of effect sizes on individual downstream genes. In simulations, this procedure produced much less biased effect size estimates than when not scaling (Supplementary Fig. 12b,c).

##### Version 2: composition in log fold change effect size space (for guide-pooling)

For guide-pooling data, the relationship among ***B***, ***X*** and ***Y*** in a given droplet *i* is modeled as:

3 $$\begin{array}{c}E\left[\log \left({{\boldsymbol{y}}}_{{\boldsymbol{i}}}\right)\right]=\log \left({\boldsymbol{c}}\right)+\mathop{\sum }\limits_{j}^{P}{x}_{{ij}}{{\boldsymbol{\beta }}}_{{\boldsymbol{j}}}\end{array}$$

The only difference between equation (2) and equation (3) is the absence of the normalizing factor $\frac{1}{{g}_{i}}$ in front of the second term of the right side of equation (3). Inference to learn ***B*** is performed as in version 1, with the only difference being that the rows of ***X*** are not normalized to have a sum of 1.

#### Covariates

Covariates corresponding to the proportion of mitochondrial reads, the total read count per cell and cell cycle state (as determined by the CellCycleScoring function from the Seurat R package^78^) were accounted for when estimating effect sizes using FR-Perturb, by regressing the covariates out of the expression matrix according to the linear model ${\boldsymbol{Y}}^{\,{\prime}}{\boldsymbol{=}}{\boldsymbol{CD}}$. Here, ***Y*′** represents the *N* × *G* normalized expression matrix (where *N* is the number of cells and *G* is the number of sequenced genes); ***C*** represents the *N* × (*C* + 1) covariate matrix including an intercept term (where *C* represents number of covariates with all covariates centered to mean 0); and ***D*** represents the fitted (*C* + 1) × *G* matrix of covariate effects on gene expression. All downstream inference was performed on the residual matrix ${{\boldsymbol{Y}}}_{{resid}}{\boldsymbol{=}}{{\boldsymbol{Y}}^{\prime}}{\boldsymbol{-}}{\boldsymbol{CD}}$.

#### Hyperparameters for FR-Perturb

The spams R package^79^ was used to perform the steps of factorize-recover, including sparse PCA and LASSO. Three hyperparameters are set in FR-Perturb: the rank *R* of ***Y*′**, a tuning parameter *λ*_1_ for sparse PCA during the factorize step (which is the solution of $\mathop{\min }\nolimits_{{\boldsymbol{W}}}\frac{1}{n}{\sum }_{i=1}^{n}\mathop{\min }\nolimits_{{\widetilde{{\boldsymbol{u}}}}_{i}}{{||}{{\boldsymbol{y}}}_{i}-{\boldsymbol{W}}{\,\widetilde{{\boldsymbol{u}}}}_{i}{||}}_{2}^{2}$ so that ${{||}{\widetilde{{\boldsymbol{u}}}}_{i}{||}}_{1}\le {\lambda }_{1}$), and a tuning parameter *λ*_2_ for LASSO during the recover step (which is the solution of $\mathop{\min }\nolimits_{{\boldsymbol{u}}}\,{{||}\widetilde{{\boldsymbol{u}}}-{\boldsymbol{Xu}}{||}}_{2}^{2}$ so that ${{||}{\boldsymbol{u}}{||}}_{1}\le {\lambda }_{2}$). These were set based on maximizing cross-validation *r*^2^ as *R* = 10, *λ*_1_ = 0.1 and *λ*_2_ = 10. Analysis results were not especially sensitive to different values of *R*, *λ*_1_ and *λ*_2_ (Supplementary Fig. 12d–f).

#### Permutation testing for significance

Permutation testing was used to obtain two-tailed *P* values for elements of $\hat{{\boldsymbol{B}}}$. To generate an empirical null distribution for each element of $\hat{{\boldsymbol{B}}}$, samples were permuted (that is, rows of ***X***), and $\hat{{\boldsymbol{B}}}$ was re-inferred using FR-Perturb for each permutation. Permuting rows of ***X*** has no impact on the factorize step, because this step does not involve ***X*** (and the alternative approach of permuting rows of ***Y*** does not affect the individual factors). Thus, only the recover step was performed, and ***U*** was estimated for each permutation, followed by multiplying the null ***U*** by ***W*** obtained from the factorize step to obtain the null $\hat{{\boldsymbol{B}}}$ estimate. In addition, to reduce computational cost, only 500 permutations total were performed. For entries of $\hat{{\boldsymbol{B}}}$ that had *P* = 0 based on these 500 permutations, a skew-*t* distribution was fit to the empirical null distribution for each entry using the selm function from the sn R package, and *P* values were then re-computed for these entries from the fitted distribution. False discovery *q* values were computed using the Benjamini–Hochberg procedure applied to the *P* values for all entries of $\hat{{\boldsymbol{B}}}$.

#### Inference using negative binomial regression

Using the glmGamPoi R package^80^, ***B*** was inferred by separately running differential expression analysis for each perturbation (that is, column of ***X***), where the two groups being compared were droplets containing only non-targeting control guides and droplets containing a guide for the perturbed gene of interest. For droplets containing multiple guides, other guides present in the droplet were ignored when forming these groups. Analytic *P* values and false discovery *q* values were obtained for all effect sizes from the method output.

#### Inference using elastic net

Using the spams R package^79^, the same elastic net inference procedure proposed in Dixit et al.^2^ was used to infer ***B*** from the following models: ${{\boldsymbol{Y}}^{\,\prime}}={{\boldsymbol{X}}^{\,\prime}B}$ for version 1 and ${{\boldsymbol{Y}}^{\,\prime}}={\boldsymbol{XB}}$ for version 2 from above with *λ*_1_ = 0.00025 and *λ*_2_ = 0.00025 (where elastic net finds the solution to $\mathop{\min }\nolimits_{{\boldsymbol{y}}^{\,\prime}}\frac{1}{2}{{||}\,{{\boldsymbol{y}}^{\,\prime}}-{\boldsymbol{X}}\,{\boldsymbol{\beta }}{||}}_{2}^{2}+{\lambda }_{1}{{\boldsymbol{||}}\,{\boldsymbol{\beta }}{\boldsymbol{||}}}_{1}{\boldsymbol{+}}\frac{{\lambda }_{2}}{2}{{\boldsymbol{||}}\,{\boldsymbol{\beta }}{\boldsymbol{||}}}_{2}^{2}$ for each column of ***Y*′**), matching the values used in Dixit et al. Other values for the parameters yielded similar results (Supplementary Fig. 12g). *P* values for all effect sizes were obtained by permuting the rows of ***X*** a total of 10 times and re-estimating ***B*** to generate a null distribution across all values of ***B***, matching the procedure used in Dixit et al.

#### Selecting optimal guide combination for each gene

Four distinct sgRNAs were generated for each perturbed gene. When inferring effect sizes, guides were aggregated by perturbed gene to increase sample size and simplify downstream analyses. When generating the perturbation design matrix ***X***, a cell containing any guide for the gene was labeled as receiving a perturbation for the gene. However, sgRNAs have varying efficiency at KO or KD their target gene, and including guides that do not work will add noise to the effect size inference. To retain only sgRNAs that had measurable effects on their target gene, we retained guides with concordant effect size estimates across random sample-wise splits of the data (that is, the subset of guides to the same gene showing maximal concordance).

Specifically, let *i* represent the index of a given perturbed gene, so that ***x***_***i***_ corresponds to the column of ***X*** that indicates which cells received perturbation *i*, and ***β***_***i***_ corresponds to the column of ***B*** that indicates the effect sizes on all genes’ expression from perturbing gene *i*. For each *i*, 15 different versions of ***x***_***i***_ were generated, corresponding to all possible subsets of the four guides. For each version, any cell receiving a guide within the given subset of guides is labeled as containing a perturbation for the gene, whereas the remaining guides are ignored. Only ***x***_***i***_ in ***X*** was modified, and the remaining columns were kept the same. Next, the dataset of interest was randomly split in half by samples (cells). FR-Perturb was used to infer effect sizes for all perturbed genes within each half. Then, the *R*^2^ of ${\hat{{\boldsymbol{\beta }}}}_{i}$ was computed between the two halves (restricting to only effects with an FDR *q* < 0.2), and the specific guide subset that produced that highest *R*^2^ was retained. The same procedure was repeated for each *i* to learn the optimal guide combination for each perturbed gene.

#### Simulations

Perturb-seq datasets were simulated at various levels of overloading using real expression counts and perturbation effect sizes estimated from our data.

##### Simulating cell-pooled data

To simulate expression data for *n* droplets containing *m* cells each, the expression of *n* × *m* cells (each containing one guide) was first simulated by randomly sampling control cells from our experiment and scaling their expression counts by the fold change effect sizes of a given perturbed gene (estimated from our conventional KO Perturb-seq screen). A 10% probability of receiving a control guide (that is, no change in expression) was simulated to match the proportion of control guides in the real data. Next, the expression counts of *m* cells were randomly averaged at a time to create cell-pooled data.

##### Simulating guide-pooled data

To simulate expression data for *n* cells containing *m* guides each, *m* perturbed genes were randomly selected for each cell, and the expression of a randomly selected control cell was then scaled by the product of the fold change effect sizes of the *m* perturbed genes. As before, a 10% probability of receiving a control guide was simulated.

#### Clustering and dimensionality reduction

For Fig. 5c, dimensionality reduction was performed using PCA on the log(TP10K + 1) expression counts of all cells, where the expression values of each gene are scaled and centered to mean 0 and variance 1.

The rows and columns of Fig. 5d were clustered using Leiden clustering^81^. First, the Euclidian distance between all pairs of genes was calculated by their perturbation effect sizes, and the FindNeighbors function from the Seurat R package^78^ was used to compute a shared nearest neighbor graph from these distances (*k* = 20), followed by the FindClusters function to perform Leiden clustering on the graph with resolution parameter = 0.5, selected by visual inspection of the resulting clusters. GO enrichment analysis of the genes in the resulting clusters was performed with the ClusterProfiler package^82^ with gene sets obtained from the C2 (curated gene sets) and C5 (ontology gene sets) collections of the Molecular Signatures Database^83^.

#### Learning second-order effects for individual perturbation pairs

Second-order interaction effects on gene expression in cell *i* with multiple guides were modeled as:

$$E\left[\log \left({{\boldsymbol{y}}}_{{\boldsymbol{i}}}\right)\right]=\log \left({\boldsymbol{c}}\right)+\mathop{\sum }\limits_{j}^{P}{x}_{{ij}}\,{{\boldsymbol{\beta }}}_{{\boldsymbol{j}}}+\mathop{\sum }\limits_{j}^{P}\mathop{\sum }\limits_{k}^{P}{x}_{{ij}}{x}_{{ik}}{\,{\boldsymbol{\beta }}}_{{\boldsymbol{jk}}}$$

Here, $\log \left({{\boldsymbol{y}}}_{{\boldsymbol{i}}}\right)$ is a vector of length *G* corresponding to the log expression counts of all genes in droplet *i*; *x*_*ij*_ and *x*_*ik*_ are binary scalars indicating whether cell *i* contains a guide for gene *j* and/or gene *k*; ***c*** is a vector of length *G* indicating the expected control expression counts of all genes; ***β***_***j***_ is a vector of length *G* indicating the first-order effect size of guide *j* on the expression of *G* genes; and ***β***_***jk***_ is a vector of length *G* indicating the second-order effect size of guides *j* and *k* on the expression of *G* genes. In matrix form, the above can be represented as:

$$E\left[{\boldsymbol{Y}}{\prime} \right]={\boldsymbol{XB}}+{{\boldsymbol{X}}}_{\left({\bf{2}}\right)}{{\boldsymbol{B}}}_{\left({\bf{2}}\right)}$$

where each row of ***Y*′** equals $\log \left({{\boldsymbol{y}}}_{{\boldsymbol{i}}}\right)-\log \left({\boldsymbol{c}}\right)$; ${{\boldsymbol{X}}}_{\left({\boldsymbol{2}}\right)}$ is an $N\times \left(\genfrac{}{}{0ex}{}{P}{2}\right)$ indicator matrix for whether each cell contains any of $\left(\genfrac{}{}{0ex}{}{P}{2}\right)$ perturbation pairs; and ${{\boldsymbol{B}}}_{\left({\boldsymbol{2}}\right)}$ is an $\left(\genfrac{}{}{0ex}{}{P}{2}\right)\times G$ matrix of second-order interaction effects. ***B*** is known from estimating first-order effects previously, which enables the following equation to be written:

$$E\left[{\boldsymbol{Y}}^{\prime\prime}\right]={{\boldsymbol{X}}}_{\left({\bf{2}}\right)}{{\boldsymbol{B}}}_{\left({\bf{2}}\right)}$$

where ${{\boldsymbol{Y}}}^{\,\prime\prime}={{\boldsymbol{Y}}}^{{{\,\prime}}}-{\boldsymbol{XB}}$. Finally, ${{\boldsymbol{B}}}_{\left({\boldsymbol{2}}\right)}$ is estimated using FR-Perturb in the exact same manner as ***B***. To reduce the large size of $\left(\genfrac{}{}{0ex}{}{P}{2}\right)$, only perturbation pairs that were present in a minimum of five cells were included.

When estimating the significance of entries of ${{\boldsymbol{B}}}_{\left({\boldsymbol{2}}\right)}$, the uncertainty in both ***B*** and ${{\boldsymbol{B}}}_{\left({\boldsymbol{2}}\right)}$ must be accounted for, because the latter depends on the former. Thus, when generating a null distribution for the entries of ${{\boldsymbol{B}}}_{\left({\boldsymbol{2}}\right)}$, the rows of both ***X*** and ${{\boldsymbol{X}}}_{\left({\boldsymbol{2}}\right)}$ were permuted, and ***B*** was re-estimated for each permutation.

#### Learning second-order effects for perturbation modules

##### Intra-modular interactions

A second-order intra-modular interaction effect was estimated for each co-functional perturbation module *M* (that is, group of perturbed genes) on each co-regulated gene program *P* (that is, group of downstream genes) as follows. For each pair of *M* and *P*, cells were partitioned into three sets:

1. Control set. Cells containing only non-targeting control guides or guides for genes without significant effects on *P*. The latter group of guides is included to increase sample size, and all these guides are collectively referred to as ‘control guides’.
2. First-order set. Cells with exactly one guide in *M*, with remaining guides in the cells falling into the ‘control guide’ set.
3. Second-order set. Cells with exactly two guides in *M*, with remaining guides in the cells falling into the ‘control guide’ set.

A mean expression value for *P* was computed for each set (*μ*_0_, *μ*_1_ and *μ*_1,1_, respectively) as the average standardized log(TP10K + 1) expression of all genes in *P* among the cells in the set, with covariates corresponding to read count per cell, percent mitochondrial reads, cell cycle state and number of guides per cell regressed out of the log(TP10K + 1) expression matrix and expression standardized to mean 0 and variance 1. The effect size of the first-order set was computed as ${\beta }_{1}={\mu }_{1}-{\mu }_{0}$ and the interaction effect size of the second-order set as ${\beta }_{\mathrm{1,1}}={\mu }_{\mathrm{1,1}}-2{\beta }_{1}-{\mu }_{0}$. *P* values for all interaction effects were computed by permuting the set membership labels of all the cells and recomputing *μ*_0_, *β*_1_ and *β*_1,1_ for the permuted sets. Standard errors for all interaction effects were computed via bootstrapping, by resampling cells from each of the sets without changing their labels.

##### Inter-modular interactions

Inter-modular interaction effects were computed using a similar approach as above. The 490 total modules were first reduced into 30 disjoint modules using Leiden clustering of a shared nearest neighbor graph defined based on the number of genes shared between gene sets. For two co-functional modules, *M*_1_ and *M*_2_, the first-order effects *β*_1_ and *β*_2_ were computed in the same manner as above. The second-order set was defined as cells with at least one guide from each of *M*_1_ and *M*_2_, with the remaining guides in the cell falling into the ‘control guide’ category, as defined above. The mean expression of the second-order group is *μ*_1,2_. The interaction effect is defined as ${\beta }_{\mathrm{1,2}}={\mu }_{\mathrm{1,2}}-{\beta }_{1}-{\beta }_{2}-{\mu }_{0}$, and *P* values and standard errors were estimated using permutation testing and bootstrapping, respectively.

#### Heritability analyses

Sc-linker^55^ was used as previously described to compute a disease heritability enrichment score for each gene set constructed from the KO and KD perturbation effect sizes or perturbation modules and gene programs. Using sc-linker, SNPs were first linked to genes using a combination of histone marks from the Epigenomics Roadmap^84^ and the activity-by-contact strategy^85^, and then an enrichment score was computed for the SNPs based on the heritability enrichment of the SNPs obtained from stratified LD score regression (S-LDSC^86,87^).

More specifically, for each gene set *G*, a set of weights ${A}_{G}=\left\{{a}_{G,1},{a}_{G,2},\ldots ,{a}_{G,\,j}\right\}$ between 0 and 1 was constructed for each SNP based on the confidence of them influencing any gene in *G*, following the procedure described in Jagadeesh et al.^55^ using activity-by-contact scores^88^ and the Epigenomics Roadmap histone marks^84^ for whole blood samples. For gene sets defined from membership in perturbation modules (M1–M3) or gene programs (P1–P4) (Supplementary Table 2), modules/programs were merged between the KO and KD screens. For gene sets defined based on perturbation effects, each gene was weighted by the effect size of the perturbation on the gene, normalized to lie between 0 and 1. A set of weights ${A}_{{all}}=\{{a}_{{all},1},{a}_{{all},2},\ldots ,{a}_{{all},\,j}\}$ was also constructed, representing the confidence of the SNP influencing any gene across the genome. Next, heritability enrichment estimates ${E}_{G}=\frac{ \% {h}^{2}\left({A}_{G}\right)}{ \% {SNP}({A}_{G})}$ and ${E}_{{all}}=\frac{ \% {h}^{2}\left({A}_{{all}}\right)}{ \% {SNP}({A}_{{all}})}$ were computed for each *A*_*G*_ and *A*_*all*_, respectively, using S-LDSC^86,87^. Here, $\% {h}^{2}\left({A}_{G}\right)=\frac{{\sum }_{j}^{M}{a}_{G,\,j}{\beta }_{j}^{2}}{{\sum }_{j}^{M}{\beta }_{j}^{2}}$ (where ${\beta }_{j}^{2}$ represents the squared effect size of SNP *j* on the phenotype and *M* represents the total number of SNPs) and $\% {SNP}\left({A}_{G}\right)=\frac{{\sum }_{j}^{M}{a}_{G,j}}{M}$. Conceptually, $\% {h}^{2}\left(G\right)$ represents the fraction of the total genetic effect on the phenotype attributed to SNPs in *A*_*G*_, whereas %*SNP*(*G*) represents the effective fraction of SNPs that are contained in *A*_*G*_. Thus, the ratio $\frac{{ \% h}^{2}\left(G\right)}{ \% {SNP}\left(G\right)}$ is essentially the average effect size magnitude on the phenotype for SNPs in *A*_*G*_. Finally, the enrichment score for *A*_*G*_ was computed as ${E}_{G}-{E}_{{all}}$. Subtracting *E*_*all*_ controls for the baseline level of heritability enrichment for SNPs that influence any gene (because most SNPs do not influence any genes). *P* values were obtained for the null hypothesis ${E}_{G}-{E}_{{all}}=0$ using a block jackknife procedure^86^.

#### eQTL analyses

Raw genetic data for 432 European individuals and gene expression data for primary monocytes from these individuals profiled 2 h after treatment with LPS were obtained from Fairfax et al.^27^. For each *cis*–*trans* gene pair, plink^89^ was used to compute marginal association statistics of all SNPs within 1 megabase (Mb) of the promoter of the *cis* gene with the expression of both the *cis* gene and the *trans* gene. All our analyses were restricted to *cis* genes with at least one significant *cis*-eQTL (*q* < 0.05) in the Fairfax et al. dataset. Next, coloc^64^ was applied to the association statistics to estimate the posterior probability (with the default prior) that the *cis* and *trans* gene have a shared eQTL within 1 Mb of the *cis* gene, setting a posterior probability threshold of 0.75 to determine significant co-localization (varying this threshold does not change downstream results; Supplementary Fig. 9d). The posterior probability that each *cis* gene co-localizes with random *trans* genes was also computed. For all analyses, the top 20 principal components (PCs) of the gene expression matrix were included as covariates, matching the covariates included by Fairfax et al. in their *trans*-eQTL analysis and selected based on the fact that they maximize the number of significant *trans*-eQTLs in Fairfax et al. By restricting the *cis* gene to having a significant eQTL and comparing our effects to random genes while keeping the *cis* gene the same, we control for differences in power for detecting *cis*-by-*trans* eQTLs that arise from differential levels of selective constraint on the *cis* gene. In particular, the *cis* genes selected to be perturbed in our screens include many genes under selective constraint (Supplementary Fig. 7a), for which we have decreased power to detect *cis*-by-*trans* eQTLs compared to random *cis* genes.

Bivariate Haseman–Elston regression as implemented in the GCTA software tool^66^ was also used to compute the genetic correlation between the expression of the *cis* gene and the *trans* gene when restricting to the region 1 Mb around the promoter of the *cis* gene. Again, the top 20 PCs of the gene expression matrix were included as covariates. The method outputs a genetic correlation estimate $\hat{r}$ and standard error estimate ${SE}(\hat{r})$ for each *cis*–*trans* gene pair. To obtain a combined genetic correlation estimate for all downstream genes of a given perturbed gene, all $\hat{r}$ estimates were first squared and then combined using inverse variance weighing. The variance of ${\hat{r}}^{2}$ was estimated from ${SE}(\hat{r})$ using the Delta method: ${Var}\left({\hat{r}}^{2}\right)\approx 4{\hat{r}}^{2}{Var}(\hat{r})$.

### Reporting summary

Further information on research design is available in the Nature Portfolio Reporting Summary linked to this article.

## Online content

Any methods, additional references, Nature Portfolio reporting summaries, source data, extended data, supplementary information, acknowledgements, peer review information; details of author contributions and competing interests; and statements of data and code availability are available at 10.1038/s41587-023-01964-9.

### Supplementary Information

Supplementary information Supplementary Figs. 1–12 and Supplementary Note.

Reporting Summary

Supplementary Tables 1–5.

## Data Availability

Raw and processed data for all Perturb-seq screens (including all perturbation effect sizes estimated with FR-Perturb) were deposited in the National Center for Biotechnology Information’s Gene Expression Omnibus under accession number GSE221321 (ref. ^90^). SNP-to-gene links (for running sc-linker) can be found at https://github.com/kkdey/GSSG. GWAS summary statistics can be found at https://data.broadinstitute.org/alkesgroup/sumstats_formatted/. eQTLGen data can be found at https://www.eqtlgen.org/phase1.html. Genotypes and expression data from the Fairfax et al.^27^ study can be found at the European Genome-phenome Archive (https://ega-archive.org/) under study ID EGAS00000000109, although approval is needed to obtain raw data. Gene sets from the Molecular Signatures Database used to run enrichment analysis can be found at https://www.gsea-msigdb.org/gsea/msigdb/collections.jsp.

## Code availability

Software implementing FR-Perturb can be found at https://github.com/douglasyao/FR-Perturb (ref. ^91^).
