# Supplementary material for: Scalable genetic screening for regulatory circuits using compressed Perturb-seq
Source: Nat Biotechnol. 2023 Oct 23;42(8):1282–95. doi: 10.1038/s41587-023-01964-9 (PMC11035494; doi:10.1038/s41587-023-01964-9)
Supplement: Supplementary file 1 — Supplementary Figs. 1–12 and Supplementary Note. [file 41587_2023_1964_MOESM1_ESM.pdf]

nature biotechnology

Article

https://doi.org/10.1038/s41587-023-01964-9

Scalable genetic screening for regulatory circuits using compressed Perturb-seq

In the format provided by the authors and unedited

Supplementary Figures

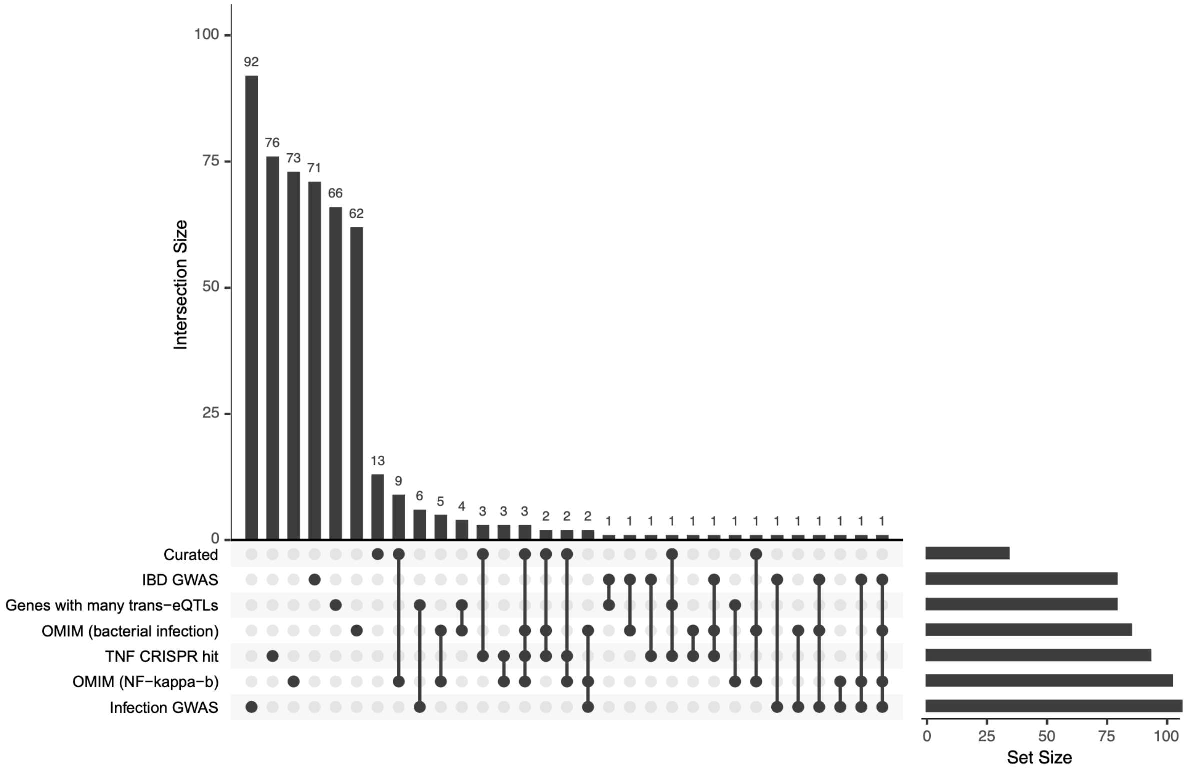

Supplementary Figure 1. Perturbed genes. Upset plot of sources of 598 genes chosen to be perturbed. Curated: manually curated list of canonical LPS response genes. IBD GWAS: genes near fine-mapped variants from GWAS of inflammatory bowel disease[1]. Trans eQTL regulator: genes with a cis-eQTL that is also a trans-eQTL for 4 or more genes in an immune model system[2]. OMIM (bacterial infection): genes involved in Mendelian diseases associated with the term “bacterial infection” in the Online Mendelian Inheritance in Man (OMIM) database[3]. TNF CRISPR hit: top 100 hits from a genome-wide CRISPR screen for regulation of TNF expression after LPS stimulation[4]. OMIM (NF-kappa-b): genes involved in Mendelian diseases associated with the term “NF-kappa-b” in the OMIM database. Infection GWAS: genes associated with the term “infection” in the GWAS Catalog[5].

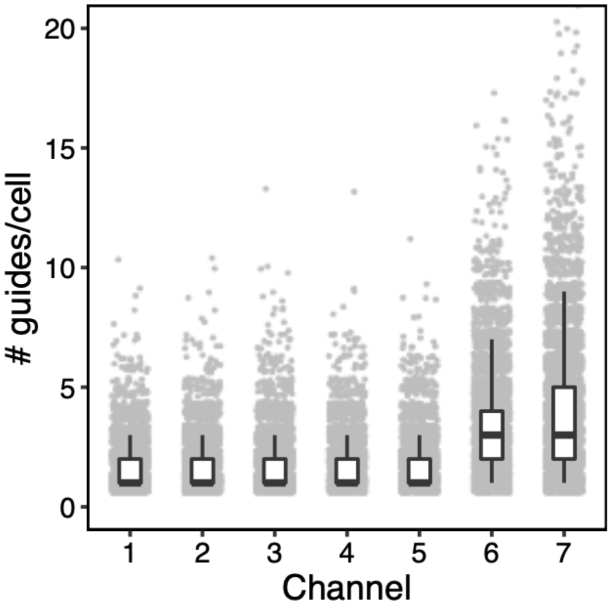

Supplementary Figure 2. Guides/cell in each channel. Distribution of guides/cell across seven 10X channels from guide-pooled screen. Each point represents a cell, with boxes depicting the first quartile, median, and third quartile of values, and the bounds of the whiskers representing 1.5*IQR. For channels 6 and 7, a sequential transduction was performed, resulting in a larger number of guides per cell.

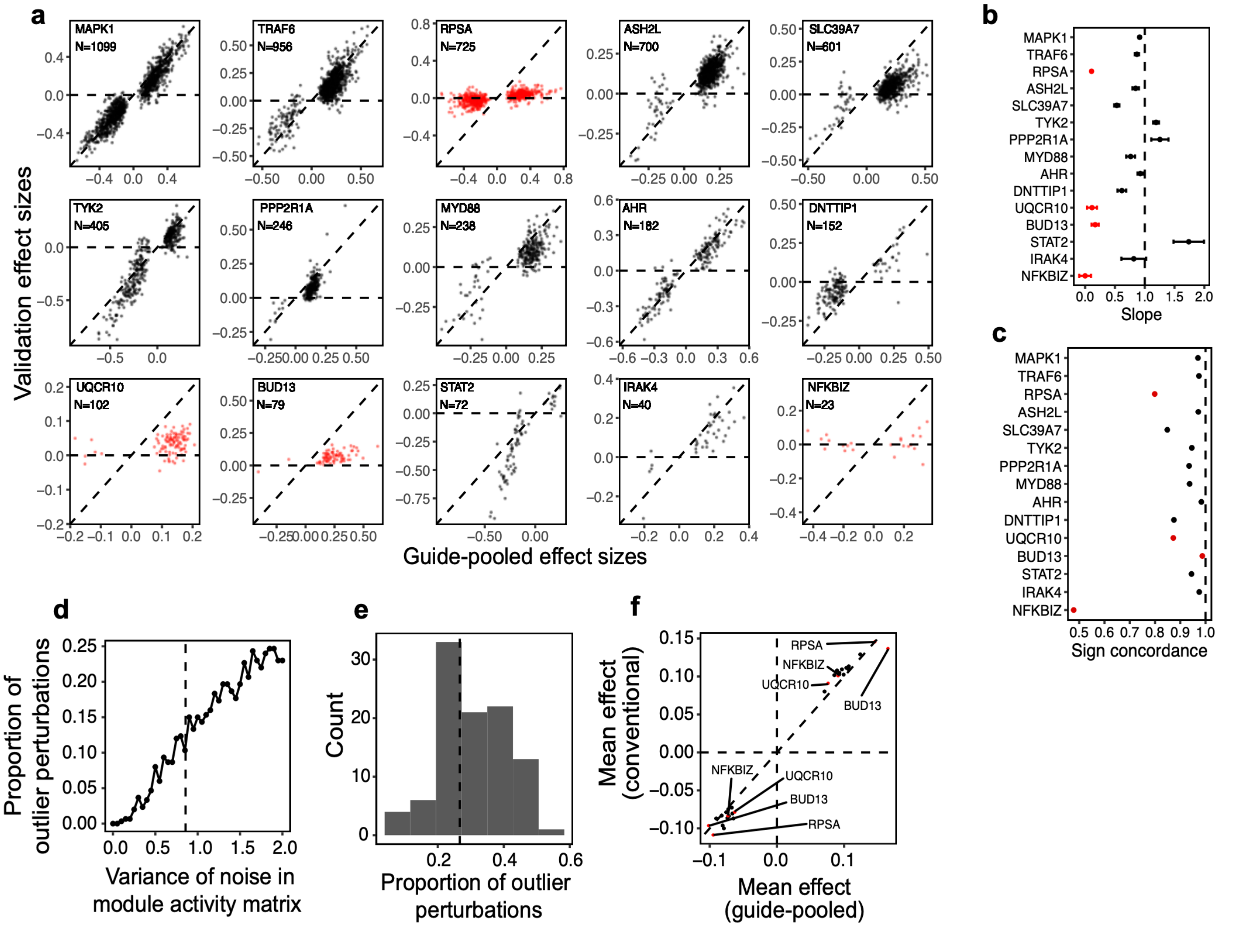

Supplementary Figure 3. Investigating outliers in guide-pooled screen. (**a**) Effect sizes of all significant effects (FDR q < 0.05, N = 5,836) from the guide-pooled screen restricting to 3 or more guides per cell (x-axis) versus the same effects from the validation screen (y-axis), for each of the top 15 perturbed genes (panels; labeled on top left), ranked by number of significant effects. Perturbed genes with a slope < 0.2 are indicated in red. (**b**) Slopes (x-axis; estimated using Deming regression to account for noise in the independent variable) of each scatterplot from **a**. Error bars represent 95% confidence intervals. Red: Perturbed genes with a slope < 0.2. (**c**) Same as **b**, but showing sign concordance rather than slope. (**d**) Proportion of perturbations exhibiting a slope < 0.2 (y-axis) versus the variance of normally distributed noise added to the real module activity matrix from the validation screen (x-axis). Dotted line indicates the "empirical" variance estimated from the real guide-pooled screen (i.e. the average squared difference in elements of the module activity matrix from the conventional vs. guide-pooled screen). Each point is averaged across 20 random simulations. (**e**) Distribution of the proportion of outlier perturbations (slope < 0.2) among the top 15 perturbations across 100 simulated guide-pooled screens. The dotted line indicates the observed proportion of outlier perturbations in the real guide-pooled screen (4/15). (**f**) Mean effect estimates of the same 15 perturbed genes from **a** on all significantly upregulated genes (upper right quadrant) and downregulated genes (lower left quadrant) in the conventional (y-axis) or guide-pooled (x-axis) screen. Mean effects are computed by taking the expression matrix, regressing out covariates, scaling all genes to have

mean 0 and variance 1, centering based on the expression in control cells, then taking the mean value across all cells from the respective screen. The four outlier perturbations from **a** (slope < 0.2) are labeled.

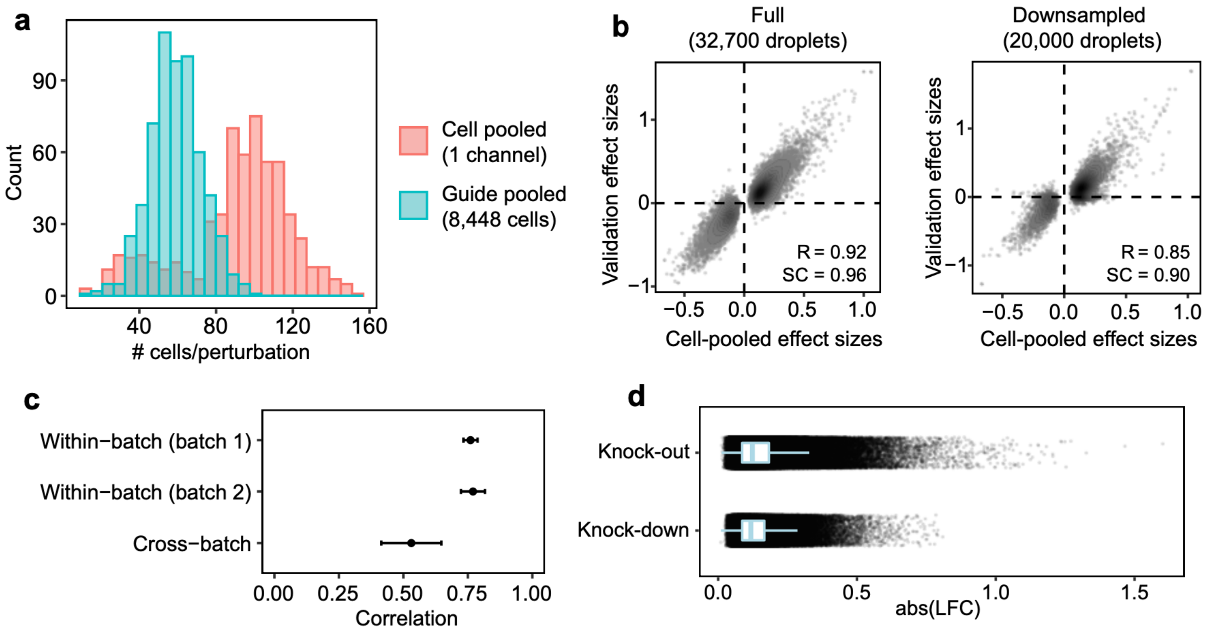

Supplementary Figure 4. External factors differing between guide-pooled and cell-pooled screens. (**a**) Distribution of the number of cells containing a guide targeting each perturbed gene in the cell-pooled (red) and guide-pooled (blue) screens. (**b**) Effect sizes in the cell-pooled (x-axis) and conventional (y-axis) screens for all significant effects (FDR q < 0.05) from the full cell-pooled screen (left) and the cell-pooled screen down-sampled to the same number of cells per perturbation as the guide-pooled screen (right) versus the same effects in the conventional screen. R, Pearson's correlation. SC, sign concordance. (**c**) Pearson's correlation of effect sizes (x-axis) within- and cross-batch (y-axis) from two batches of the conventional knock-down screen. Batches were downsampled to be the same number of cells (27,954 cells each), then randomly split in half. FR-Perturb was used to estimate effects in each split, then the correlation between the top 10,000 most significant effects was computed within and across batches. Error bars represent standard deviation across 5 random splits. (**d**) Distribution of absolute log fold changes for all significant effects (q < 0.05) from the combined (compressed and conventional) knock-out and knock-down screens. Boxplots represent the median and first/third quartiles of points, while the bounds of the whiskers represent 1.5*IQR.

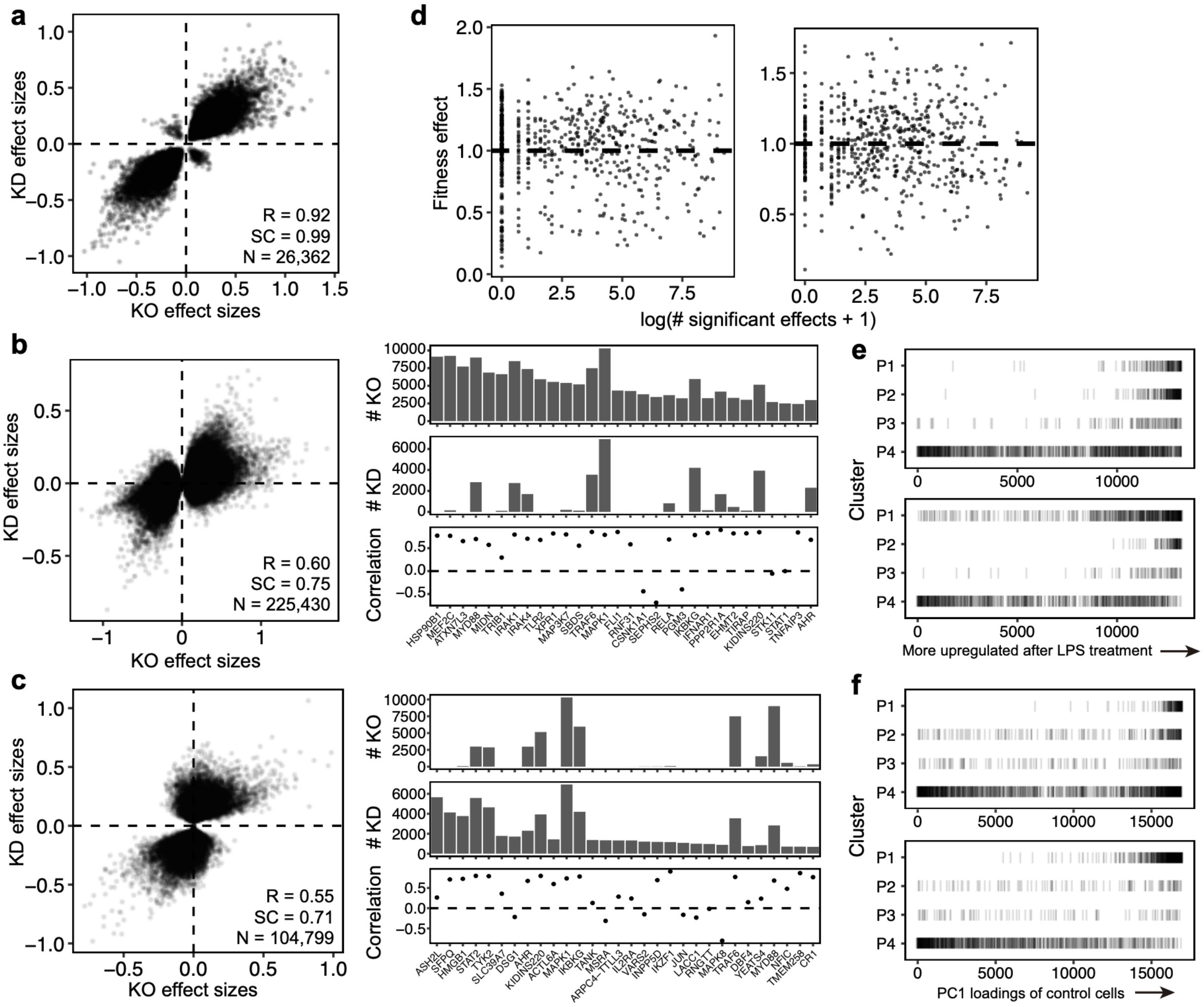

Supplementary Figure 5. Comparison of knock-out versus knock-down screens. Scatterplots of knock-out effect sizes (x-axis) versus knock-down effect sizes (y-axis) while restricting to effects significant ($q < 0.05$) in both screens (**a**), effects significant in only the knock-out screen (**b**), and effects significant in only the knock-down screen (**c**). For **b** and **c**, the correlation of effects stratified by individual perturbed genes is also shown (right plots), with the number of total significant effects for each perturbed gene in the knock-out (top barplot) and knock-down (bottom barplot) screens. (**d**) Relationship between fitness (y-axis) and the number of significant downstream effects (x-axis) for each perturbed gene in the knock-out (left) and knock-down (right) screens. The fitness effect of each perturbed gene is computed as the ratio of reads between the input guide pool and the guides after infecting cells and treating with LPS (while normalizing to the guide distribution in the input pool). (**e**) Enrichment of genes upregulated after LPS treatment in the four main gene programs (**Fig. 5d**) from the knock-out (top) and knock-down (bottom) screens. X-axis: genes sorted based on their expression fold changes before vs. after treatment with LPS, with more upregulated genes toward the right. Genes in the indicated gene programs are represented with a black bar. (**f**) Same as **e**, but sorting genes based on the PC1 loadings of genes from PCA performed on only unperturbed control cells.

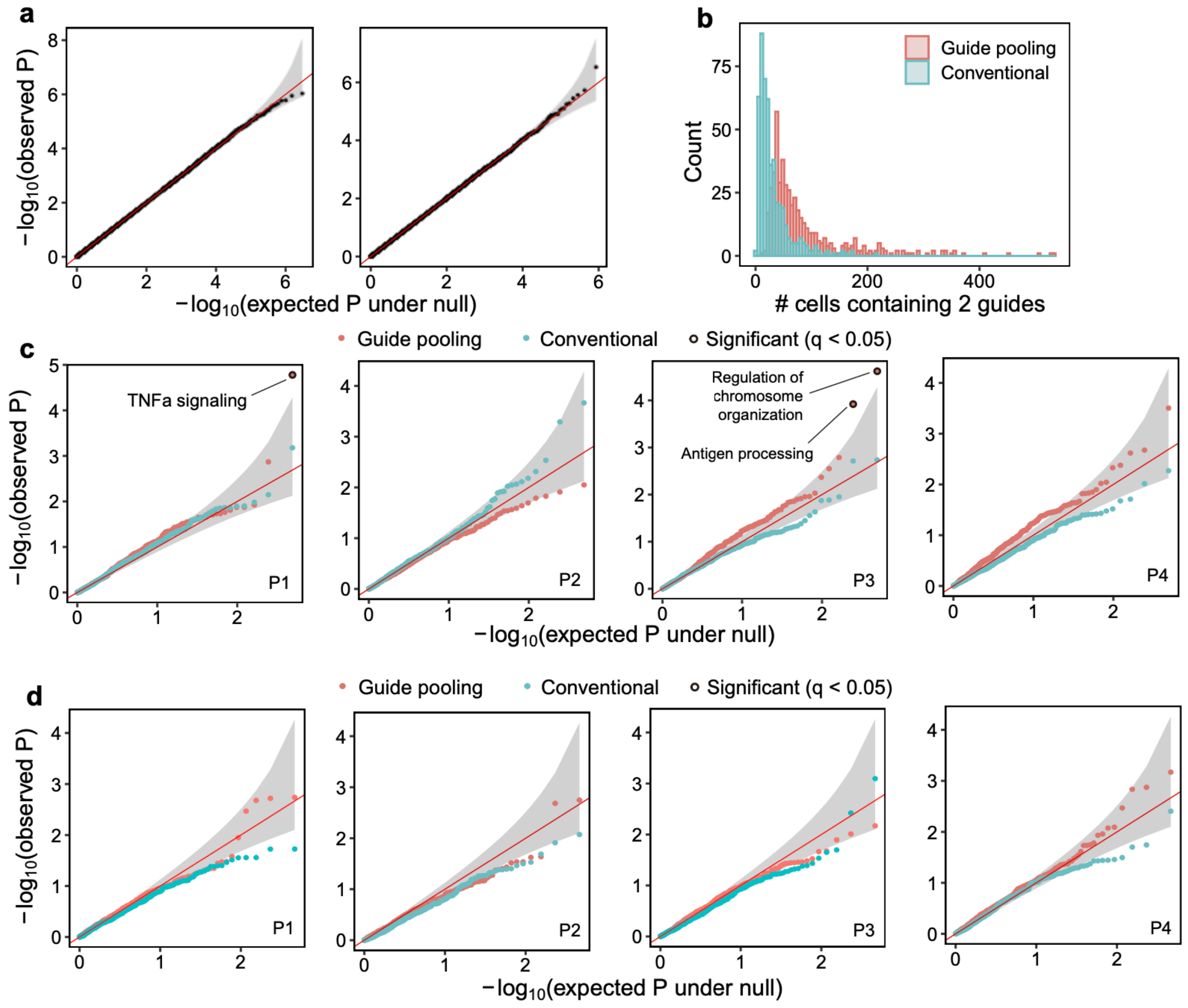

Supplementary Figure 6. Additional analyses of genetic interaction effects. (**a**) Quantile-quantile plots comparing the distribution of observed interaction p-values for all perturbation pairs present in at least 5 cells on all downstream genes for the guide-pooled screen (left; 186 pairs * 16,268 downstream genes = 3,025,848 effects) and conventional screen (right; 46 pairs * 18,617 downstream genes = 856,382 effects) versus a uniform distribution under the null (x-axis). P-values are two-sided and obtained from permutation testing. Grey region indicates the 95% confidence interval under the null. (**b**) Histogram of cells per co-functional modules (out of 490 total) containing at least 2 genes in the module. (**c**) Quantile-quantile plots comparing the distribution of observed intra-modular interaction p-values on the four gene programs (y-axis) versus a uniform distribution under the null (x-axis). P-values are two-sided and obtained from permutation testing. Significant p-values ($q < 0.05$) are indicated with a black border and are labelled with the module name. Grey region indicates the 95% confidence interval under the null. (**d**) Same as **c**, but showing inter-modular (435 module pairs) rather than intra-modular interaction p-values.

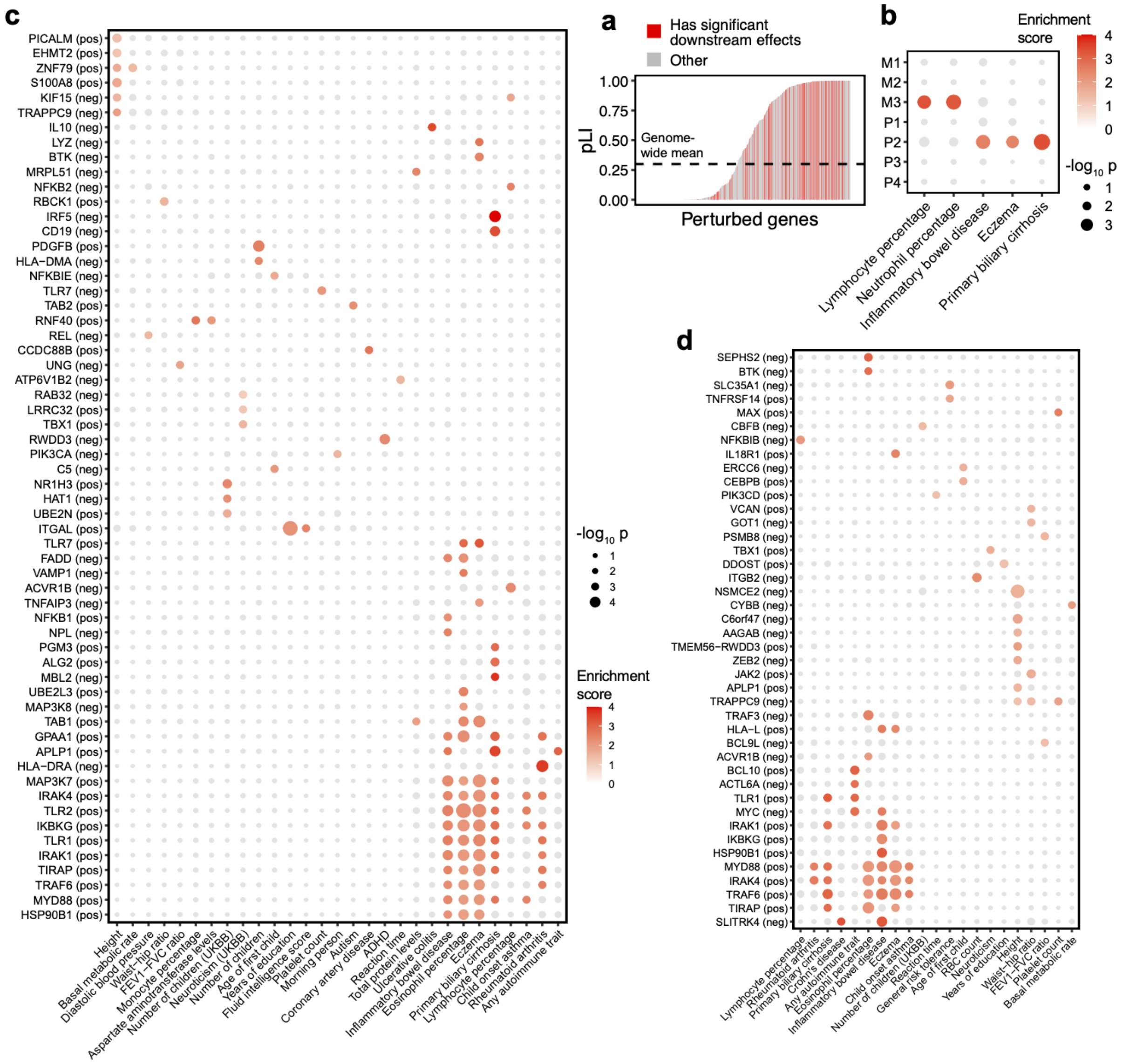

Supplementary Figure 7. Additional heritability analyses. (**a**) Distribution of probability of loss of function intolerance (pLI[6]; y-axis) of all 598 perturbed genes (x-axis). Perturbed genes with significant downstream effects (affecting the expression of >100 genes ($q < 0.05$) in either the knock-out or knock-down screen; N = 159) are indicated in red, with the remaining perturbed genes indicated in grey. Mean pLI across all genes is indicated with a dotted line. (**b**) Heritability enrichment scores (estimated using sc-linker[7]) of perturbation modules M1-3 and gene programs P1-4. Shown are all traits with at least one significant ($p < 0.001$) effect (in red). (**c,d**) Heritability enrichment scores of all perturbation signatures from the knock-out (**c**) and knock-down (**d**) screens with at least one significant ($p < 0.001$) effect on any trait (in red).

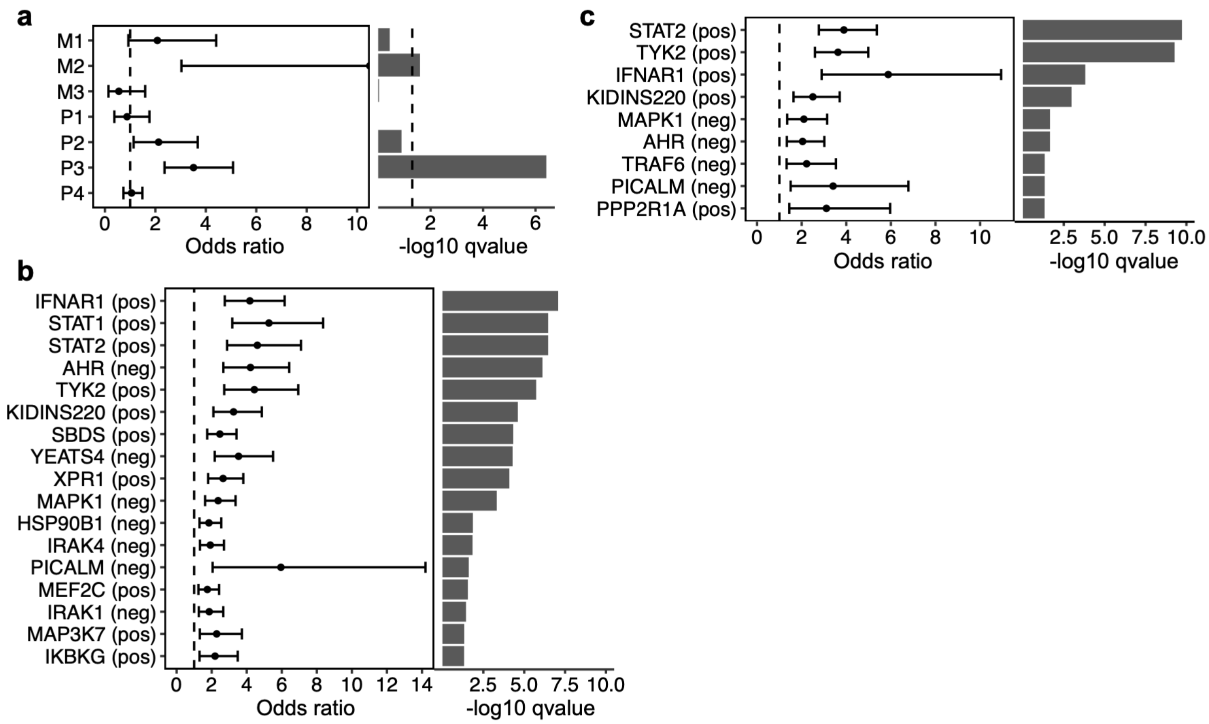

Supplementary Figure 8. Mendelian gene analysis. (**a**) Enrichment (odds ratios, x-axis, left) and -log10 enrichment q-values (x-axis, right) of Mendelian immune genes in gene sets determined by clustering the perturbation-on-gene effect size matrix from **Fig. 5d** (y-axis). P-values are computed using Fisher's exact test, while q-values are computed from the p-values using the Bonferroni-Hochberg correction. Error bars indicate 95% confidence intervals. Dotted line in the right barplot indicates a q-value of 0.05. (**b,c**) Same as **a**, but showing the enrichment of Mendelian immune genes in gene sets constructed from genes significantly downregulated ("pos") or upregulated ("neg") by knock-out (**b**) or knock-down (**c**) of the target gene, matching the gene sets analyzed in **Fig. 6a**.

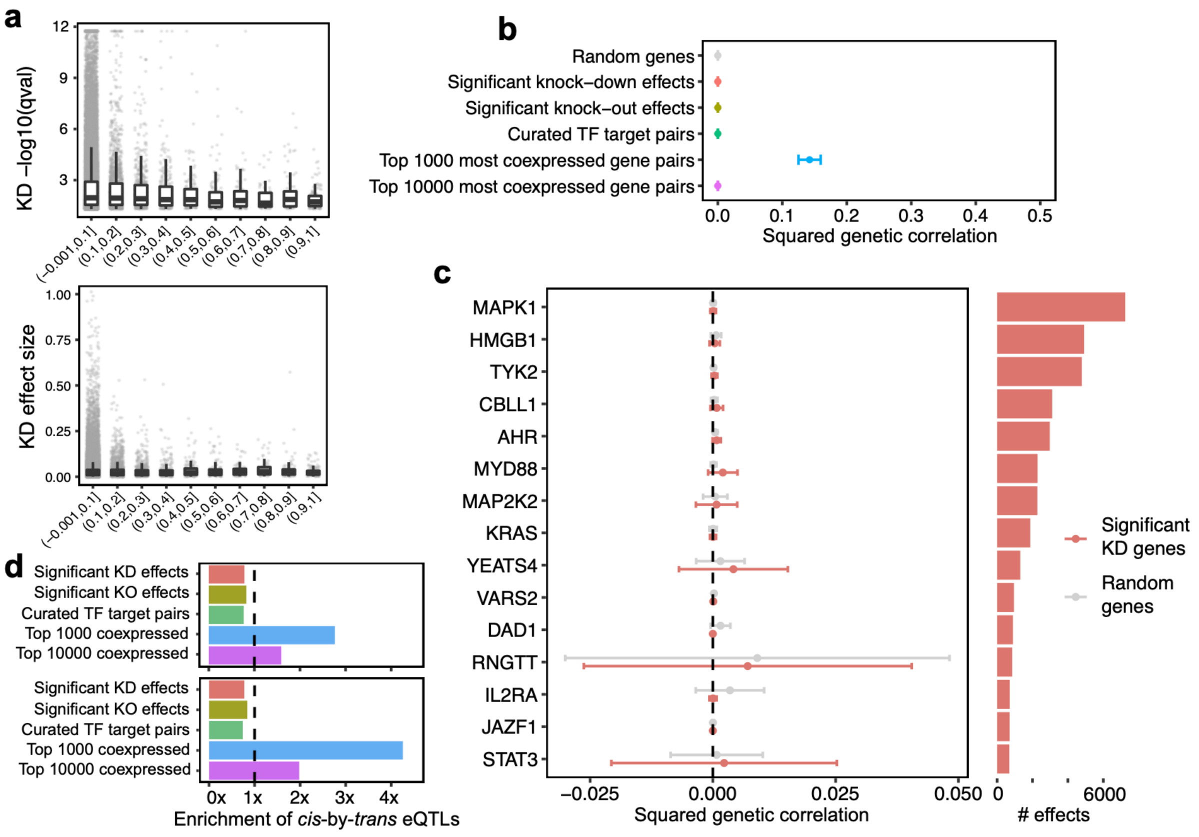

Supplementary Figure 9. Additional eQTL analyses. (**a**) Relationship between knock-down significance (top) or effect size magnitude (bottom) versus probability of observing *cis*-by-*trans* eQTL in the Fairfax dataset (x-axis). Boxplots represent the median and first/third quartiles of points, while the bounds of the whiskers represent 1.5*IQR. (**b**) Genetic correlation estimates across various sources of gene-gene relationships. Estimates are meta-analyzed across all gene pairs. Error bars represent standard errors. (**c**) Genetic correlation estimates for significant gene-gene pairs from the knock-down screen stratified by perturbed gene. Barplot indicates the total number of significant downstream genes for each perturbed gene. Error bars represent standard errors. (**d**) Same as **Fig. 6d**, but varying the posterior probability threshold of determining a significant *cis*-by-*trans* eQTL to 0.25 (top) and 0.5 (bottom).

Knock-out (odds ratio: 0.66)

	Perturb-seq effect	Not Perturb-seq effect
Cis-by-trans eQTL	148	4776
Not cis-by-trans eQTL	52228	1104594

Knock-down (odds ratio: 0.98)

	Perturb-seq effect	Not Perturb-seq effect
Cis-by-trans eQTL	92	4036
Not cis-by-trans eQTL	23831	1021742

Supplementary Figure 10. eQTL analyses using eQTLGen. Overlap between significant cis-by-trans eQTLs from eQTLGen (Bonferroni-corrected p-value < 0.05 for effects on both the cis and trans gene) and significant Perturb-seq gene pairs (q-value < 0.05) in the combined knock-out (left) or knock-down (right) screens. All cis genes are restricted to those that both have a significant cis-eQTL in eQTLGen and at least one significant perturbation effect in Perturb-seq. All trans genes are restricted to those that are present in both the eQTLGen dataset and the Perturb-seq screen.

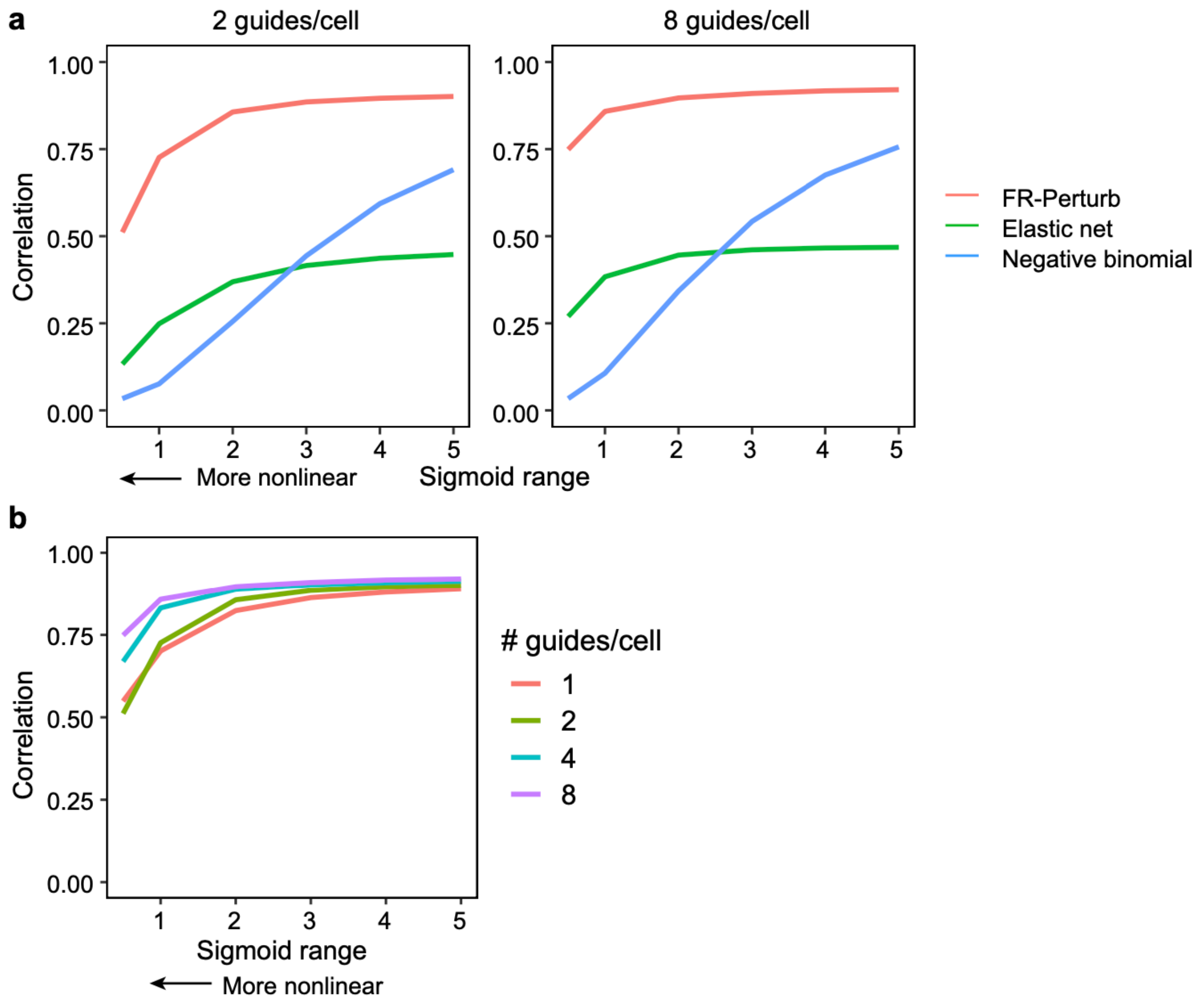

Supplementary Figure 11. **Performance of inference methods in simulations with nonlinear effects.** Simulations were conducted with real effect sizes from the conventional knock-out experiment. Sums of effect sizes were passed through a scaled sigmoid function $f(x) = -m + \frac{2m}{1+e^{-x}}$, where m is the user-specified maximum absolute log fold change of expression for any gene and x is the sum of effect sizes. 25,000 cells were simulated. Simulations were otherwise conducted in the same manner as **Extended Data Fig. 6** (**Methods**). (**a**) Performance of FR-Perturb, elastic net regression, and negative binomial regression (quantified as the correlation of the top 10,000 effects with the true effects; y-axis) when varying the degree of nonlinearity (m from above, where smaller m = faster saturation of effect sizes and thus more nonlinearity; x-axis). (**b**) Performance of FR-Perturb with at different # of guides per cell when varying the degree of nonlinearity.

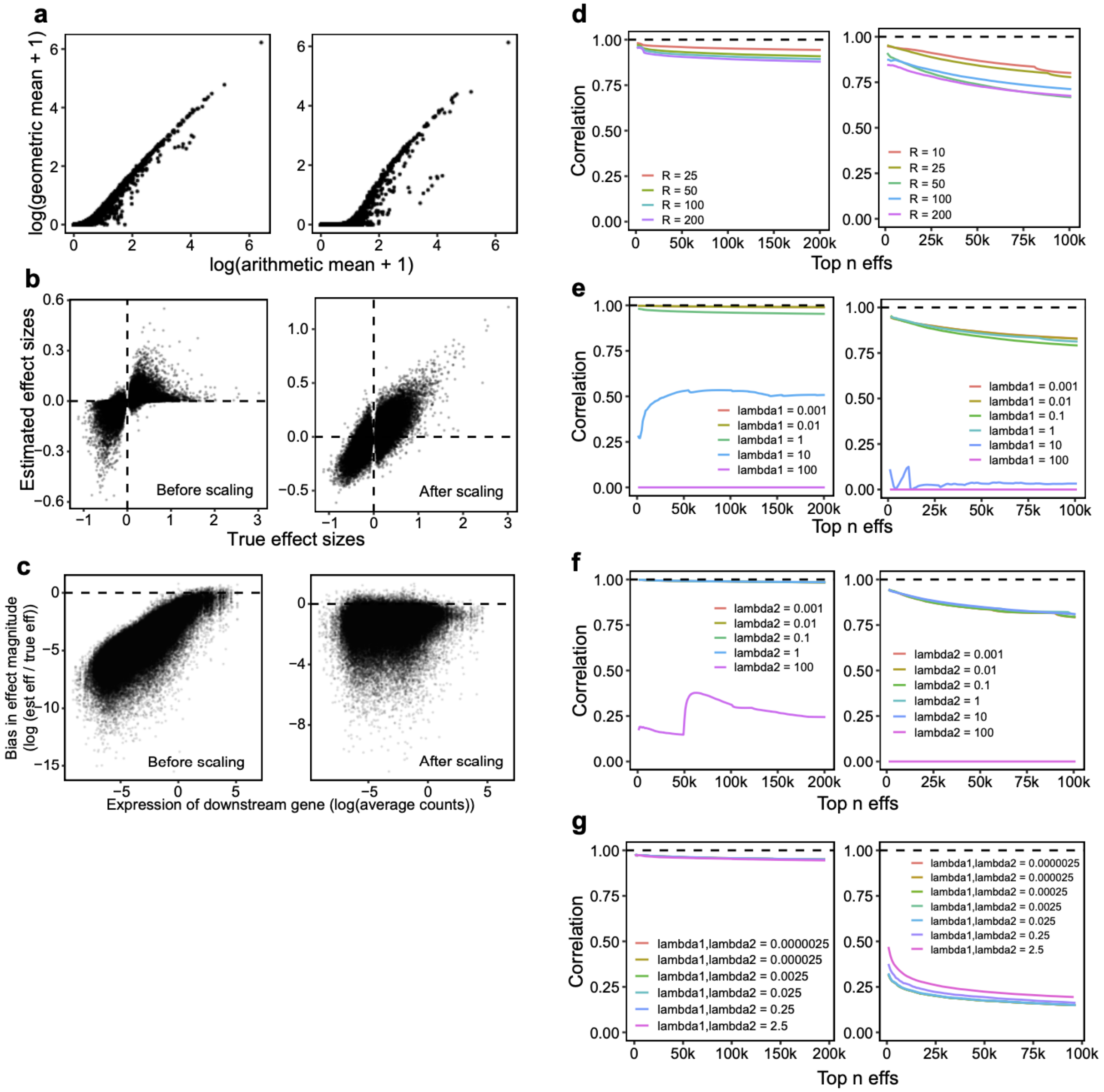

Supplementary Figure 12. Evaluating inference procedures. (**a**) Arithmetic versus geometric mean of expression. Each point represents an individual gene's mean expression across 3 (left) or 10 (right) random control cells from our conventional knock-out screen. (**b**) Impact of scaling effects from FR-Perturb in simulations. Scatterplots of true effect sizes (x-axis) versus estimated effect sizes (y-axis) in simulated cells before versus after scaling effects (**Methods**). True effect sizes were taken as the effects estimated from the knock-out screen, with 50,000 cells simulated. (**C**) Bias in effect size magnitude in estimated effects from **b** (y-axis) versus the expression level of the downstream gene (x-axis). (**d**) Impact of varying the hyperparameters in FR-Perturb. (Left) Correlation of top *n* most significant effects from the knock-out screen with the same effects estimated using the default FR-Perturb hyperparameters (R=10, lambda1=0.1, lambda2=10) when varying R. (Right) Correlation of top *n* most significant effects from the cell-pooled screen with the same effects from the conventional knock-out screen when varying R. (**e,f**) Same as **d**,

but varying lambda1 (**e**) and lambda2 (**f**) rather than R. We note that the poor performance of large values of lambda1 and lambda2 is primarily due to most of the inferred effect sizes being zero. (**g**) Impact of varying hyperparameters of elastic net. (Left) Correlation of top *n* most significant effects from the knock-out screen with the same effects estimated using the default elastic net hyperparameters (lambda1=0.00025, lambda2=0.00025) when varying lambda1 and lambda2. (Right) Correlation of top *n* most significant effects from the cell-pooled screen with the same effects from the conventional knock-out screen when varying lambda1 and lambda2.

Supplementary Note

Section 1. Estimating q and r from our data

We provide rough estimates of two parameters that influence the theoretical performance of FR-Perturb, namely r (i.e. the rank of the perturbation by gene effect matrix after removing unstructured noise, or its "intrinsic dimensionality") and q (i.e. the number of nonzero entries in the perturbation-on-module effect matrix), from our conventional knock-out screen.

To approximate r, we used the Poisson mixture method described in Haro et al.[8] and implemented in the intrinsicDimension R package[9]. Applying this method to the perturbation by gene effect size matrix estimated by either negative binomial regression or elastic net regression (**Methods**) from our conventional knock-out experiment (excluding the matrix estimated by FR-Perturb since we manually set the dimensionality of this matrix as a hyperparameter), we found that the intrinsic dimensionality was 56 (from negative binomial regression) and 47 (from elastic net regression) out of a maximum of 598 (i.e. the total number of perturbations).

To approximate q, we examined the distribution of the elements of the gene by module activity matrix estimated by FR-Perturb (**Methods**). We found that the top 206 elements of this matrix (3.4% of 6,000 total elements) explain 50% of the variance in the overall matrix. We note that this distribution is influenced by our choice of the sparsity hyperparameter for the "recover" step of FR-Perturb.

Both our estimates of r and q are highly tentative given the large amount of overall noise in our perturbation effect size matrices (and in the case of q, dependence on the hyperparameters of FR-Perturb). In practice, we found that setting hyperparameters r = 10 and λ_1 = 10 (related to q) performed the best based on maximizing cross-validation accuracy across datasets. However, a wide range of values for r and λ_1 spanning several orders of magnitude produced similar inference results (**Supplementary Fig. 12**), demonstrating that our choice of these values was not critical to the performance of our approach.

Section 2. Not all genetic interaction effects will bias guide pooling

One of the requirements for guide pooling to produce unbiased effect size estimates is the absence of *systematic* deviation from additivity of effects when multiple perturbations are present in the same sample. Below, we discuss why most genetic interaction effects are unlikely to lead to this model violation. We also present results from simulations of this model violation, showing that guide pooling still leads to efficiency gains even with substantial nonlinearity in effect sizes.

Because each cell receives a random and essentially unique combination of perturbations, individual interaction effects will tend to cancel each other out when averaging across all cells (and thus will not impart bias on the learned effect sizes), so long as there are an equal number of positive and negative interactions. This result (i.e. equal number of positive and negative interactions) is observed to hold true for genome-wide second-order interaction effects on fitness in yeast[10]. We also note that in a companion manuscript to ours[11], an analogue of guide pooling (multiple small molecules/sample) was tested in a very different model system (organoids

stimulated with multiple ligands / small molecules) in which we might expect frequent interactions, and was shown to be effective.

Nonetheless, systematic bias in interaction effects (e.g. a large imbalance in the number of positive vs. negative effects) can realistically arise in some circumstances like the presence of "global buffering effects," such as when the phenotype never exceeds a certain magnitude regardless of the sum of the first order effects of perturbations in the sample. In simulations of this scenario, we found that guide pooling still outperforms conventional screening (**Supplementary Fig. 11**), though the efficiency gains are dampened compared to the scenario without systematically nonlinear interactions.

Section 3. Analysis of pre-stimulated cells
We collected sequencing data for 13,297 cells without stimulation with LPS alongside the LPS-stimulated guide-pooled and conventional knock-down screen. These cells received the same pool of 600 perturbations as LPS-stimulated cells and include one 10X channel each of cells infected at low MOI (matching the conventional screen) and high MOI (matching the guide pooled screen). We performed differential expression analysis comparing all pre-stimulation cells to the LPS-stimulated cells. The top 20 most significantly upregulated genes include classic LPS response markers of inflammation and/or genes involved in core inflammatory/antiviral pathways, including NFKB1, NFKBIA, CCL4L2, CXCL10, CCL3L1, TNFAIP2, TNFAIP3, IRF1, IFIT3, and TRAF1 (**Extended Data Fig. 2a**). We also found that among the downstream genes that are most strongly modulated by all perturbations in our screen, most of them were also significantly upregulated before vs. after treatment with LPS (**Extended Data Fig. 2b**), suggesting that the perturbations in our screen directly affect cellular processes that are modulated by LPS treatment.

Section 4. Outliers in guide pooling comparison
In **Fig. 4e**, we observed visual outliers in the scatterplot of effects between the guide-pooled vs. validation screen. In this section, we show that these outliers arise from noise – specifically, noise in perturbation-on-module effect estimates, which after multiplication with the module dictionary matrix results in correlated noise in perturbation-on-gene effect estimates. We also show that these outliers are unlikely to be caused by genetic interaction effects, which in theory could also cause some effects to differ substantially between the guide-pooled vs. conventional screen.

Evidence for correlated noise in effect size estimates. First, by stratifying significant effects by perturbed gene, we show that the visual outliers in Figure 4E come entirely from a small proportion of perturbed genes where the slope of effects deviates substantially from 1. With a threshold of slope < 0.2 (threshold selected arbitrarily), we identify 4 perturbed genes (out of 15 total with at least X significant effects in the guide-pooled screen) that fall under this threshold (**Supplementary Fig. 3a-c**). We refer to these perturbations as "outlier perturbations."

Next, we conducted two simulations showing that realistic amounts of noise in perturbation-on-module effect estimates can lead to the presence of outlier perturbations.

(1) We added varying levels of normally distributed noise to the real perturbation-by-module matrix from the conventional screen, then counted the proportion of outlier perturbations

after multiplying this matrix by the real module-by-gene dictionary matrix (slope < 0.2 comparing the effect sizes with vs. without noise added). We observed that the proportion of outlier perturbations was nonzero with even a small amount of noise added (**Supplementary Fig. 3d**).

(2) We simulated expression counts in guide-pooled cells at a sample size approximately matching our real guide-pooled screen (10,000 cells; 3 guides per cell) from real effect sizes from the conventional knock-out screen. We simulated these counts using the same procedure as **Extended Data Fig. 6** (**Methods**). We then estimated the effect sizes from the simulated cells using FR-Perturb and counted the proportion of outlier perturbations among the top 15 perturbations (slope < 0.2 comparing the estimated vs. true effects). Across 100 random simulations, we found that 77 of them produced outlier perturbations with at least the same frequency as the real guide-pooled screen (4 or greater out of 15) (**Supplementary Fig. 3e**).

Lack of evidence for outlier effects arising from genetic interactions. We investigated whether genetic interaction effects can explain the outlier perturbations by examining whether outlier perturbations were still present when estimating effect sizes using a method other than FR-Perturb (in which case the phenomenon of correlated noise due to estimation of gene modules would not be a factor). In an analysis similar to that of **Fig. 5f**, we computed the mean effects of the top 15 perturbations (of which 4 were outliers) on all significantly upregulated and downregulated genes from **Supplementary Fig. 3a**. We observed that mean effects of the outlier perturbations did not differ significantly between the guide-pooled vs. conventional screen (**Supplementary Fig. 3f**).

Section 5. Cost savings of guide-pooling and cell-pooling over existing approaches

Compressed Perturb-seq using cell-pooling led to a 4 to 20-fold cost reduction over existing approaches in our experiments, while guide-pooling led to a 10-fold cost reduction, based on matching the number of samples needed to obtain equivalent out-of-sample validation accuracy. These numbers factor in both our experimental changes (cell-pooling or guide-pooling) combined with the use of FR-Perturb to infer effects, compared to existing experimental approaches combined with the use of existing methods to infer effects (elastic net or negative binomial regression).

For cell-pooling, we account for the fact that cell-pooled channels require deeper sequencing than conventional channels (4x per channel in our case): assuming that one cell-pooled channel is equivalent to 10 conventional channels and costs are equally divided between library preparation and sequencing in the conventional screen (as was the case in our screen), this implies a relative cost of 0.5 * (0.1x library preparation costs + 4 * 0.1x sequencing costs) = 0.25x, or a 4x reduction, of cell pooling. We also provide an estimate based on our analysis that only 1/5 of a cell-pooled channel is sufficient to achieve the same validation accuracy as 10 conventional channels, resulting in a relative cost of 0.5 * (0.2 * 0.1x library preparation costs + 0.2 * 4 * 0.1x sequencing costs) = 0.05x, or a 20x reduction. Because we did not generate enough conventional data to match the performance of a single cell-pooled channel (which cannot be sub-divided in a real experiment), we report the cost reduction from cell pooling as a range rather than an exact estimate.

On the other hand, guide pooling produces composite samples at the level of cells and does not require increased per-channel sequencing costs, so the cost reduction can be directly computed as the ratio of required conventional cells to guide-pooled cells (10x in our case).

Section 6. Guide-pooling vs. cell-pooling

In this section, we discuss the strengths and limitations of the two compressed designs relative to conventional designs, and to each other.

Although guide pooling performed nominally worse than cell pooling in our analysis (correlation of 0.92 *vs*. 0.80 with validation screen; **Fig 3e** *vs*. **4e**), this comparison is confounded by various external factors, including smaller sample size of the guide pooled screen, weaker effects from knock-down used in guide pooling versus knock-out used in cell pooling, and additional biological variation in the guide pooled screen because the cells for the conventional validation screen were grown separately vs. the guide pooled screen, while the cells were from the same cell pool in the cell pooled screen vs. validation screen (see **section 7** below and **Supplementary Fig. 4**). In principle, genetic interaction effects add additional variance to effect size estimates in guide pooling but not cell pooling, though we cannot directly quantify this variance, particularly in the presence of the other factors mentioned above. Thus, the relative performance of cell pooling *vs*. guide pooling in a general setting cannot be directly quantified by comparing these plots.

Interestingly, cell pooling performed worse than the conventional approach on a per-droplet basis in both held-out real data and simulations (**Methods**, **Extended Data Fig. 6a**), but we still observed overall efficiency gains from cell pooling (**Fig. 3d-f**) because it provided 7-fold more non-empty droplets per channel (rather than providing more cells per droplet per se). Thus, the reduced per-droplet power (which occurs due to dampening of perturbation effect sizes in a manner proportional to the number of cells in a droplet, see **section 8** below) is balanced by increased per-channel power from obtaining more non-empty droplets. We cannot confidently predict the degree of cell pooling needed to achieve the optimal balance between these counteracting effects. For plate-based assays such as sci-Plex[12], our results suggest that pooling cells will decrease per-sample performance without a counteracting gain from obtaining more samples.

Conversely, the efficiency of the guide-pooled (high MOI) design readily scales with the number of guides per cell, with the best performance attained by cells with 4 or more guides in both held-out real data and simulations (**Extended Data Fig. 6b**). Although infection at high MOI can in theory induce global effects from cellular toxicity, this result demonstrates that these effects are minimal compared to the efficiency gains obtained from having more guides represented per cell. Thus, a higher number of guides per cell (achievable via sequential infections or the use of Cas12/13 to knock out/down multiple genes with a single construct[13–15]) should further improve efficiency, regardless of whether the assay is droplet-based or plate-based.

Section 7. External factors influencing guide pooling vs. cell pooling analysis

In this section, we discuss various external factors that influence the relative performance of guide pooling vs. cell pooling in our manuscript. Although cell pooling appears to outperform guide pooling based on the correlation of significant effects with the validation screen (Pearson's

R of 0.92 vs. 0.80; **Fig. 3e** vs. **4e**), the below factors make it so that we cannot draw conclusions about the relative performance of guide pooling vs. cell pooling based on the higher correlation of the cell pooled screen.

Sample size. Our guide-pooled screen (restricting to 3 or more guides per cell) was effectively smaller than the cell-pooled screen (mean of 65 vs. 90 cells containing each perturbation; **Supplementary Fig. 4a**). This difference in sample size resulted from a combination of difficult-to-control variables, including (1) how many droplets were recovered from an overloaded channel, (2) how many cells on average were in each overloaded droplet, and (3) how many guides on average were in each guide-pooled cell.

After down-sampling the cell-pooled screen to the same number of cells per perturbation as the guide-pooled screen (65 cells), we found that the correlation of significant effects between the cell-pooled screen and validation screen decreased from 0.92 to 0.85 (**Supplementary Fig. 4b**), and was substantially closer to the correlation of significant effects between the guide-pooled and validation screen (0.80).

Biological noise. Our guide-pooled screen and corresponding conventional screen were conducted in cells grown separately out of necessity (see **Fig. 2a**), whereas the cell-pooled screen and corresponding conventional screen were conducted using the same batch of cells for convenience. This introduces additional biological noise in the guide-pooling vs. conventional comparison that is not present in the cell-pooling vs. conventional comparison.

Because our conventional knock-down screen was conducted in two separate batches of cells (due to an inadequate number of cells recovered from the first batch) which were then profiled together, we can examine the additional noise in effect size estimates caused by biological differences between batches. We found that the correlation of effects was substantially lower across batches than within batches (**Supplementary Fig. 4c**), demonstrating the presence of biological noise.

Knock-out vs. knock-down. For our cell-pooled and corresponding conventional screen, we used CRISPR-Cas9 to knock-out gene products (through frameshifts), whereas we used CRISPRi with dCas9-KRAB to knock-down transcription in our guide-pooled screen and corresponding conventional screen to avoid cellular toxicity due to excessive double-stranded breaks. Consistent with other studies[16], we observed that knock-out tended to produce stronger downstream effects on gene expression than knock-down, as demonstrated by the (1) greater number of significant effects (251,792 vs. 131,161) and (2) greater average magnitude of effects in our combined knock-out screen vs. knock-down screen ($p < 2.2e-16$, Mann-Whitney U-test; **Supplementary Fig. 4d**). This will result in a greater signal-to-noise ratio for knock-out vs. knock-down at the same sample size, which in turn results in a higher correlation between the top n effects from the compressed vs. conventional screen.

Section 8. Why does cell pooling reduce per-droplet signal?

When matching the droplet count between our cell-pooled and conventional knock-out screens, we observed that cell pooling performs worse (in terms of out-of-sample validation accuracy) than the conventional screen (**Extended Data Fig. 6a**). This appears to contradict the notion that

generating linear combinations of expression should increase per-measurement signal versus directly measuring expression counts[17], but in fact is not entirely unexpected. In this section, we explain why this result occurs.

Consider a simple example where gene i has an average expression of 100 counts (at whatever constant read depth) in unperturbed cells. Now consider three guides a, b, and c which have a true log2-fold change effect of 1, 0, and 0 on gene i respectively. Three cells containing each of guides a, b, and c will have expression values of 200, 100, and 100 for gene i respectively (in expectation). If we put these three cells in a droplet and measure their average expression, we will observe 133 counts in expectation, which is substantially closer to 100 than the 200 counts for the cell containing guide a. Moreover, including additional cells that do not have an effect on gene i in the same droplet will further cause the average to be closer to 100 and thus decrease the signal to noise ratio, an effect that can be partially offset through deeper sequencing, which incurs additional cost. (As an aside, we note that with some forms of measurement, such as fluorescent measurements, but not sequencing-based counts, it may be possible to decrease the noise without increasing costs). On the contrary, for single cells containing all guides a, b, and c, assuming that the effect sizes of multiple guides combine additively in log expression space in the same cell, we will still observe an expression value of 200 for the cell. Adding more guides per cell will not dilute the signal.

Although it is not obvious that the effect size dilution in the cell pooling scenario will overpower the efficiency gains from measuring linear combinations of expression, in practice and in simulations we observed it to be the case (**Extended Data Fig. 6a**). In other words, when performing cell pooling, we are increasing the sample size per droplet by measuring multiple cells per droplet but decreasing the signal to noise ratio, and the latter ends up decreasing the overall per-droplet efficiency of cell pooling.

To summarize, the fact that cell pooling takes linear combinations of expression values across different cells (in which the presence of additional control cells will dilute the overall signal), whereas guide pooling takes linear combinations of effect sizes within the same cell (in which the presence of additional control guides will not dilute the overall signal), causes the performance of cell pooling (but not guide pooling) to drop when increasing the level of overloading.

Section 9. Use of guide pooling to systematically study high-order genetic interaction effects
One major challenge in learning second-order interaction effects (and even more so for higher-order interaction effects) is the very large space of possible gene combinations that must be tested, wherein our guide-pooled approach can in theory lead to exponential increases in efficiency over the conventional approach. We describe these theoretical efficiency gains below.

For simplicity, suppose that we are measuring a scalar phenotype. If N is the number of distinct perturbations and W the order of the interaction effect among the perturbations, then there are $\binom{N}{W}$ total interaction effects to learn. Using the conventional approach, which involves individually perturbing each gene combination in each sample, one needs $O\left(\binom{N}{W}\right) \approx O(N^W)$ samples to learn all interaction effects. This quantity scales exponentially with respect to W and

polynomially (with exponent W) with respect to N. On the other hand, the guide-pooled approach involves perturbing multiple random gene combinations in each sample (cell). This requires only $O\left(k\log\binom{N}{W}\right) \approx O(k\log N^W) \approx O(Wk\log N)$ samples. This quantity scales *linearly* with respect to W and logarithmically with respect to N, making it potentially tractable to systematically study genetic interaction effects at high order.

While the quantity k (which refers to the number of nonzero interaction effects) will also increase with both N and W, it is likely that k will increase much more slowly than $\binom{N}{W}$, as supported by the observation that interaction effects get substantially sparser when going from second to third order interaction effects in yeast[18]. When the goal is to study an M-dimensional phenotype like effect sizes on gene expression, k in the above expression can be replaced by $r + q$ (where r refers to the rank of the $\binom{N}{W} \times M$ interaction effect size matrix and q refers to number of nonzero interaction effects on latent factors of the interaction effect size matrix). Essentially nothing is known about the low-rank structure of genetic interaction effects on gene expression profiles in any model system, though our analyses of inter- and intra-module interaction effects (**Fig. 5f, Supplementary Fig. 6c,d**) provides some initial evidence for the existence of such low-rank structure in second-order interaction effects.

Importantly, these efficiency gains are also contingent on the fact that the additive model holds across all orders. This means, for example, that the phenotype of a sample (cell) containing perturbations for genes i, j, and k should on average be the sum of all first order effects as well as all *pairs* of second order effects between the three perturbations. In practice, very little is known about the structure of higher-order interaction effects and how they systematically combine within a cell, so more study is required to investigate the validity of this model.

Section 10. Mendelian gene analysis

To complement our analysis in **Fig. 6a**, we also computed the enrichment of Mendelian immune genes (395 genes from the International Union of Immunological Societies website (https://iuis.org/committees/iei/, December 2019 dataset) among the same 1,638 gene sets analyzed in **Fig. 6a**, comprising genes significantly upregulated and downregulated by each perturbation, with thresholds for significance set at q-value < 0.2 and abs(LFC) > 0.25. We also included 7 additional gene sets corresponding to genes in the four gene programs (P1-4) and three gene modules (M1-3) from **Fig. 5d**. We computed the enrichment of Mendelian immune genes among these gene sets using Fisher's exact test. When computing odds ratios for M1-3, we labeled the background set of genes as the 598 perturbed genes. When computing odds ratios for P1-P4 and all other gene sets, we labeled the background set of genes as all expressed genes. Our results from this analysis are shown below.

Among the three perturbation modules M1-3, we observed significant enrichment (q < 0.05) of M2 (perturbed genes involved in regulation of the antiviral response) (**Supplementary Fig. 8a**), whereas in our GWAS analysis we only observed significant enrichment of M3 (perturbed genes that suppress the LPS response) (**Supplementary Fig. 7b**). Among gene programs P1-4, we observed significant enrichment of only P3 (expressed genes regulated by M2 genes), in contrast to our GWAS analysis which demonstrated significant enrichment of only P2. Thus, these results demonstrate that different components of the regulatory systems captured by our model are

relevant to human genetic data from opposing sides of the selective spectrum, such that Mendelian and common variants impinge on distinct processes in this system.

Among the 1,638 gene sets constructed from individual perturbations, 26 gene sets had significant enrichment ($q < 0.05$) for Mendelian immune genes (**Supplementary Fig. 8b,c**), closely matching in number the 23 gene sets with significant enrichment in the GWAS heritability analyses (**Fig. 6a**). This includes 17 from the knock-out screen and 9 from the knock-down screen, consistent with our heritability analyses which report a greater number of significant results from knock-out over knock-down. The most highly enriched gene sets came from perturbations whose knock-out/down led to the strongest downregulation of antiviral response (P3), including STAT1, STAT2, IFNAR1, and TYK2 (pos). Other enriched gene sets also include the antiviral response, including the set of genes upregulated by knock-out/down of core LPS genes (IRAK1, IRAK4, IKBKG, MAP3K7, TRAF6).

Section 11. Enrichment of *cis*-by-*trans* eQTLs in the most co-expressed genes suggests the presence of confounding

In **Fig. 6d**, we observed that *cis*-by-*trans* eQTLs are enriched in gene pairs with the highest correlation of expression across samples (*i.e.*, the most co-expressed genes). In this section, we explain why this observation suggests the presence of confounders in the eQTL dataset, even though we followed established protocols to remove them in our analyses (*i.e.*, through the inclusion of top expression principal components).

Under random mating, genetic effects are independent of environmental effects, which enables eQTL effects to be viewed as perturbations on gene expression. However, gene co-expression is almost completely dominated by environmental rather than genetic effects[19], so the fact that we observe an enrichment of *cis*-by-*trans* eQTLs in the most co-expressed genes (**Fig. 6d**) is evidence of an unexpected relationship between genetic correlation and environmental correlation (note that this can occur even if genetic and environmental effects are independent, see below). There are many possible causal scenarios that can lead to a relationship between these quantities. We illustrate three of the simplest scenarios below:

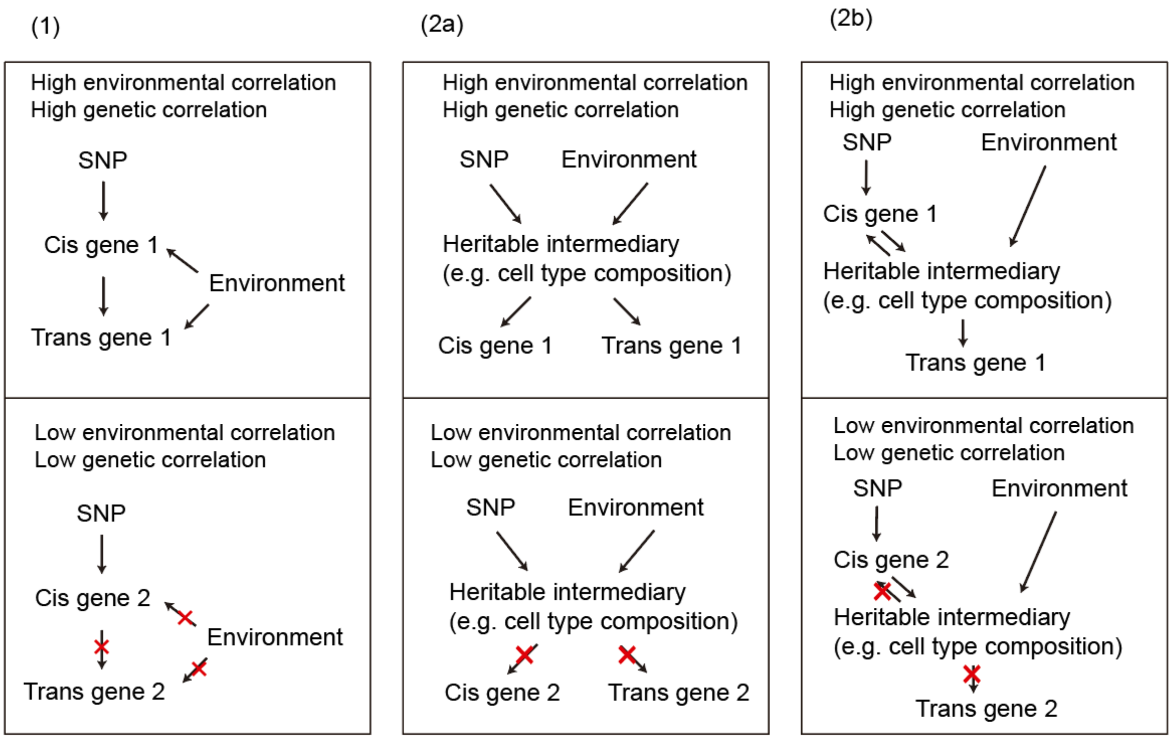

Each column in the above figure corresponds to a single scenario applied to different gene pairs (top and bottom boxes). The top box illustrates the scenario applied to a gene pair with high genetic and environmental correlation, while the bottom box illustrates the same scenario applied to a different gene pair with low genetic/environmental correlation. Scenarios (1) and (2b) have a causal link from the *cis* gene to the *trans* gene, but scenario (2a) does not and is driven by confounding. Although all three scenarios are possible in theory, we discuss below why scenario (2a) is probably the most common one.

Scenario (1) requires that the probability of the *cis* gene having a causal effect on the *trans* gene is related to the environmental correlation between the genes. Although this can in theory be explained by various biologically plausible scenarios (*e.g.*, the *cis* gene only affects the *trans* gene in the presence of a co-factor which is influenced by the environment), this scenario is less parsimonious in light of our observation that *cis*-by-*trans* eQTLs are much more strongly enriched in the top 1,000 most co-expressed gene pairs than the top 10,000 most co-expressed gene pairs (**Fig. 6d**). In other words, this would mean that the hypothetical environmentally-driven co-factor scenario acts much more strongly on the top 0.0003% of gene pairs than the top 0.003% of gene pairs, both of which are a tiny and somewhat arbitrary (from the perspective of biological function) fraction of all possible gene pairs. For the top 0.0003% of gene pairs to be functionally different than the top 0.003% when the degree of co-expression is similar between the two groups, an extremely (and we argue unrealistically) non-linear relationship between genetic and environmental correlation would need to be present.

Meanwhile, scenarios (2a) and (2b) involve the presence of a heritable intermediary between the SNP and the *cis* and *trans* genes which can also be affected by the environment. This intermediary could be intercellular/cell-type heterogeneity (which is known to be a heritable and polygenic trait in blood cells[20]) or a molecular pathway. The intermediary causes some genes'

expression to be more correlated with each other than others. Thus, because gene co-expression is determined by the intermediary and both genetic and environmental factors flow through it, the genes pairs with the highest environmental correlation and genetic correlation are the same gene pairs. This would explain why we observe significantly stronger enrichment of *cis*-by-*trans* eQTLs in the top 1,000 gene pairs than the top 10,000 gene pairs. Between scenarios (2a) and (2b), (2b) requires a bi-directional causal effect between the *cis* gene and the intermediary, so (2a) is more parsimonious.

We note that the presence of substantial intercellular heterogeneity between individuals is well-established in eQTL studies[21], even those in relatively homogenous cell specimens. For example, in Fairfax et al.[2], the inclusion of gene expression principal components to account for intercellular heterogeneity (despite the cells being the same cell type) results in an increase in the number of *cis*-eQTLs detected (from ~4500 to ~7200) and a decrease in the number of *trans*-eQTLs (from ~700 to ~350) (Fig. S2 in Fairfax et al.). This pattern is consistent with the observation that confounding (due to intercellular heterogeneity and/or other factors) decreases the number of detected cis-eQTLs while increasing the number of false-positive *trans*-eQTLs[22], in which case controlling for confounding would reverse these patterns as observed in Fairfax et al. However, we note that the observed decrease in detected *trans*-eQTLs when controlling for gene expression principal components can in theory also be explained by principal components capturing true, widespread intra-cellular *trans* effects. Disentangling spurious inter-cellular effects from true intra-cellular *trans* effects remains a key challenge in *trans*-eQTL studies.

Section 12. Explaining depletion of *cis*-by-*trans* eQTLs among regulatory gene pairs from Perturb-seq

We observed that regulatory gene pairs identified by our Perturb-seq screens are substantially depleted for colocalized *cis*-by-*trans* eQTLs compared to random gene pairs (**Fig. 6d**). In the scenario that Perturb-seq does not capture regulatory relationships in the eQTL dataset, we might expect that Perturb-seq gene pairs are essentially a random selection of genes, and thus we would observe the *same* probability of colocalization of *cis*-by-*trans* eQTL among our Perturb-seq gene pairs versus random gene pairs. However, the fact that we observe a *lower* probability of colocalization of *cis*-by-*trans* eQTLs among Perturb-seq gene pairs versus random gene pairs requires an additional explanation. Two potential explanations are discussed below.

(1) *Perturb-seq gene pairs are less confounded than random gene pairs.* We have previously illustrated that detection of *cis*-by-*trans* eQTLs is likely to be at least partially driven by confounders such as intercellular heterogeneity (see previous section). We have additionally observed that significant Perturb-seq gene pairs are more likely to be under selective constraint than random genes, and that highly co-expressed genes are less likely to be under selective constraint (**Fig. 6e**). Thus, under a model where *cis*-by-*trans* eQTLs are driven by confounding, we expect to see an enrichment of *cis*-by-*trans* eQTLs for co-expressed (and unconstrained) gene pairs, and a concomitant depletion of *cis*-by-*trans* eQTLs for significant Perturb-seq (and constrained) gene pairs. The observed depletion can thus be explained by a negative relationship between the technical confounding and selective constraint, in the presence of *cis*-by-*trans* eQTLs driven by confounding.

(2) *Negative selection on the trans gene in Perturb-seq gene pairs reduces power to detect context-specific cis-by-trans eQTLs compared to random gene pairs*. We observed that *trans* genes in the Perturb-seq gene pairs are under more selective constraint than average genes (**Fig. 6e**). However, under the simplest model of SNPs acting on the *trans* gene through the *cis* gene (see below), this fact alone does not manifest in decreased power to detect *cis*-by-*trans* eQTLs compared to random gene pairs (because our analysis explicitly conditioned on the *cis*/perturbed gene when selecting random gene pairs).

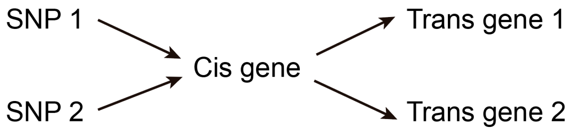

In this model, every SNP that affects the *cis* gene (including SNPs 1 and 2) will also affect both *trans* genes. Suppose that *trans* gene 1 is under strong selective constraint, so that any SNP with an effect on it is selected out of the population, whereas *trans* gene 2 is not under any selective constraint. All SNPs that regulate the *cis* gene (including both SNP 1 and SNP 2) will have decreased frequency in the population, resulting in uniformly decreased power to detect *cis*-by-*trans* eQTLs for both *cis* gene -> *trans* gene 1 and *cis* gene -> *trans* gene 2 (as well as any additional *trans* genes that the *cis* gene regulates). As noted above, when we compared Perturb-seq gene pairs to random gene pairs in our analyses, we kept the *cis* gene constant in all comparisons, thus controlling for differences in power for SNPs that regulate the *cis* gene.

A slightly modified version of the above model is needed to explain our observation of a depletion of *cis*-by-*trans* eQTLs for Perturb-seq gene pairs:

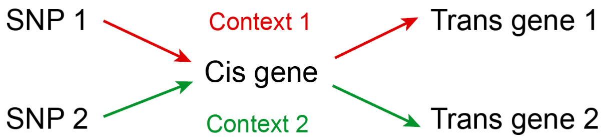

Here, SNP 1 only acts on *trans* gene 1 through the *cis* gene in a given context 1 (which could be a cell or tissue type, transient cell state, intracellular location, etc.), while SNP 2 only acts on *trans* gene 2 through the *cis* gene in a different context 2. Under this model, selection on *trans* gene 1 causes SNP 1 to be selected out of the population but not SNP 2. This will decrease the power to detect *cis*-by-*trans* eQTLs for *cis* gene -> *trans* gene 1 but not *cis* gene -> *trans* gene 2. Thus, differential selection on context-specific *cis*-eQTLs for the same gene (as has been previously hypothesized[23]) could explain why we observe a depletion of *cis*-by-*trans* eQTLs for *trans* genes under selection.

Section 13. Additional simulations

We conducted additional simulations testing the performance of cell-pooling and/or guide-pooling when varying (1) sequencing depth, (2) the distribution of guides per cell, and (3) guide efficiency. These simulations were conducted in an identical manner as **Extended Data Fig. 6** (**Methods**).

Sequencing depth. We observed that the performance of cell/guide pooling drops slightly with lower sequencing depth, but remains relatively robust even with ¼ the sequencing depth as our screens (~9,000 reads per cell in our screens) (**Extended Data Fig. 9a**).

Distribution of guides per cell. In all our other simulations of guide-pooling, we simulated a fixed number of guides per cell for each experiment. In our real guide-pooled screen, we observe a distribution of guides per cell that approximately follows a zero-truncated Poisson distribution (**Fig. 4b**). We observe virtually identical performance of guide-pooling when simulating the number of guides per cell from this distribution rather than a fixed number of guides per cell (**Extended Data Fig. 9b**).

Guide efficiency. We examined the performance of guide-pooling in two scenarios where we varied (1) the efficiency of all guides (**Extended Data Fig. 9c**), or (2) the efficiency of guides within individual cells as a function of the number of guides in the cell – namely, where efficiency decays with more guides per cell, which can realistically occur due to cellular toxicity or limited quantities of Cas9 protein (**Extended Data Fig. 9d**). We found that, as expected, the performance of guide-pooling decreases as a function of guide efficiency. However, in practice, we do not know the true efficiency of guides, nor can we exactly predict how efficiency decays as a function of the number of guides per cell.

Section 14. Number of cells needed to learn second-order interactions

Given that the assumptions discussed in the section "Use of guide pooling to systematically study high-order genetic interaction effects" (from above) hold, the following equation roughly describes the number of cells n needed to learn all second-order interaction effects among p perturbations, given a fixed number of guides per cell k.

$$n = \frac{400 * \binom{p}{2}}{\binom{k}{2}}$$

Here, 400 represents the number of cells needed to learn a single second-order interaction effect with the same power as our conventional screens had to learn first-order effects (~100 cells/perturbations), assuming that four times as many cells are needed to estimate a second-order interaction effect as a first-order effect with the same magnitude[24]. $\binom{p}{2}$ represents the total number of pairwise interactions among p perturbations. $\binom{k}{2}$ represents the number of pairwise interactions that are represented per cell containing k total perturbations.

The number of required cells n as a function of p and k is shown in **Extended Data Fig. 10**. At 100,000 cells (the scale of our conventional screens) and 3 guides per cell (the approximate number of guides per cell in our guide-pooled screen), we are powered to learn all second-order interaction effects among around 39 perturbations. At the scale of a very large Perturb-seq screen like Replogle et al.[25] (1,000,000 cells) and a high level of guide pooling (8 guides per cells), we are powered to learn all second-order interaction effects among 374 gene targets, which is 5.5x the number of gene targets (28x the number of interaction effects) that could be learned in an equally large conventional pair-wise perturbation screen with 2 guides per cell.

References

1. Huang, H. *et al.* Fine-mapping inflammatory bowel disease loci to single-variant resolution. *Nature* **547**, 173–178 (2017).
2. Fairfax, B. P. *et al.* Innate Immune Activity Conditions the Effect of Regulatory Variants upon Monocyte Gene Expression. *Science* **343**, 1246949 (2014).
3. Amberger, J. S., Bocchini, C. A., Scott, A. F. & Hamosh, A. OMIM.org: leveraging knowledge across phenotype–gene relationships. *Nucleic Acids Res.* **47**, D1038–D1043 (2019).
4. Parnas, O. *et al.* A Genome-wide CRISPR Screen in Primary Immune Cells to Dissect Regulatory Networks. *Cell* **162**, 675–686 (2015).
5. Buniello, A. *et al.* The NHGRI-EBI GWAS Catalog of published genome-wide association studies, targeted arrays and summary statistics 2019. *Nucleic Acids Res.* **47**, D1005–D1012 (2019).
6. Karczewski, K. J. *et al.* The mutational constraint spectrum quantified from variation in 141,456 humans. *Nature* **581**, 434–443 (2020).
7. Jagadeesh, K. A. *et al.* Identifying disease-critical cell types and cellular processes by integrating single-cell RNA-sequencing and human genetics. *Nat. Genet.* **54**, 1479–1492 (2022).
8. Haro, G., Randall, G. & Sapiro, G. Translated Poisson Mixture Model for Stratification Learning. *Int. J. Comput. Vis.* **80**, 358–374 (2008).
9. Johnsson, K. Structures in High-Dimensional Data: Intrinsic Dimension and Cluster Analysis. (Centre for Mathematical Sciences, Lund University, 2016).
10. Costanzo, M. *et al.* A global genetic interaction network maps a wiring diagram of cellular function. *Science* **353**, (2016).
11. Mead, B. E. *et al.* Compressed phenotypic screens for complex multicellular models and high-content assays. 2023.01.23.525189 Preprint at https://doi.org/10.1101/2023.01.23.525189 (2023).
12. Srivatsan, S. R. *et al.* Massively multiplex chemical transcriptomics at single-cell resolution. *Science* **367**, 45–51 (2020).
13. Zetsche, B. *et al.* Cpf1 Is a Single RNA-Guided Endonuclease of a Class 2 CRISPR-Cas System. *Cell* **163**, 759–771 (2015).
14. Campa, C. C., Weisbach, N. R., Santinha, A. J., Incarnato, D. & Platt, R. J. Multiplexed genome engineering by Cas12a and CRISPR arrays encoded on single transcripts. *Nat. Methods* **16**, 887–893 (2019).
15. Wessels, H.-H. *et al.* Efficient combinatorial targeting of RNA transcripts in single cells with Cas13 RNA Perturb-seq. *Nat. Methods* **20**, 86–94 (2023).
16. Rosenbluh, J. *et al.* Complementary information derived from CRISPR Cas9 mediated gene deletion and suppression. *Nat. Commun.* **8**, 15403 (2017).
17. Cleary, B., Cong, L., Cheung, A., Lander, E. S. & Regev, A. Efficient Generation of Transcriptomic Profiles by Random Composite Measurements. *Cell* **171**, 1424-1436.e18 (2017).
18. Kuzmin, E. *et al.* Systematic analysis of complex genetic interactions. *Science* **360**, eaao1729 (2018).
19. Lukowski, S. W. *et al.* Genetic correlations reveal the shared genetic architecture of transcription in human peripheral blood. *Nat. Commun.* **8**, 483 (2017).

20. Astle, W. J. *et al.* The Allelic Landscape of Human Blood Cell Trait Variation and Links to Common Complex Disease. *Cell* **167**, 1415-1429.e19 (2016).
21. THE GTEX CONSORTIUM. The GTEx Consortium atlas of genetic regulatory effects across human tissues. *Science* **369**, 1318–1330 (2020).
22. Dahl, A., Guillemot, V., Mefford, J., Aschard, H. & Zaitlen, N. Adjusting for Principal Components of Molecular Phenotypes Induces Replicating False Positives. *Genetics* **211**, 1179–1189 (2019).
23. Umans, B. D., Battle, A. & Gilad, Y. Where Are the Disease-Associated eQTLs? *Trends Genet.* **37**, 109–124 (2021).
24. Leon, A. C. & Heo, M. Sample Sizes Required to Detect Interactions between Two Binary Fixed-Effects in a Mixed-Effects Linear Regression Model. *Comput. Stat. Data Anal.* **53**, 603–608 (2009).
25. Replogle, J. M. *et al.* Mapping information-rich genotype-phenotype landscapes with genome-scale Perturb-seq. *Cell* **185**, 2559-2575.e28 (2022).
