# Supplementary material for: Scalable genetic screening for regulatory circuits using compressed Perturb-seq
Source: Nat Biotechnol. 2023 Oct 23;42(8):1282–95. doi: 10.1038/s41587-023-01964-9 (PMC11035494; doi:10.1038/s41587-023-01964-9)
Supplement: Supplementary file 2 — Reporting Summary [file 41587_2023_1964_MOESM2_ESM.pdf]

nature portfolio

Corresponding author(s): Douglas Yao, Aviv Regev, Alexander Gusev, Brian Cleary

Last updated by author(s): Aug 20, 2023

Reporting Summary

Nature Portfolio wishes to improve the reproducibility of the work that we publish. This form provides structure for consistency and transparency in reporting. For further information on Nature Portfolio policies, see our Editorial Policies and the Editorial Policy Checklist.

Statistics

For all statistical analyses, confirm that the following items are present in the figure legend, table legend, main text, or Methods section.

n/a	Confirmed	
☐	☒	The exact sample size (n) for each experimental group/condition, given as a discrete number and unit of measurement
☒	☐	A statement on whether measurements were taken from distinct samples or whether the same sample was measured repeatedly
☐	☒	The statistical test(s) used AND whether they are one- or two-sided<br>*Only common tests should be described solely by name; describe more complex techniques in the Methods section.*
☐	☒	A description of all covariates tested
☐	☒	A description of any assumptions or corrections, such as tests of normality and adjustment for multiple comparisons
☐	☒	A full description of the statistical parameters including central tendency (e.g. means) or other basic estimates (e.g. regression coefficient) AND variation (e.g. standard deviation) or associated estimates of uncertainty (e.g. confidence intervals)
☐	☒	For null hypothesis testing, the test statistic (e.g. F, t, r) with confidence intervals, effect sizes, degrees of freedom and P value noted<br>*Give P values as exact values whenever suitable.*
☒	☐	For Bayesian analysis, information on the choice of priors and Markov chain Monte Carlo settings
☒	☐	For hierarchical and complex designs, identification of the appropriate level for tests and full reporting of outcomes
☐	☒	Estimates of effect sizes (e.g. Cohen's d, Pearson's r), indicating how they were calculated

Our web collection on statistics for biologists contains articles on many of the points above.

Software and code

Policy information about availability of computer code

Data collection	No software was used to collect data.
Data analysis	Starting with raw Illumina BCL files from the sequencing output, the "cellranger mkfastq" command with default parameters (from the 10x CellRanger tool v6.0.1; https://support.10xgenomics.com/single-cell-gene-expression/software/downloads/latest) was used to generate FASTQ files. To generate the droplet by perturbation design matrix, paired-end reads (in FASTQ format) containing a droplet barcode and UMI on read 1 and sgRNA sequence on read 2 were aligned using the Bowtie2 software (version 2.3.4.3). We used our custom software FR-Perturb (https://github.com/douglasyao/FR-Perturb) to estimate perturbation effect sizes from our data. Sc-linker (https://github.com/kkdey/GSSG) was used to compute disease enrichment of gene sets constructed from perturbation effects sizes. PLINK v1.90b6.4 was used to compute eQTL summary statistics. GCTA v1.93.0beta was used to compute genetic correlations. coloc v5.1.0 was used to compute cis-by-trans eQTLs.

For manuscripts utilizing custom algorithms or software that are central to the research but not yet described in published literature, software must be made available to editors and reviewers. We strongly encourage code deposition in a community repository (e.g. GitHub). See the Nature Portfolio guidelines for submitting code & software for further information.

Data

Policy information about availability of data

All manuscripts must include a data availability statement. This statement should provide the following information, where applicable:

- Accession codes, unique identifiers, or web links for publicly available datasets
- A description of any restrictions on data availability
- For clinical datasets or third party data, please ensure that the statement adheres to our policy

Raw and processed data for all screens were deposited in NCBI's Gene Expression Omnibus under accession number GSE221321. SNP-to-gene links (for running sc-linker) can be found at https://github.com/kkdey/GSSG. GWAS summary statistics can be found at https://data.broadinstitute.org/alkesgroup/sumstats_formatted/. eQTLGen data can be found at https://www.eqtlgen.org/phase1.html. Genotypes and expression data from the Fairfax et al. study can be found at the European Genome-phenome Archive (https://ega-archive.org/) under study ID EGAS00000000109, though approval is needed to obtain raw data. Gene sets from the Molecular Signatures Database used to run enrichment analysis can be found at https://www.gsea-msigdb.org/gsea/msigdb/collections.jsp.

Human research participants

Policy information about studies involving human research participants and Sex and Gender in Research.

Reporting on sex and gender	N/A
Population characteristics	N/A
Recruitment	N/A
Ethics oversight	N/A

Note that full information on the approval of the study protocol must also be provided in the manuscript.

Field-specific reporting

Please select the one below that is the best fit for your research. If you are not sure, read the appropriate sections before making your selection.

☒ Life sciences ☐ Behavioural & social sciences ☐ Ecological, evolutionary & environmental sciences

For a reference copy of the document with all sections, see nature.com/documents/nr-reporting-summary-flat.pdf

Life sciences study design

All studies must disclose on these points even when the disclosure is negative.

Sample size	Sample sizes for experiments were chosen so that each perturbation would be represented in roughly 100 cells. This number comes from previous Perturb-seq screens (Dixit et al. 2016 Cell).
Data exclusions	We excluded single-cell RNA-seq data from one 10X channel each from the conventional knock-out and conventional knock-down screen respectively due to unusual characteristics of the cells (very low sequencing coverage).
Replication	We experimentally validated several of the novel results found in our Perturb-seq screens, namely the effects of RAB5C, PGM3, XPR1, and KIDINS220 KO on the inflammatory response in LPS-stimulated THP1 cells, as measured by the secretion of IL6 from ELISA. All attempts at replication were successful when measuring IL6. We also attempted to validate the effects of KO of these genes on secretion of IFIT1 (as a proxy for the anti-viral response) from ELISA, but the baseline levels of IFIT1 protein were too low to be detected in control cells, so we excluded these results.
Randomization	Not applicable. Our study was conducted in a cell line, so confounders were controlled through controlling experimental conditions rather than randomization.
Blinding	Not applicable. Our study was conducted in a cell line, so group assignment bias does not occur.

Reporting for specific materials, systems and methods

We require information from authors about some types of materials, experimental systems and methods used in many studies. Here, indicate whether each material, system or method listed is relevant to your study. If you are not sure if a list item applies to your research, read the appropriate section before selecting a response.

Materials & experimental systems

n/a	Involved in the study
☒	☐ Antibodies
☐	☒ Eukaryotic cell lines
☒	☐ Palaeontology and archaeology
☒	☐ Animals and other organisms
☒	☐ Clinical data
☒	☐ Dual use research of concern

Methods

n/a	Involved in the study
☒	☐ ChIP-seq
☒	☐ Flow cytometry
☒	☐ MRI-based neuroimaging

Eukaryotic cell lines

Policy information about cell lines and Sex and Gender in Research

Cell line source(s)	Name: THP-1. Source: ATCC (product number : TIB-202). The cells are human, male.
Authentication	The cell line was not authenticated.
Mycoplasma contamination	Mycoplasma was tested once a month in the cell line, plus once upon receiving the new cells from ATCC. The cell line tested negative for mycoplasma each time.
Commonly misidentified lines (See ICLAC register)	The cell line is not commonly misidentified.
